# Primary lymphoma of the uterine cervix: A systematic review and integrated analysis of case reports and series

**DOI:** 10.3892/ol.2025.14896

**Published:** 2025-01-21

**Authors:** Konstantinos S. Kechagias, Stergios Bobotis, Amy Shearer, Laura Burney Ellis, Sophie M. Stephens, Sarah J. Bowden, Ilkka Kalliala, Foteini Kalofonou, Deirdre Lyons, Anita Mitra, Apostolia Galani, Maria Paraskevaidi, Maria Kyrgiou

**Affiliations:** 1 Department of Metabolism, Digestion and Reproduction, Faculty of Medicine, Imperial College London, London W12 ONN, UK; 2 Department of Obstetrics and Gynecology, Imperial College Healthcare NHS Trust, London W12 0HS, UK; 3 Department of Obstetrics and Gynecology, Helsinki University and University Hospital Helsinki, 00014 Helsinki, Finland; 4 Department of Medical Oncology, The Royal Marsden NHS Foundation Trust, London SW3 6JJ, UK

**Keywords:** lymphoma, uterine cervix cancer, systematic review

## Abstract

Evidence on the diagnosis and management of women with primary lymphoma of the uterine cervix (PLUC) is limited. The present study performed a systematic review of the literature and provided an overview of the reported cases of PLUC. A total of 213 reports were included, which comprised 339 patients with PLUC. The mean age of the patients was 48.5 years (median, 46 years; age range, 15–88 years). The most common presenting symptom was vaginal bleeding (189/318, 59.4%) and its duration ranged from 4.5 days to 24 months, with only a small fraction of patients developing ‘B’ symptoms including weight loss, night sweats and fever (28/318, 8.8%). Biopsy (either excisional or punch biopsy) was the most commonly used initial/primary diagnostic modality (78/278, 28.1%) followed by ultrasound (59/278, 21.2%). The most common management approach out of 309 patients was surgery (with or without adjuvant or neo-adjuvant treatment; 115/309), followed by chemotherapy alone (109/309), which was followed by chemo-radiotherapy alone (62/309). The follow-up period for survivors ranged from 4 weeks to 246 months, and the absolute 5-year and 10-year survival rates were 86.1 and 85.4%, respectively. The relatively low number of patients and high heterogeneity did not permit a robust comparative analysis of the survival outcomes. However, the longest median survival was reported for women who received neo-adjuvant chemotherapy followed by surgery (15 patients; 72 months). Although malignant PLUC is rare, early detection, optimal therapeutic management, and multidisciplinary involvement of gynecologic oncologists and lymphoma specialists may offer benefit to patients diagnosed with this rare disease.

## Introduction

Malignant lymphomas constitute a biologically heterogeneous group of diseases resulting from the clonal expansion of lymphocytes (1) and they are categorized based on their presumed cell of origin (either B-cell or T-cell), their morphology (Hodgkin or non-Hodgkin), and their level of cell differentiation (2). Approximately one fourth of all lymphomas originate from extranodal tissues, with the gastrointestinal tract and skin being the most common sites (3). Lymphomas that arise from the gynecological tract are less common, representing around 1% of extranodal lymphomas. Of these, less than 1% emerge from the uterine cervix (4).

The symptoms of primary lymphoma of the uterine cervix (PLUC) often mimic those of cervical epithelial tumors. A notable number of patients present with atypical symptoms such as bleeding and pain, and pelvic exam usually reveals cervical oedema. Since PLUC tend to develop in the cervical stroma rather than the mucosa, it has been suggested that cytology cannot serve as a reliable diagnostic tool, which creates a diagnostic challenge for clinicians (5). This characteristic limits the utility of traditional cytological screening methods, such as the Pap smear, which are designed to detect epithelial abnormalities. As a result, cytology often produces false negatives in PLUC cases and therefore has limited efficacy as an initial diagnostic tool. Given this, reliance on histopathological examination through biopsy and the use of imaging modalities, such as MRI and CT scans, is crucial for accurate diagnosis (6). PLUC bear histopathological resemblance to lymphomas that occur in other anatomical sites. However, the characterization of the malignancy as primary can be difficult because the disease often involves contiguous sites including the uterus and the vagina (6).

The optimal therapeutic management of patients with female genital tract lymphomas and PLUC comprise another matter of debate due to the rarity of the disease, the presence of different histopathological subtypes that respond differently to treatment and the lack of prospectively designed studies on the disease. According to a body of evidence which is mainly based on case reports and series, treatment options may encompass surgery, chemotherapy, radiotherapy, or various combinations of the above (7). Multidisciplinary co-operation between gynecologists, pathologists, hematologists and medical oncologists has been strongly recommended for the management of patients diagnosed with the disease.

Given the limited clinical data on PLUC, we sought to thoroughly review the existing literature and summarize the reported cases. Our focus was on the clinicopathological features, diagnosis, and treatment approaches associated with this condition.

## Materials and methods

### Registration

This review was reported based on the ‘Preferred Reporting Items for Systematic Reviews and Meta-Analyses’ (PRISMA) guidelines (8). The protocol was registered in PROSPERO (CRD42023424206).

### Literature search

Title screening was managed using Covidence. Two reviewers (SB, AS) independently searched PubMed, Web of Science and Scopus library databases from inception until August 2023. The search included the following terms: ‘Lymphoma’ AND (‘cerv*’ OR ‘vagina*’ OR ‘uter* OR ‘gynecological tract’). No restrictions regarding study design, geographic region or language were applied. To identify any overlooked studies, we also conducted a manual search of the references cited in the selected articles and relevant published reviews. Additionally, Google Scholar, tailored Google searches, and consultations with experts were employed to locate further articles from grey literature sources. Any discrepancies during the search process were addressed by involving a third investigator (KSK).

### Eligibility criteria

All study designs were considered eligible for inclusion only if they provided data for cases diagnosed with PLUC. The definition of PLUC was based on the definitions provided by the authors of the included studies (lymphoma presenting only in the uterine cervix with no visible lymphadenopathy on imaging or larger involvement of the uterine cervix in case of lymphoma presence on contiguous sites). Review articles, conference abstracts, and non-peer reviewed sources were not eligible for inclusion. We also excluded published dissertations, studies *in vitro* and animal models.

### Data extraction and handling

Non-English articles were manually translated when possible (9–15). In all studies, patient data was retrieved and handled by two authors (SB, AS) who conducted the data extraction independently. We collected the following information when available: Sex, age, menopausal status, comorbidities, family history of cancer, presenting symptoms and duration of symptoms, laboratory tests, primary diagnosis, histopathological markers, imaging findings, staging, therapeutic management, follow up time and clinical outcome (overall survival and disease-free survival). Any disagreements were discussed and resolved by a third investigator (KSK).

### Quality assessment

The risk of bias (RoB) was evaluated independently by two authors (SB, AS). To assess the overall quality of the case reports and case series, we utilized the critical appraisal checklist from the Joanna Briggs Institute (JBI) (16), which has been reliably employed in similar studies. The evaluation focused on eight key elements: patient demographics, medical history, current health status, physical examination and diagnosis, concurrent therapies, post-intervention health outcomes, and the interaction between drug administration and patient response. Each study was rated as ‘Yes’, ‘No’, or ‘Unclear/Not Applicable’ based on the availability and clarity of the information for each element.

### Data synthesis

This review employs descriptive statistics to present the demographics and clinical features, including symptoms, imaging methods, treatment approaches, staging, follow-up duration, and outcomes. Continuous variables are summarized using means, while binary variables are presented as rates or percentages. The duration of symptoms is reported as a range to account for the inconsistent and approximate reporting of this data across different studies.

Kaplan-Meier curves were generated to estimate overall survival irrespective of treatment used using GraphPad PRISM 9.0. Data was pooled when duration of follow up (in months) and outcome (alive or deceased) were provided. Duration of follow up was converted to months and the definition for overall survival was based on the definitions provided by the authors of the included studies. The patients were divided into different subgroups based on menopausal status, therapeutic management, hysterectomy type, use of adjuvant treatments and staging at diagnosis (supplementary material). Further sensitivity analyses were performed, to ensure homogeneity of data, excluding studies with high risk of bias, excluding pre-menopausal patients and excluding patients with high stage disease based on Ann Arbor staging system. Data with a P<0.05 was considered statistically significant. Missing and unidentifiable data were excluded from the statistical analysis. Both the log-rank test and the Gehan-Breslow-Wilcoxon method were used to compare the survival trend between the different subgroups.

## Results

### Study characteristics

**Figure 1.**
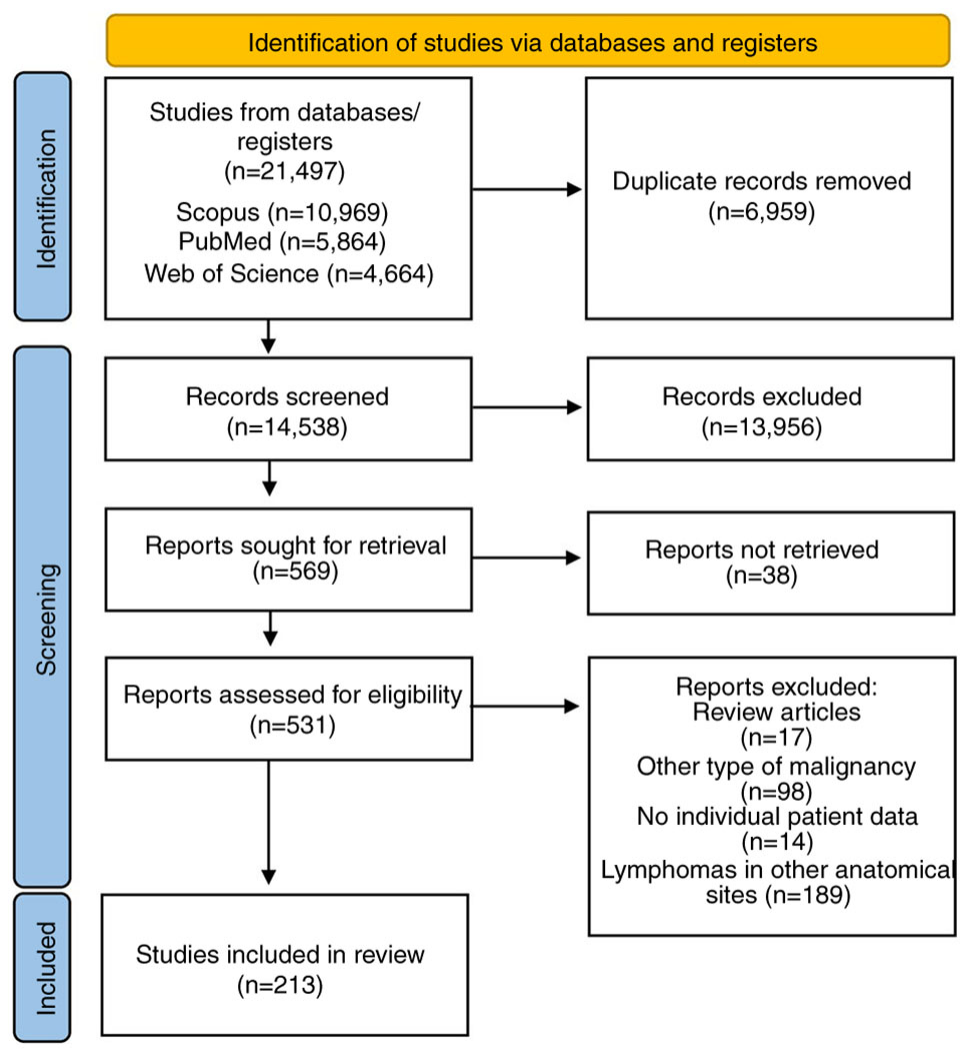
Preferred Reporting Items for Systematic Reviews and Meta-Analyses flowchart.

We identified 21,497 articles through the literature search; 6,959 bibliographic references were removed as duplicates and 13956 articles were excluded through title and abstract screening as irrelevant. Two authors reviewed 569 full text articles for eligibility. Finally, 213 studies, published between 1964 and 2023, were found eligible for the systematic review (Figs. 1 and S1). Regarding continent, 84 of the studies were conducted in Asia, 74 in Europe, 49 in Americas, 5 in Africa and 1 in Oceania. In terms of design, 162 studies were case reports and 51 were case series (Table SI).

### Patient characteristics

We identified a total of 339 cases of PLUC (Table SI). The mean age of the patients was 48.5 years (median: 46, age range: 15–88). Data regarding menopausal status was provided for 146 patients. For the remaining cases menopausal status was based on the age. In total, 129 patients were classified as premenopausal (129/339, 38.1%) and 129 as postmenopausal (129/339, 38.1%) (Table SI).

Data regarding symptomatology was available for 318 cases. The most common presenting symptom was vaginal bleeding (189/318, 59.4%) and its duration ranged from 4.5 days to 24 months. Vaginal bleeding was characterized in many cases as post-coital (33/3018, 10.4%) or inter-menstrual (12/318, 3.8%). The second most common symptom was vaginal discharge (44/318, 13.8%) followed by abdominal pain (34/318, 107) and pelvic pain (12/318, 3.8%). A few patients remained asymptomatic (42/318, 13.2%) and were diagnosed incidentally. Only a small fraction of patients developed ‘B’ symptoms (28/318, 8.8%) including night sweats (5/318, 1.5%), fever (9/318, 2.7%) and weight loss (14/318, 4.1%) (Tables SI and SII).

Although all cases were confirmed by histopathological examination, data regarding diagnostics was provided for 278 patients. Biopsy (either excisional or punch biopsy) was the most commonly used initial diagnostic modality (78/278, 28.1%) followed by ultrasound (59/278, 21.2%), cervical cytology (54/267, 20.2%) and computerized tomography (24/278, 8.6%). Data regarding histopathological types was available for all cases. Among the 339 cases analyzed, the most common histopathological subtype of PLUC was diffuse large B-cell lymphoma (DLBCL) (39/339). Other predominant subtypes included non-Hodgkin's B-cell lymphoma (13/339), large lymphoma (6/339), and intermediate lymphoma (6/339). Less common but notable types observed were lymphoma-like lesions (5/339) and several other rare subtypes, reflecting the histopathological diversity within PLUC. Data for histopathological markers was provided for 136 cases. The most commonly encountered markers included CD3 (61/136, 44.9%), CD10 (48/136, 35.3%), CD20 (15/136, 11%) cyclin D1 (34/136, 25%), CD5 (29/136, 21.3%) and BCL-2 (16/136, 11.8%) (Table SI).

For staging the Ann Arbor system was used in 179 patients. The most common stages included stage I (93/179, 52%) and stage II (47/179, 26.3%) followed by stage IV (29/179, 16.2%) and stage III (8/179, 4.5%). FIGO staging for cervical cancer was also utilized in 21 cases. The most common stages included FIGO stage I (12/21, 57.1%), FIGO stage II (4/21, 19%), FIGO stage III (1/21, 4.8%) and FIGO stage IV (4/21, 19%). Data regarding the presence of distant metastases at the time of diagnosis was provided for 245 patients with metastases being present in only a small fraction of patients (26/245, 10.6%) mainly affecting retroperitoneal lymph nodes and the kidney.

Information regarding management was provided for 309 patients (Table I). Almost one third of the patients were managed surgically (115/309, 35.5%), with most receiving adjuvant treatment (62/115), some receiving surgery alone (34/115), and a few receiving neo-adjuvant treatment (19/115). Of those that received surgery, the majority underwent hysterectomy (85/115); which included radical hysterectomy (9/115), total hysterectomy (58/85) and unspecified hysterectomy (18/85). A small number of patients underwent hysterectomy and lymphadenectomy (23/115). Of those that received neo-adjuvant treatment (19/115), the majority received neo-adjuvant chemotherapy (15/115), with a very small fraction of those receiving ‘R-CHOP’ chemotherapy (2/15), and the remaining receiving neo-adjuvant radiotherapy (4/19). More than half of those that underwent surgery received adjuvant treatment (62/115); the majority of this was chemotherapy (35/62), with a small amount receiving ‘R-CHOP’ chemotherapy (9/35), and the remainder receiving radiotherapy (18/62) or chemo-radiotherapy (9/62) (Table SIII).

The largest group of patients were managed without surgery and received only medical therapies (194/309). This comprised chemotherapy alone (109/194) with the commonest regime being ‘R-CHOP’, followed by chemo-radiotherapy (62/194), and radiotherapy (23/194).

### Management trends

**Figure 2.**
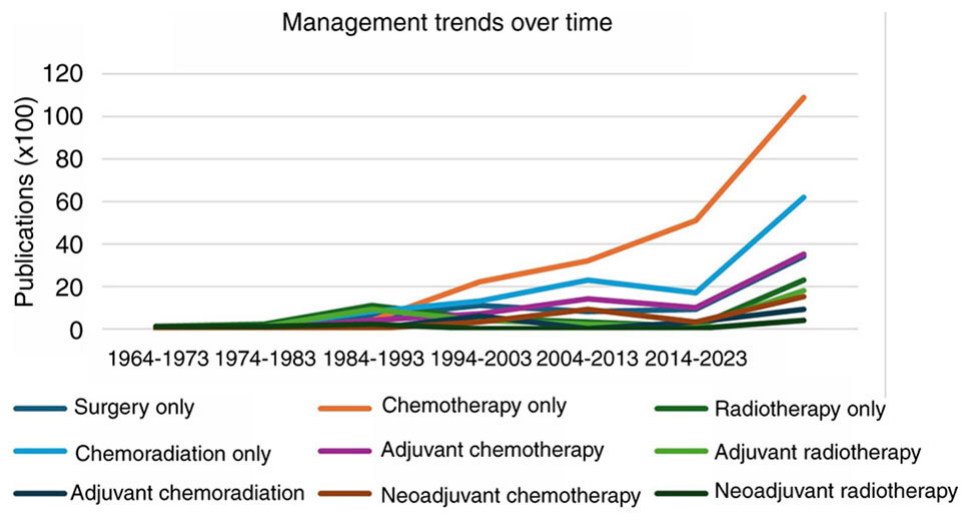
Management trends over time.

The majority of the cases with management data were published later than 1984 (301/309). By decade, between 1984 and present day, the proportion of patients treated with radiotherapy decreased from 25% (11/44) in 1984–1993, to 1% (1/95) in the years 2014–2023. Conversely, the number of patients managed with chemotherapy alone increased, from 9% (4/44) in the years 1984–1993, to 54% (53/95) in the most recent decade 2014–2023 (Fig. 2; Table SIV).

### Outcome and survival rates

**Figure 3.**
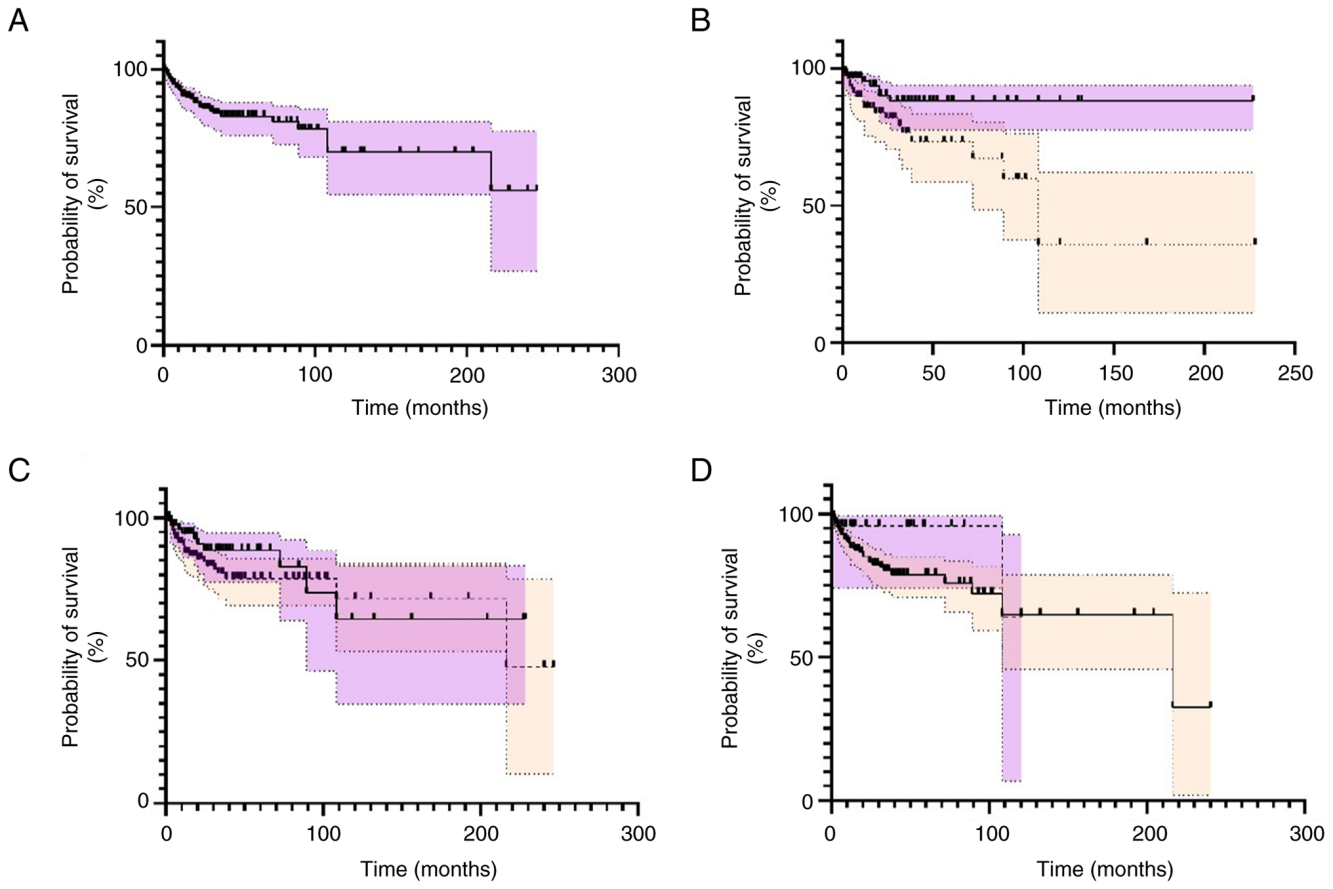
Kaplan-Meier curves calculated using the log-rank test and the Gehan-Breslow-Wilcoxon method for overall survival (A) for the total number of patients, and based on (B) menopausal status (premenopausal n=94 vs. postmenopausal n=80), (C) management type (operative n=78 vs. non-operative n=139) and (D) presence of symptoms (symptomatic n=187 vs. asymptomatic n=24). For all comparisons, the first group is illustrated in purple whereas the second group in beige.

Data for clinical outcome was provided for 279 patients, of whom 46 died and 233 survived. Almost half of the deaths occurred within 12 months after the initial diagnosis (21/45, 46.6%). The follow-up period for survivors ranged from 4 weeks to 246 months and the absolute 5-year and 10-year survival rates were 86.1 and 85.4%, respectively (Fig. 3A).

The longest median survival was for those that received chemotherapy alone (72 months, range 36–156), and particularly for the two patients that received neo-adjuvant ‘R-CHOP’ (96 months, range 36–156). The shortest median survival was for the three patients that received uterine artery embolization as treatment (5.75 months, range 1.5–10). Median survival for those who received any surgery (28 months, range 1.5–228) was not dissimilar to those who received surgery alone (30 months, range 6–24), any medical therapy alone (24 months, range 1–246), including specifically chemotherapy alone (23 months, range 3–168), radiotherapy alone (36 months, range 3–240), or chemo-radiotherapy (24 months, range 1–246). The type of hysterectomy did not appear to affect median survival; with median survival for radical hysterectomy (26 months, range 10–36) calculated to be similar to those that underwent hysterectomy and lymphadenectomy (27 months, range 4–108), total hysterectomy (28.5, range 2–228), or any unspecified hysterectomy (21.5 months, range 3–204) (Table I).

**Figure 4.**
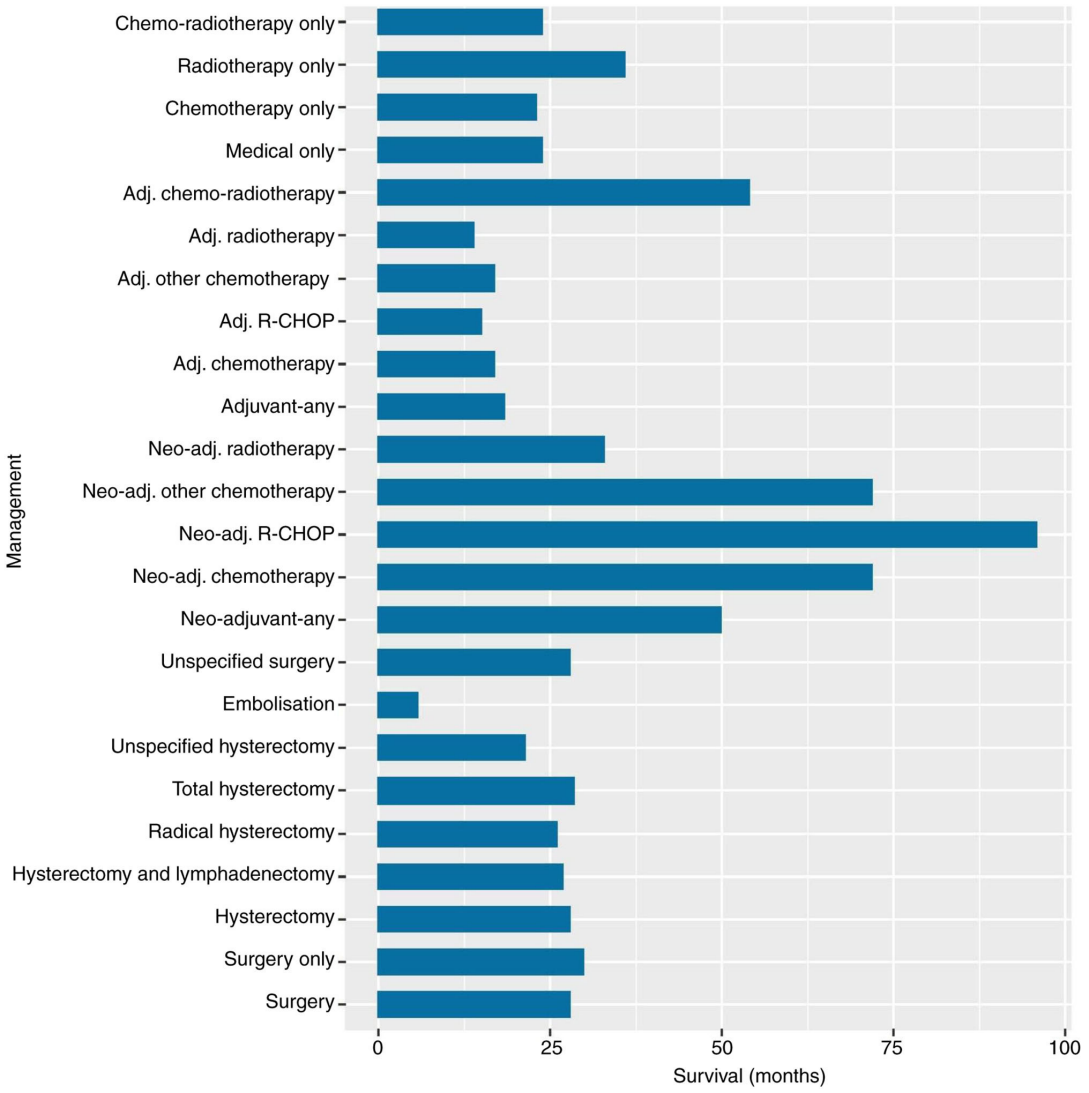
Median survival according to therapeutic management type.

Survival analysis indicated that premenopausal patients exhibited better prognosis compared to postmenopausal patients (P<0.0001) (Fig. 3B). Moreover, surgical management with or without adjuvant/neoadjuvant treatments was not superior to non-surgical management including chemotherapy, radiotherapy or chemoradiotherapy (P=0.29) (Fig. 2C). Additionally, we did not find statistically significant differences in overall survival when we compared surgery and adjuvant chemotherapy with chemotherapy alone as well as surgery and adjuvant chemoradiation with chemoradiation alone (Fig. S2; Table SIV). The presence of symptoms was not associated with a reduced overall survival rate (P=0.24) (Fig. 3D). The median survival and ranges for each management type are presented in Table I and displayed in Fig. 4.

### Quality of the studies

The quality assessment of the eligible studies indicated that the majority of the included studies (138/204) met most of the recommended criteria, categorizing them as having a low risk of bias. In contrast, 64 studies were classified with a medium risk of bias, while 2 studies fell into the high-risk category. A total of 20 studies achieved a perfect score. The aspect most frequently reported as ‘Not/Applicable’ among the studies was the patient's past medical history (Table SV, Table SVI, Table SVII). Sensitivity analyses were performed using the log-rank test and the Gehan-Breslow-Wilcoxon method while excluding potential confounding variables such as high risk of bias studies, premenopausal women, and patients with stages III and IV PLUC (Fig. S3, Fig. S4, Fig. S5). The survival curves produced show late-stage crossover which limits interpretation but have been included as part of sensitivity analysis.

## Discussion

In this systematic review of the literature, we identified cases of primary lymphoma of the uterine cervix and examined their clinicopathological characteristics, diagnosis and therapeutic management. Our study included 213 reports which comprised 339 patients diagnosed with primary lymphoma of the uterine cervix. Our findings revealed that most patients presented with non-specific symptoms including vaginal bleeding and discharge with a mean age of 48.5. The menopausal status of the patients varied and most of them were diagnosed at an early stage, according to the Ann Arbor staging system. The follow-up period ranged from 4 weeks to 246 months and the absolute 5- and 10-year survival rates were 86.1 and 85.4%, respectively. Although a higher concentration of cases was noted in Asia and Europe in terms of geographical distribution, the small volume of case reports and series analyzed does not allow the identification of any geographical variations or patterns. Broader studies on extranodal lymphomas suggest that factors such as genetic predispositions, environmental influences, or differences in healthcare systems and diagnostic practices may contribute to regional distribution patterns (3).

The existence and recognition of primary genital tract lymphomas was initially widely debated in the literature (3) until the identification of normal lymphoid tissue in female reproductive tract (17) and the reporting of patients with malignant lymphomas located exclusively in gynecological organs (18). While the exact causes and mechanisms behind these lymphomas remain elusive, chronic inflammation is suggested as a potential triggering factor (19). However, further mechanistic studies are required to elucidate the aforementioned theory. The increase in the incidence of extra-nodal lymphomas in different body sites, during the last decades has been also linked with the increase in immunosuppressive therapies, environmental toxins as well as, improved diagnostic techniques (20).

Despite the presence of published cases of primary lymphomas of the female genital tract in the scientific literature their definition remains a matter of controversy. A number of investigators have discussed cases of lymphomas in different parts of the female reproductive tract without presenting specific criteria to define primary tumors (5,21–23). Others have applied either the Fox and More criteria for uterine lymphoma (24) or the Harris and Scully modification of the Ann Arbor staging system for ovarian lymphomas (25). Currently, different staging systems are used for PLUC, which may contribute to inconsistencies in diagnosis and treatment planning. Developing a standardized staging and diagnostic framework specifically for PLUC would reduce this variability and facilitate more consistent management of the disease across clinical settings (26,27). Regarding PLUC, the definition that has been proposed by Kosari *et al* is the most comprehensive and includes the following criteria: i) Lymphomas confined to the cervix at the time of initial diagnosis, ii) lymphomas that are localized in the cervix without any evidence of involvement of other body parts on full investigation, and iii) the absence of abnormal cells indicating leukemia in the peripheral blood and bone marrow (28).

The presentation of the disease usually involves vaginal bleeding or discharge as cervical lesions can easily become ulcerated and/or infected (29), a phenomenon described in different histopathological types of cervical malignancies (30). In line with the results of our study, the presence of ‘B’ symptoms is considered uncommon and affects only a relatively small number of patients with PLUC (31), while cases of asymptomatic patients have been also reported. Despite the fact that clinical presentation of PLUC is very similar to other cervical tumors, the disease is considered to be human papillomavirus (HPV)-independent and distinct compared to cervical epithelial malignancies (32–36). While the co-existence of PLUC with other synchronous histological types of cervical cancer is pathophysiologically feasible, in this review, we only identified sporadic cases.

As far as diagnosis is concerned, histopathology plays a pivotal role while cytology cannot be utilized reliably as PLUC mainly affects the stroma rather than the epithelium of the uterine cervix. This was also reflected in our results, with only one fifth of the investigators using cytology as the first modality for diagnosis. The histopathological diversity within PLUC, with DLBCL as the most common subtype, mirrors patterns observed in other extranodal lymphomas (27,37). The range of subtypes suggests that while broad treatment protocols may be effective, subtype-specific approaches may be beneficial for optimizing outcomes. Less common subtypes, such as lymphoma-like lesions, further underscore the need for careful diagnostic differentiation, as histopathology plays a critical role in guiding treatment decisions (38). Future research should focus on identifying and characterizing genetic profiles in PLUC, as this could lead to more tailored and effective treatment options, similar to the advancements seen in other lymphomas.

Immunohistochemistry is another important diagnostic tool and lymphoma markers used for other types of lymphocyte malignancies can be also found consistently in PLUC. These marker include among others CD20 and Ki-67 for B-cell and CD3 and CD20 for T-cell lymphomas (37). Although such markers are widely used in diagnosing PLUC, variability in their expression across cases can impact diagnostic accuracy. This variability likely stems from the biological diversity of lymphomas, with different lymphocyte subtypes displaying distinct immunophenotypic profiles (26). Genetic mutations and the tumor microenvironment also contribute to these differences, highlighting the need for a more comprehensive marker panel to improve accuracy (38).

In the analysis it was evident that premenopausal women had better oncological outcomes when compared to the postmenopausal women. Although such an outcome could have been expected as premenopausal status could be associated with a younger, healthier population, the actual reasons for this disparity remain unclear. Potential factors may include differences in hormonal status, comorbidities, or variations in treatment response. Further research is necessary to investigate the underlying mechanisms and how they may influence survival outcomes in PLUC (38,39).

Due to the rarity of the disease, a consensus regarding therapeutic management has not been established. Older reports utilized radiotherapy as monotherapy. However, more recent approaches included either surgery (115/309), with or without neo-adjunctive or adjunctive therapy, chemotherapy alone (109/309), or a combination of chemotherapy and radiotherapy (62/310). In our analysis, there was no statistically significant difference in the overall survival when surgery alone was compared with chemotherapy alone. However, the longest calculated median survival was for those who received neo-adjuvant chemotherapy (15 patients, median survival 72 months, range 36–156). Additionally, surgery followed by adjunctive chemotherapy resulted in similar overall survival when compared to surgery alone. The question is whether PLUC should be managed in accordance with treatment guidelines for lymphoma of other sites (26), or whether it should be site-specific; an anatomical site such as the uterine cervix is feasible to remove surgically with relatively little morbidity. Our study does not provide sufficient evidence to confirm the idea that PLUC should be managed non-surgically. Based on the extremely limited present data, which may also be influenced by the age of some of the studies and geographical variation, there is possibly a tentative suggestion that a combination of neo-adjuvant chemotherapy and surgery may represent a reasonable treatment option.

Current guidelines for the management of non-Hodgkin lymphomas typically recommend chemotherapy as the first-line approach. Specifically, for diffuse B-cell lymphomas, it is preferable to utilize a doxorubicin-based regimen, such as a course of six to eight cycles of R-CHOP (rituximab, cyclophosphamide, vincristine, prednisolone) (40). In contrast, when dealing with indolent B-cell lymphomas, such as marginal zone lymphomas, there is no single standardized regimen that is universally recommended. Treatment options vary, ranging from rituximab alone to more intricate and complex combinations like R-hyper C-VAD (cyclophosphamide, vincristine, doxorubicin, and dexamethasone alternating with methotrexate and cytarabine) (41). The consideration of radiotherapy as a treatment option is typically reserved for instances of very low-stage lymphomas that exhibit localized characteristics and can be easily targeted. For example, this approach may be appropriate for early-stage follicular lymphoma, as indicated in the NICE guidelines (42). Treatment guidelines for squamous cell carcinoma or adenocarcinoma of the cervix recommend surgery in cases where the disease is at an early stage (43). In our study the overall survival was not significantly different in patients treated with chemotherapy alone. It is possible that chemotherapy alone represents a safe treatment option for younger premenopausal patients diagnosed at an early stage who wish to retain their fertility. Del Valle-Rubido *et al* published the only case included in this study where the patient received fertility-sparing management; a 28-year-old woman received ‘CHOP’ chemotherapy, after transplantation of the ovaries; this patient went on to survive at least 10 years and was still alive at the time of writing (9). However, the results of our analysis in this study, including the lack of difference in survival between management options, should be interpreted with caution considering the potential limitations associated with the analysis including the low number of patients, the heterogeneity between the included studies and the scarcity of events.

To the best of our knowledge, our study is the most comprehensive review on the reported cases of PLUC. Our findings present pooled data from 328 patients and highlight published data with quality assessment of included studies.

However, several limitations should be acknowledged. The inclusion of low-quality case reports and case series limits the validity and generalizability of our conclusions. These studies inherently carry a significant risk of bias, being particularly susceptible to overinterpretation and selection bias. Although many of the studies were rated as low risk of bias according to our assessment tool, their fundamental design flaws make them prone to publication bias, especially since negative findings are rarely reported. Consequently, the data presented, while potentially insightful, may not accurately reflect real-world outcomes. Additionally, although this study identifies various subtypes of PLUC, the limited data available restricts our ability to thoroughly examine the treatment response and prognosis for each subtype, as also reflected in the lack of statistical power of the Kaplan-Meier graphs. Given the rarity of PLUC and its subtypes, further research is needed to understand these subtype-specific outcomes, including recurrence rates and disease-free survival, which may guide more tailored treatment strategies. Quality of life metrics are also essential for providing insight into the impact of PLUC treatments in the long-term. Therefore, data from larger, prospectively designed studies are essential for drawing more reliable conclusions about optimizing diagnostic and therapeutic strategies for patients with PLUC.

A further limitation in the classification of PLUC arises from the concurrent use of the Ann Arbor staging system, which is traditionally used for lymphomas, and the FIGO staging system, which is applied to cervical cancers. The use of these two distinct staging systems can create inconsistencies and challenges in classification, which may impact treatment decisions and hinder efforts to further develop comparative studies. Developing a unified staging system specifically tailored to PLUC could improve clarity in both clinical practice and research, allowing for a more standardized approach to diagnosis and treatment planning.

Another notable limitation is our inability to estimate the effect size of outcomes and quantify uncertainty. It is noteworthy that a majority of clinical cases lack sufficient information for concentrating study results into a mean, median, or proportion with a confidence interval. This underscores the significance of initiatives aimed at standardizing and enhancing the quality of information reported in case reports. Overall, a pooled integrated analysis of case reports and series cannot replace evidence provided by clinical trials. Therefore, subject recruitment in rare diseases and personalized medicine represents a critical task in clinical research (40,41,43,44).

Finally, the conclusions drawn from our integrated analysis are inevitably constrained by the small number of patients included, the difficulty to assess homogeneity between studies and the inherent rarity of the events under investigation. Statistical significance is typically determined by observing patterns or outcomes that are unlikely to occur by chance alone (45). However, in the context of rare events, the occurrence itself is infrequent, making traditional measures of statistical analysis and significance less applicable. The late crossover of survival curves observed in some of the supplementary sensitivity analyses highlights a statistical manifestation of this key limitation. Overall, the scarcity of events can lead to challenges in sample size, limiting the statistical power to detect meaningful differences or associations (46–47).

In conclusion, malignant lymphomas can arise at unconventional anatomical sites. In the cervix uteri, the tumor appearance can vary and thus may be misdiagnosed with a primary epithelial malignancy. Although primary malignant lymphomas of the uterine cervix are infrequent, early detection, optimal therapeutic management, and multidisciplinary involvement may provide benefits to patients developing the disease. The knowledge of the rare occurrence of malignant lymphoma in the cervix, its unique characteristics and the increased awareness of clinicians can prevent clinical misdiagnoses and ultimately improve the clinical outcomes of patients developing this uncommon disease. While evidence on this condition remains limited, leveraging international registries could significantly strengthen the body of knowledge surrounding this rare malignancy.

## Supplementary Material

Supporting Data

Supporting Data

## Figures and Tables

**Table I.** Median survival according to therapeutic management type.

A, Surgery		

Management	n/N (%)	Median survival, months (range)
Surgery	115/309 (37.2)	28 (1.5–228)
Surgery only	34/309 (11.0)	30 (6–204)
Hysterectomy	85/115 (73.9)	28 (2–228)
Hysterectomy and lymphadenectomy	23/115 (20.0)	27 (4–108)
Radical hysterectomy	9/85 (10.6)	26 (10–60)
Total hysterectomy	58/85 (68.2)	28.5 (2–228)
Hysterectomy (unspecified)	18/85 (21.2)	21.5 (3–204)
Embolisation	3/115 (2.6)	5.75 (1.5–10)
Surgery (unspecified)	14/115 (12.2)	28 (6–156)

**B, Neo-adjuvant treatment**		

**Management**	**n/N (%)**	**Median survival, months (range)**

Neo-adjuvant	19/115 (16.5)	50 (8–156)
Chemotherapy	15/19 (78.9)	72 (36–156)
R-CHOP	2/15 (13.3)	96 (36–156)
Other chemotherapy	14/15 (93.3)	72 (36–132)
Radiotherapy	4/19 (21.1)	33 (8–40)

**C, Adjuvant treatment**		

**Management**	**n/N (%)**	**Median survival, months (range)**

Adjuvant	62/115 (53.9)	18.5 (1.5–228)
Chemotherapy	35/62 (56.5)	17 (1.5–228)
R-CHOP	9/35 (25.7)	15 (4–48)
Other chemotherapy	26/35 (74.3)	17 (1.5–228)
Radiotherapy	18/62 (29.0)	14 (2–60)
Chemo-radiotherapy	9/62 (14.5)	54 (10–89)

**D, Medical treatment only**		

**Management**	**n/N (%)**	**Median survival, months (range)**

Medical only	194/309 (62.8)	24 (1–246)
Chemotherapy only	109/194 (56.2)	23 (3–168)
Radiotherapy only	23/194 (11.9)	36 (3–240)
Combination therapy: Chemo-radiotherapy only	62/194 (32.0)	24 (1–246)

n/N, number of events/total number.

## Data Availability

The data generated in the present study are included in the figures and/or tables of this article.
