# Supplementary material for: Primary lymphoma of the uterine cervix: A systematic review and integrated analysis of case reports and series
Source: Oncol Lett. 2025 Jan 21;29(3):150. doi: 10.3892/ol.2025.14896 (PMC11783993; doi:10.3892/ol.2025.14896)

Figure S1. Research output (number of published articles and number of patients) related to primary lymphoma of the uterine cervix between 1963 and 2023.

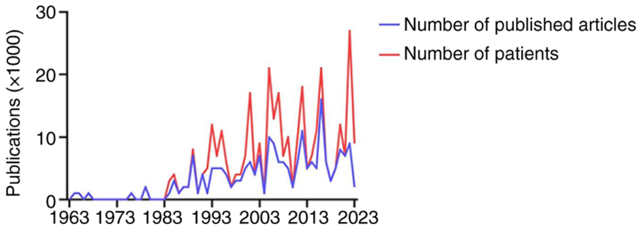

Figure S2. Kaplan-Meier curves calculated using the log-rank test and the Gehan-Breslow-Wilcoxon method for overall survival based on different management types including (A) operative vs. chemoradiation, (B) surgery + adjuvant treatment vs. chemoradiation, (C) surgery only vs. surgery + adjuvant treatment, (D) surgery + chemotherapy vs. chemotherapy only, (E) operative vs. chemotherapy only and (F) chemotherapy only vs. chemoradiation, and according to (G) presence of metastasis and stage, including (H) stage I+II vs. stage III+IV and (I) stage I vs. stage IV. For all comparisons, the first group is illustrated in purple whereas the second group in beige.

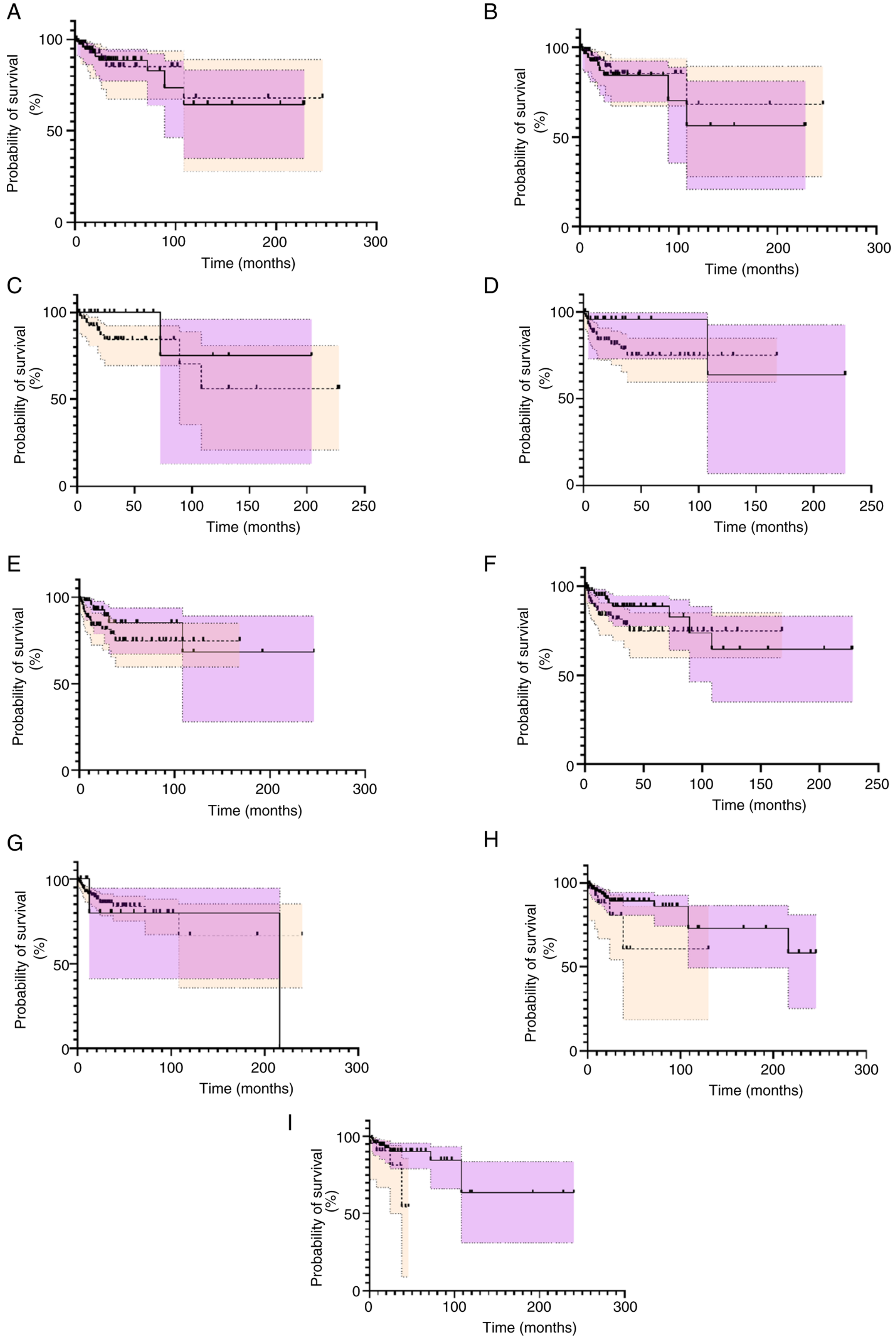

Figure S3. Kaplan-Meier curves calculated using the log-rank test and the Gehan-Breslow-Wilcoxon method excluding high RoB studies for overall survival. Based on (A) the total number of cases, (B) the menopausal status, (C) the presence of symptoms, based on different management types, including (D) operative management vs. non-operative management, (E) operative vs. chemoradiation, (F) surgery + adjuvant treatment vs. chemoradiation, (G) surgery only vs. surgery + adjuvant treatment, (H) surgery + chemotherapy vs. chemotherapy only, (I) operative vs. chemotherapy only and (J) chemotherapy only vs. chemoradiation, and according to (K) presence of metastasis and stage, including (L) stage I+II vs. stage III+IV and (M) stage I vs. stage IV. For all comparisons, the first group is illustrated in purple whereas the second group in beige.

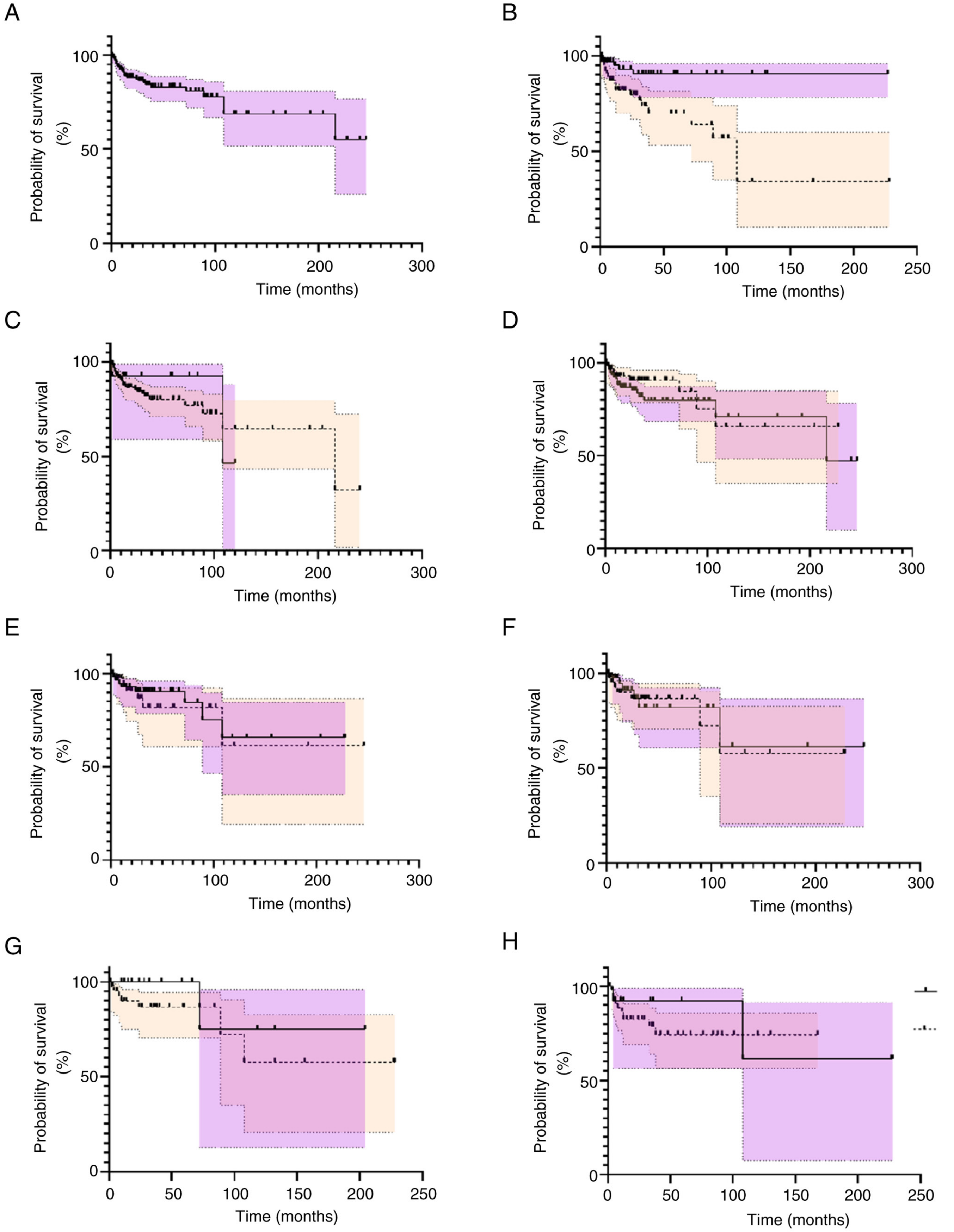

Figure S3. Continued.

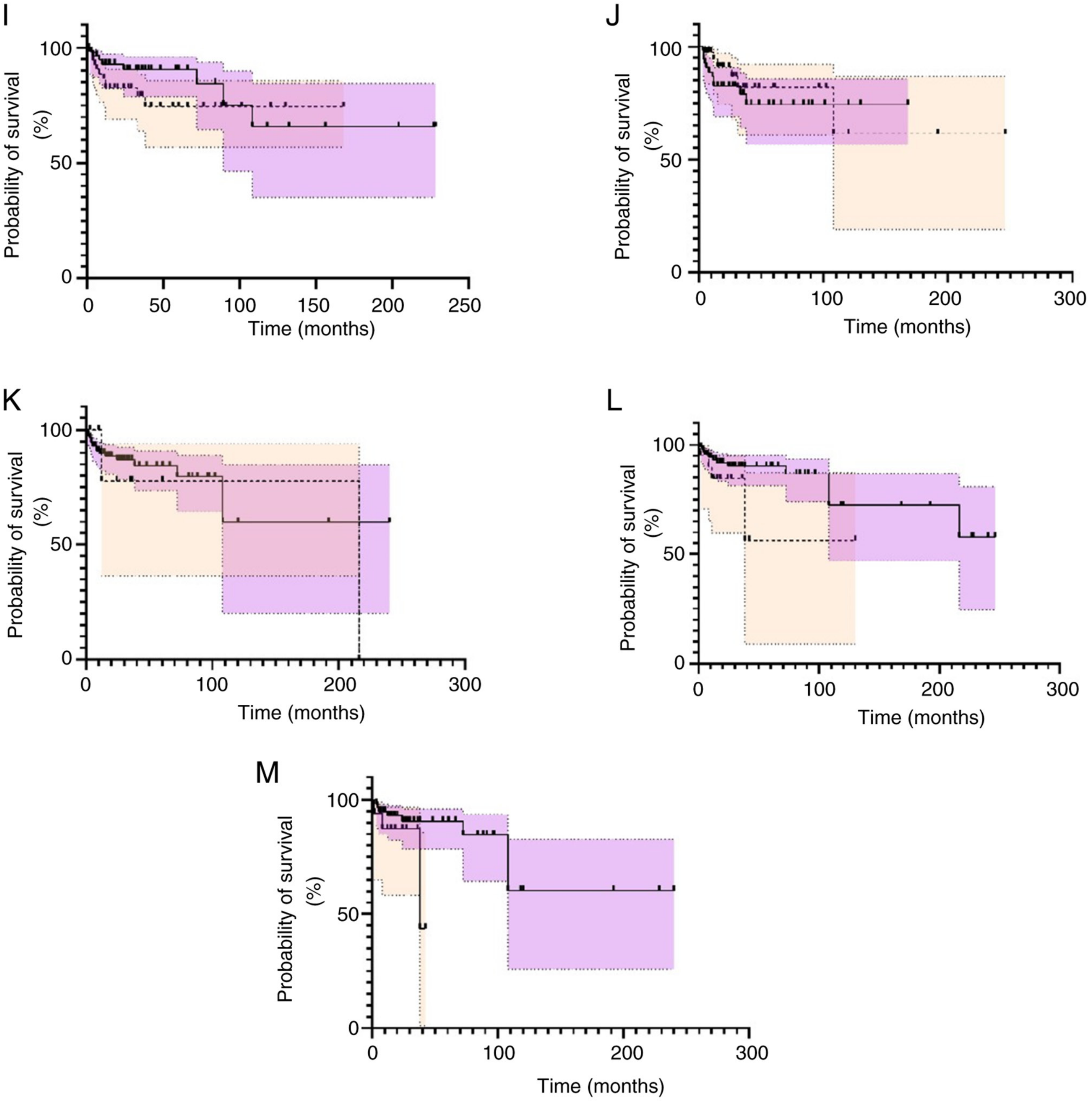

Figure S4. Kaplan-Meier curves calculated using the log-rank test and the Gehan-Breslow-Wilcoxon method excluding pre-menopausal women for overall survival. These were based on (A) the total number of cases, (B) the presence of symptoms, based on different management types, including (C) operative management vs. non-operative management, (D) operative vs. chemoradiation, (E) surgery + adjuvant treatment vs. chemoradiation, (F) surgery only vs. surgery + adjuvant treatment, (G) surgery + chemotherapy vs. chemotherapy only, (H) operative vs. chemotherapy only and (I) chemotherapy only vs. chemoradiation, and according to (J) presence of metastasis and stage, including (K) stage I+II vs. stage III+IV and (L) stage I vs. stage IV. For all comparisons, the first group is illustrated in purple whereas the second group in beige.

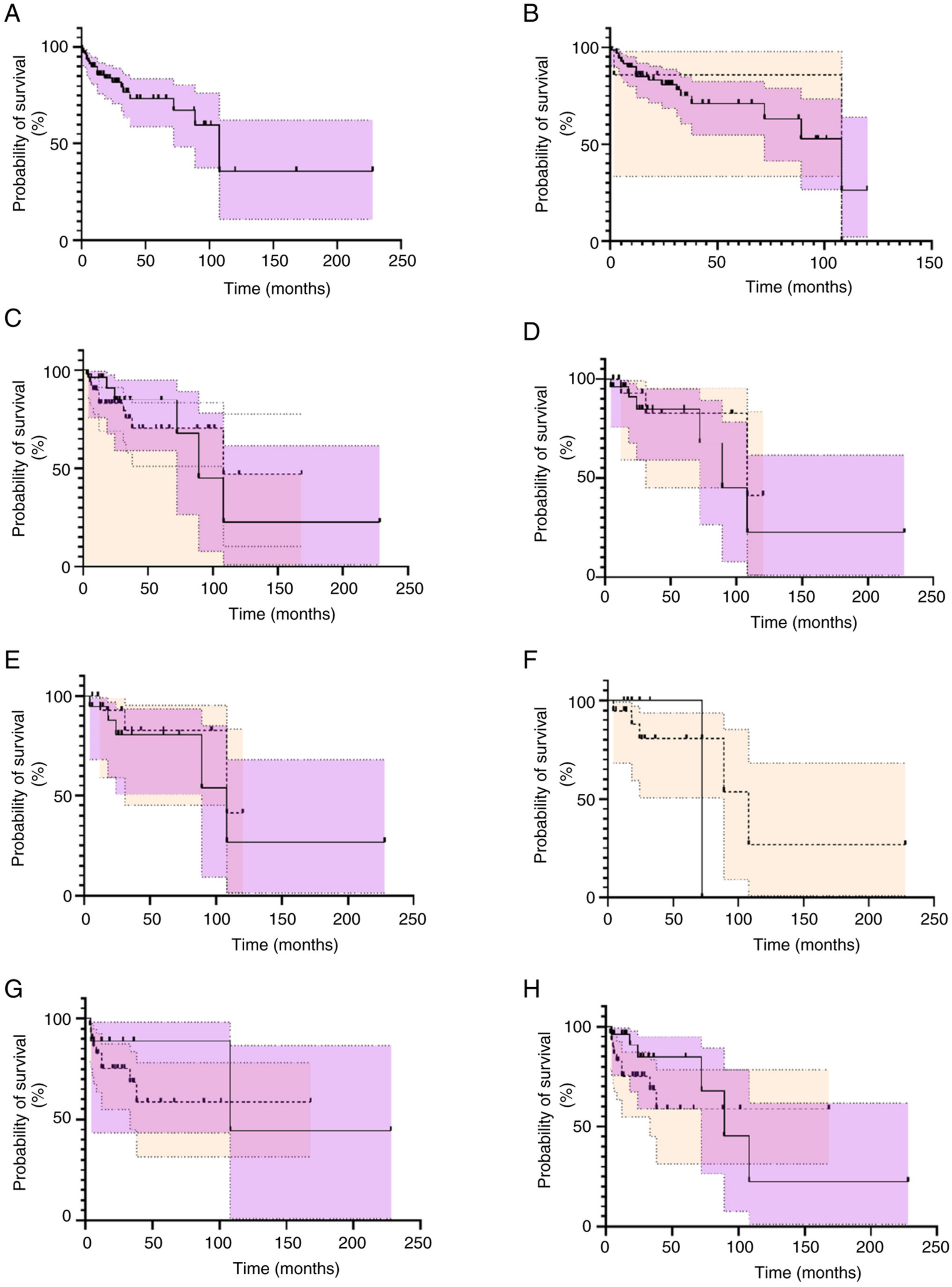

Figure S4. Continued.

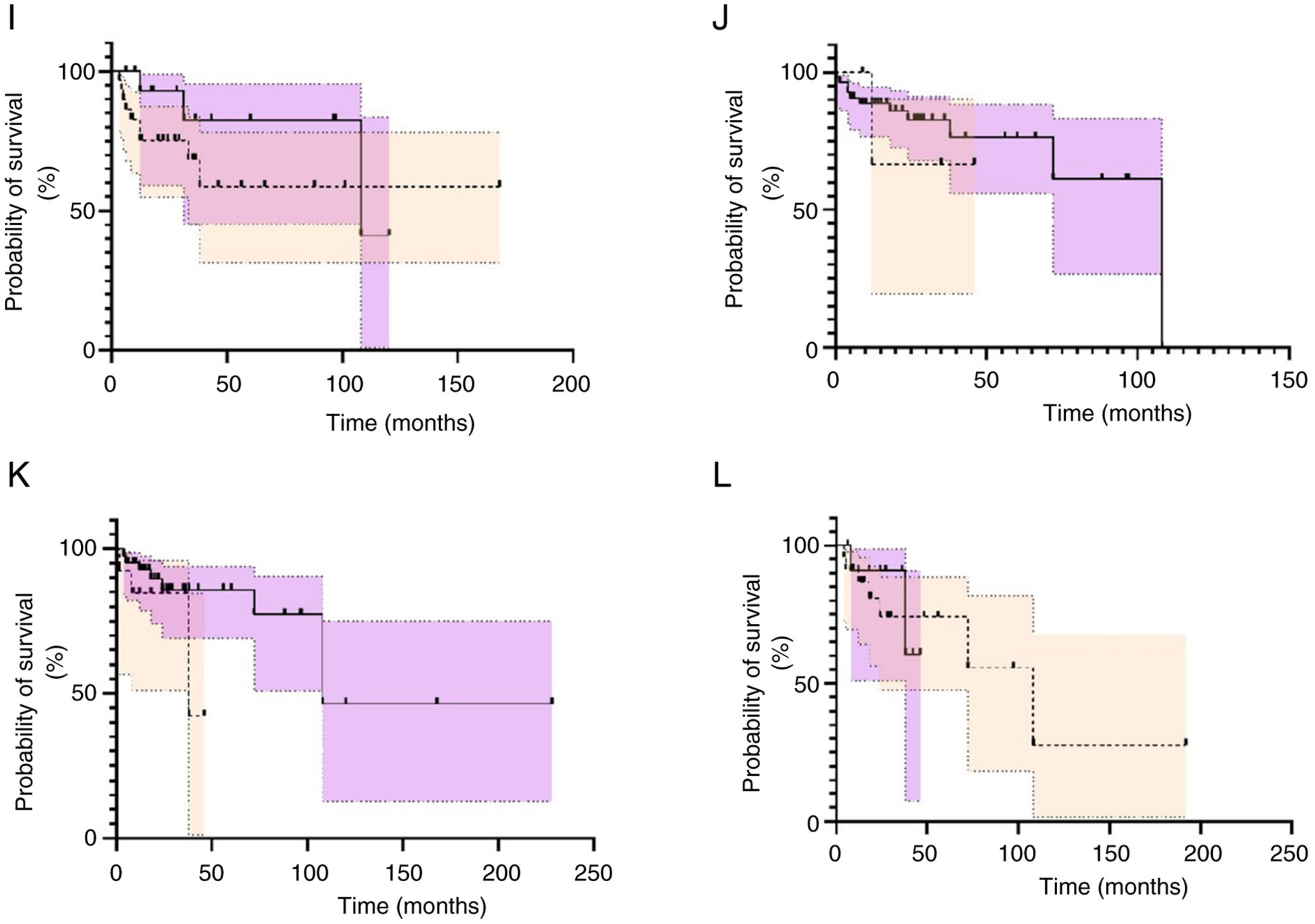

Figure S5. Kaplan-Meier curves calculated using the log-rank test and the Gehan-Breslow-Wilcoxon method excluding patients with Stage III and Stage IV for overall survival. Based on (A) the total number of cases, (B) the menopausal status, (C) the presence of symptoms, based on different management types, including (D) operative management vs. non-operative management, (E) operative vs. chemoradiation, (F) surgery + adjuvant treatment vs. chemoradiation, (G) surgery only vs. surgery + adjuvant treatment, (H) surgery + chemotherapy vs. chemotherapy only, (I) operative vs. chemotherapy only, (J) chemotherapy only vs. chemoradiation and according to (K) presence of metastasis. For all comparisons, the first group is illustrated in purple whereas the second group in beige.

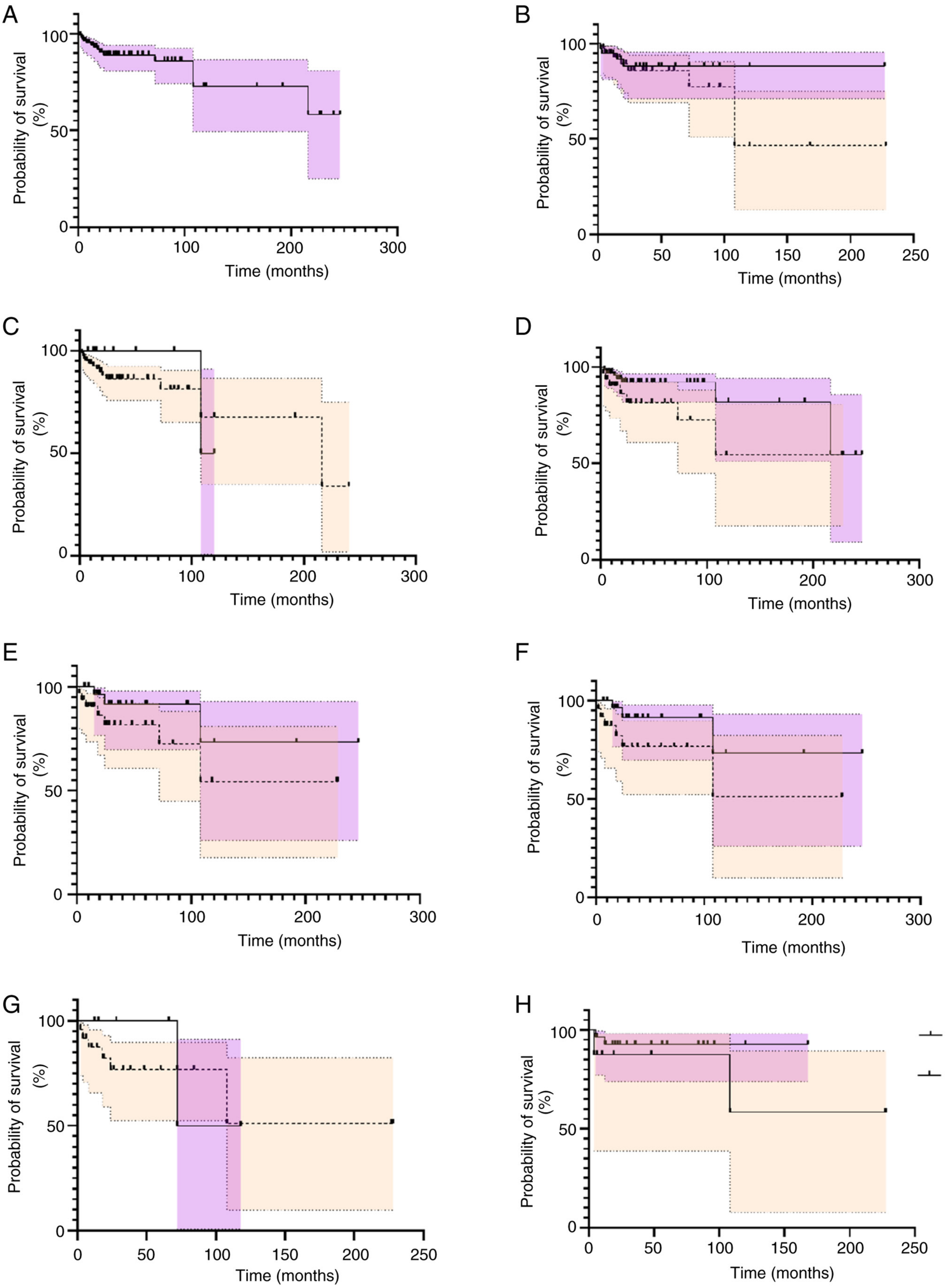

Figure S5. Continued.

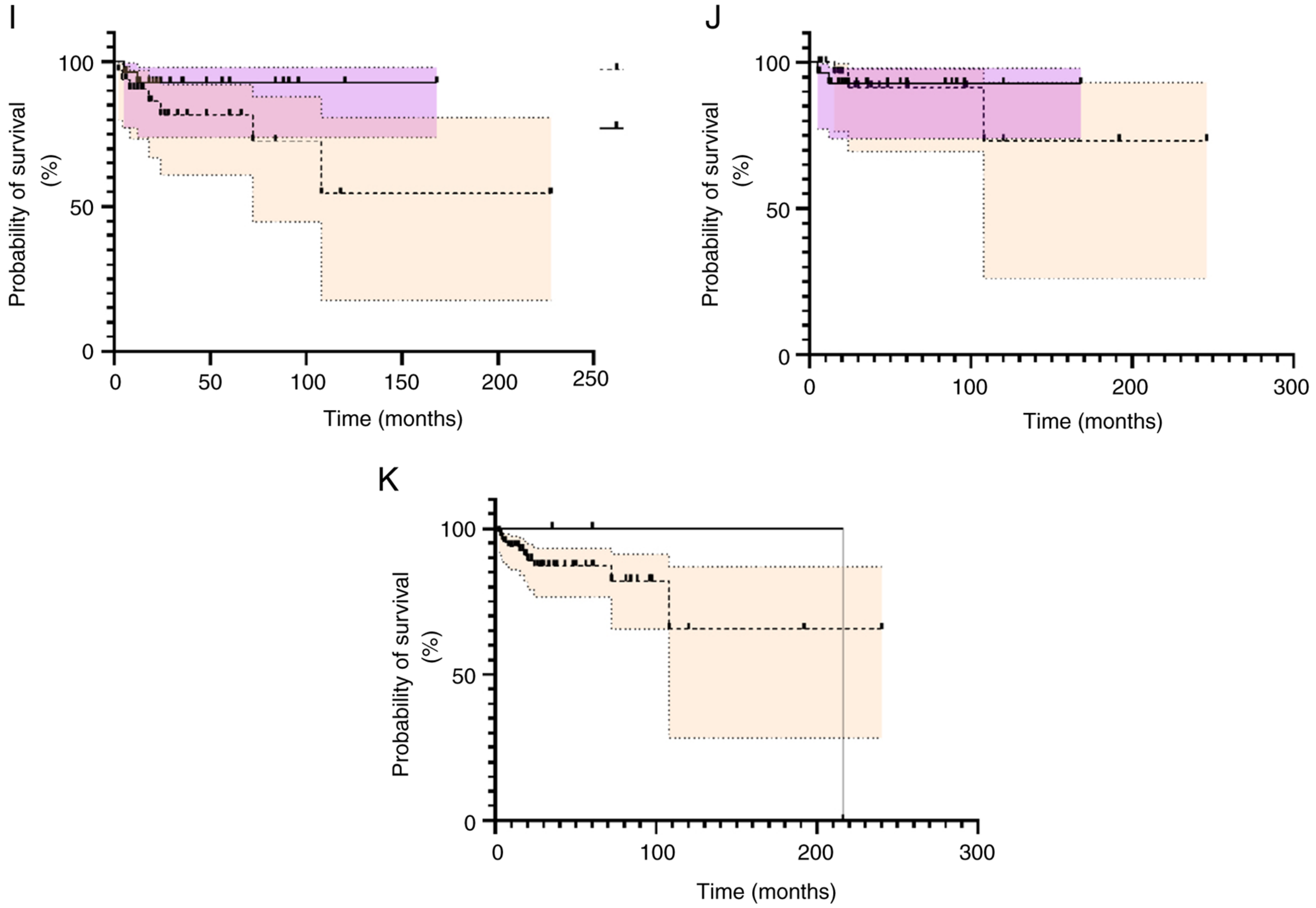

Supplement: Supporting Data [file Supplementary_Data1.pdf]
