# Supplementary material for: Primary lymphoma of the uterine cervix: A systematic review and integrated analysis of case reports and series
Source: Oncol Lett. 2025 Jan 21;29(3):150. doi: 10.3892/ol.2025.14896 (PMC11783993; doi:10.3892/ol.2025.14896)
Supplement: Supporting Data [file Supplementary_Data2.pdf]

Table SI. Characteristics of the included studies (individual patient data).

First author, year, country	Case	Age, years; menopausal status	Presentation (duration of symptoms)	First diagnostic modality	Histological type	Staging	Management	Follow-up (outcome)	(Refs.)
Nasiell[1] 1964 Sweden	Case 1	39 pre-menopausal	• Irregular vaginal bleeding	Vaginal and cervical smear	Hodgkin's lymphoma	n/a	• Initial: Radiotherapy, • Secondary: Total abdominal hysterectomy and bilateral salpingo-oophorectomy	8 months (alive)	(1)
Moores[2] 1965 UK	Case 1	49 peri-menopausal [a]	• Irregular vaginal bleeding (2 months)	Examination under anaesthesia	Primary lymphosarcoma	n/a	• Initial: Radiotherapy - pelvis, • Secondary: nil	n/a	(2)
Anderson[3] 1967 Hawaii	Case 1	40 peri-menopausal [a]	• Asymptomatic	Incidental-Histopathology of total abdominal hysterectomy and bilateral salpingo-oophorectomy	Hodgkins's lymphoma	n/a	• Initial: Total abdominal hysterectomy and bilateral salpingo-oophorectomy, • Secondary: Radiotherapy - cobalt external radiation therapy pelvis	12 months (alive)	(3)
Camino[4] 1974 Mexico	Case 1	29 pre-menopausal	• Dyspareunia (8 months) • Weight loss • Irregular vaginal bleeding (1 week)	Cervical biopsy	Cervical lymphoma	FIGO Stage IV	• Initial: Chemotherapy • Secondary: Radiotherapy – pelvis, paraaortic chain	1 month (deceased)	(4)
	Case 2	57 post-menopausal [a]	• Vaginal bleeding • Abdominal pain (7 months)	Cervical cytology	Cervical lymphoma	n/a	• Initial: Surgery • Secondary: Radiotherapy – pelvis, paraaortic chain	10 months (alive)	

Carr[5] 1976 UK	Case 1	44 peri-menopausal[a]	• Watery vaginal discharge<br>• Heavy menstrual periods<br>• Inter-menstrual bleeding<br>• Post-coital bleeding<br>(5 months)	Examination under anaesthesia and biopsy	Extranodal reticulum-cell type lymphoma	n/a	• Initial: Radiotherapy - pelvis and para-aortic nodes and cervix,<br>• Secondary: n/a	36 months (alive)	(5)
Tunca 1979 US	Case 1	22 pre-menopausal[a]	• Heavy vaginal bleeding	Cervical biopsy	Histiocytic lymphoma	n/a	• Initial: Radiotherapy - Cobalt-60 whole pelvis,<br>• Secondary: Total abdominal hysterectomy and bilateral salpingo-oophorectomy	n/a (alive)	(6)
Steinfeld 1979 USA	Case 1	29 pre-menopausal	• Post-coital bleeding<br>(5 days)	Cervical biopsy	Diffuse histiocytic lymphoma	IIE	• Initial: Radiotherapy - burnette applicator,<br>• Secondary: Radiotherapy - external beam therapy, Chemotherapy - vincristine	n/a	(7)
Komaki 1984 US	Case 1	43 peri-menopausal[a]	• Irregular menstrual periods<br>• Abdominal discomfort<br>• Pelvic discomfort<br>(36 months)	Cervical cytology	DLBCL	IIE	• Initial: Abdominal radiotherapy,<br>• Secondary: Radiotherapy - abdomen and supradiaphragmatic lymph nodes	81 months (alive)	(8)
	Case 2	38 pre-menopausal[a]	• Menorrhagia<br>• Low-back pain<br>(3 months)	Cervical cytology	DLBCL	IIE	• Initial: Radiotherapy - pelvis and para-aortic lymph nodes,<br>• Secondary: Radiotherapy - pelvis	84 months (alive)	(8)

	Case 3	38 pre-menopausal[a]	• Prolonged menstruation	Functional dilatation and curettage	DLBCL	IIE	• Initial: Radiotherapy - pelvis and para-aortic lymph nodes,<br>• Secondary: Radiotherapy - pelvis	36 months (alive)	(8)
Bowen[9] 1985 South Africa	Case 1	22 pre-menopausal[a]	• Brown-watery vaginal discharge (6 months)	IV pyelography	Large-cell lymphoma	IIE	• Initial: Chemotherapy - modified CHOP (cyclophosphamide and doxorubicin, vincristine, bleomycin, and prednisolone,<br>• Secondary: nil	n/a	(9)
Gharpure 1985 India	Case 1	50 peri-menopausal[a]	• Foul discharge<br>• irregular vaginal bleeding (2 months)	Cervical cytology	Diffuse poorly differentiated lymphocytic large-cell type lymphoma	n/a	• Initial: Radiotherapy,<br>• Secondary: nil	8 months (deceased)	(10)
	Case 2	45 peri-menopausal[a]	• Blood-stained vaginal discharge (1 month)	Cervical cytology	Diffuse histiocytic lymphoma	n/a	• Initial: Radiotherapy,<br>• Secondary: nil	6 months (deceased)	(10)
Taki 1985 Japan	Case 1	41 peri-menopausal[a]	• Irregular vaginal bleeding	Cervical cytology	Non-Hodgkin's B-cell lymphoma	n/a	• Initial: Simple total hysterectomy and bilateral salpingo-oophorectomy,<br>• Secondary: Radiotherapy - pelvis	n/a	(11)
Bar 1986 Netherlands	Case 1	66 post-menopausal[a]	• Uterine prolapse and vaginal bleeding	CXR	Non-Hodgkin's B-cell lymphoma	IE	• Initial: Hysterectomy, part of the parametrial tissue and pelvic lymph nodes,<br>• Secondary: Chemotherapy - CHOP	4 months (deceased)	(12)

Mann 1987 US	Case 1	44 peri-menopausal [a]	• Watery vaginal discharge	Cervical biopsy	Small-cell non-cleaved lymphoma	n/a	• Initial: Radiotherapy, • Secondary: Hysterectomy, Chemotherapy - F. Macop. (5-flurouracile, methotrexate, adriamycin, cytarabine, cyclophosphamide, vincristine, prednisolone)	10 months (deceased)	(13)
Cardillo 1987 Italy	Case 1	37 pre-menopausal	• Left submandibular swelling • Leucorrhoea • Inter-menstrual spotting	Cervical cytology	Low grade plasmacytoid non-Hodgkin's lymphoma	n/a	• Initial: Modified radical hysterectomy, bilateral salpingo-oophorectomy, pelvic and periaortic lymph node sampling, • Secondary: Radiotherapy - pelvis	40 months (alive)	(14)
Shimamoto 1987 Japan	Case 1	28 pre-menopausal	• Irregular bleeding (2 months)	Cervical cytology	Diffuse lymphoma	IIE, FIGO Ib	• Initial: Surgery – hysterectomy, pelvic, mesenteric, and omental lymph node sampling • Secondary: Chemotherapy – CHOP	8 months (alive)	(15)
Strang 1988 Sweden	Case 1	77 post-menopausal	• Post-menopausal bleeding	Cystoscopy	Aneuploid lymphoma	n/a	• Initial: Hysterectomy, • Secondary: nil	36 months (alive)	(16)
Ibrahim 1988 UK	Case 1	78 post-menopausal	• Intermittent vaginal bleeding (3 months)	Cervical cytology	Diffuse non-Hodgkin's lymphoma large cell type	n/a	• Initial: Radiotherapy - box technique, • Secondary: n/a	24 months (alive)	(17)
Khong 1989 Singapore	Case 1	54 peri-menopausal [a]	• Irregular heavy vaginal bleeding (2 months)	Cervical cytology	DLBCL	n/a	• Initial: Surgery, • Secondary: Chemotherapy - CHOP-Mtx	n/a (alive)	(18)

							(cyclophosphamide, doxorubicin, vincristine, prednisolone, methotrexate)		
Khoury 1989 UK	Case 1	37 pre-menopausal [a]	• Menorrhagia	Examination under anaesthesia	Non-Hodgkin's B-cell lymphoma	n/a	• Initial: Radiotherapy - pelvis, • Secondary: Laparotomy and excision of residual pelvic mass	96 months (alive)	(19)
	Case 2	46 peri-menopausal [a]	• Irregular vaginal bleeding (2 months)	Cystoscopy	Non-Hodgkin's B-cell lymphoma	n/a	• Initial: Total hysterectomy and bilateral salpingo-oophorectomy, • Secondary: Radiotherapy	102 months (alive)	(19)
Kurup 1989 Poland	Case 1	53 peri-menopausal [a]	• Irregular vaginal bleeding aggravated by coitus (3 weeks)	Cervical cytology	DLBCL	n/a	• Initial: Radiotherapy - Cobalt 60 pelvis and para-aortic area, • Secondary: Chemotherapy - COP	n/a	(20)
Hashimoto 1989 Japan	Case 1	45 pre-menopausal	• Vaginal bleeding (3 months)	MRI	DLBCL	IIAE	• Initial: Chemotherapy – R-CHOP • Secondary: nil	12 months (alive)	(21)
Campell 1989 UK	Case 1	32 pre-menopausal	• Pelvic discomfort • Frequent menstruation (2 months)	Ultrasound	Diffuse non-Hodgkin's lymphoma	n/a	• Initial: Chemotherapy - CHOP, • Secondary: Radiotherapy - pelvis	6 months (alive)	(22)
Johnston 1989 USA	Case 1	22 pre-menopausal	• Malodorous vaginal discharge • Post-coital bleeding • Irregular menstruation (2 months)	Cervical cytology	Diffuse histiocytic lymphoma of B-Cell type	IE	• Initial: Total abdominal hysterectomy and bilateral salpingo-oophorectomy and pelvic node sampling,	33 months (alive)	(23)

							• Secondary: Radiotherapy - external radiation		
Matsuyama 1989 Japan	Case 1	69 post-menopausal	• Post-menopausal bleeding (3 months)	Cervical cytology	Large cell-type lymphoma	IIE	• Initial: Radical hysterectomy with pelvic lymph node dissection and paraaortic lymph node biopsy, • Secondary: Radiotherapy - pelvis	n/a	(24)
Murotsuki 1989 Japan	Case 1	85 post-menopausal	• Vaginal bleeding (1 month)	Cervical biopsy	Small, cleaved, diffuse B-cell lymphoma	IE, FIGO Ib	• Initial: Total hysterectomy and bilateral salpingo-oophorectomy • Secondary: nil	1 year (alive)	(25)
Stickelmann 1989 Germany	Case 1	65 post-menopausal	• Post-menopausal bleeding	Cervical cytology	Highly non-Hodgkin's lymphoma- B-cell series (centroblastic lymphoma)	I	• Initial: Radiotherapy - Cobalt 60 pelvis and parametria, • Secondary: Radiotherapy - radium	9 months (alive)	(26)
Mathiasen 1990 Denmark	Case 1	22 pre-menopausal	• Vaginal bleeding (2 months)	Cervical cytology	Centroblastic diffuse B-cell lymphoma	n/a	• Initial: Radical hysterectomy and BSO and para-aortic lymph nodes and mesenteric lymph nodes, • Secondary: Chemotherapy - cisplatin, VP-16, bleomycin, prednisolone	34 months (alive)	(27)

Dang 1991 USA	Case 1	35 pre-menopausal [a]	• Vaginal bleeding with clots (7 months)	Cervical cytology	Intermediate grade non-Hodgkin's B-cell lymphoma	n/a	• Initial: Radical abdominal hysterectomy, upper vaginectomy and right salpingo-oophorectomy with right pelvic and periaortic lymph node sampling,<br>• Secondary: n/a	n/a	(28)
Hachisuga 1991 Japan	Case 1	60 post-menopausal [a]	• Post-menopausal bleeding	Cervical cytology	Lymphoma-like lesion	n/a	• Initial: Radical abdominal hysterectomy, upper vaginectomy and right salpingo-oophorectomy with right pelvic and periaortic lymph node sampling,<br>• Secondary: n/a	32 months (alive)	(29)
Pasini 1991 Italy	Case 1	35 pre-menopausal [a]	• Post-coital bleeding (24 months)	Colposcopy	Follicular centre cell derived B-cell lymphoma	IIE	• Initial: Chemotherapy - vincristine, adriamycin, cyclophosphamide, VP16, prednisolone,<br>• Secondary: n/a	12 months (alive)	(30)
Malatskey 1991 USA	Case 1	45 peri-menopausal [a]	• Inter-menstrual bleeding	Ultrasound	Large cell B-cell lymphoma	n/a	• Initial: Total hysterectomy and bilateral salpingo-oophorectomy,<br>• Secondary: nil	n/a	(31)
Perren 1992 UK	Case 1	54 peri-menopausal [a]	• Irregular vaginal bleeding (24 months)	Examination under anaesthesia and cervical biopsy	Diffuse small cleaved cells and large cell lymphoma without sclerosis	IIEA	• Initial: Radiotherapy,<br>• Secondary: n/a	216 months (deceased)	(32)

	Case 2	47 peri-menopausal [a]	• Pre-menstrual vaginal discharge<br>• Malaise<br>• Weight loss<br>• Night sweats (9 months)	Examination under anaesthesia cervical biopsy endometrial curettage	Diffuse small cleaved cells and large cell lymphoma without sclerosis	IIEB	• Initial: Chemotherapy - CHOP,<br>• Secondary: n/a	60 months (alive)	(32)
	Case 3	53 peri-menopausal [a]	• Urinary frequency and hesitancy (3 months)	Examination under anaesthesia vaginal biopsy	Diffuse large cleaved cell lymphoma without sclerosis	IEA	• Initial: Radiotherapy - whole pelvis,<br>• Secondary: n/a	240 months (alive)	(32)
	Case 4	41 peri-menopausal [a]	• Offensive vaginal discharge (1 month)	Examination under anaesthesia and biopsy	Diffuse small cleaved cells and large cell lymphoma with some sclerosis	IEA	• Initial: Chemotherapy - CHOP,<br>• Secondary: Radiotherapy - pelvis and two caesium insertions	96 months (alive)	(32)
	Case 5	35 pre-menopausal	• Post-coital bleeding (3 months)<br>• Right loin pain (1 month) (4 months)	Examination under anaesthesia and biopsy	Diffuse lymphoma with marked sclerosis	IEA	• Initial: Radiotherapy - external beam pelvis and para-aortic nodes,<br>• Secondary: Chemotherapy - cyclophosphamide, vincristine, prednisolone	38 months (alive)	(32)
Maryniak 1993 Poland	Case 1	72 post-menopausal	• Vaginal bleeding	n/a	High grade B -cell lymphoma	IE	• Initial: Chemotherapy - mitomycin C (peritoneal injection),<br>• Secondary: Radiotherapy	18 months (deceased)	(33)
	Case 2	53 post-menopausal	• Pelvic discomfort	n/a	High-grade B-cell lymphoma	IIE	• Initial: Total hysterectomy,<br>• Secondary: Radiotherapy	38 months (alive)	(33)
	Case 3	24	• Vaginal bleeding	n/a	Low grade B-cell lymphoma	IIE	• Initial: Total hysterectomy and	20 months	(33)

		pre-menopausal					bilateral salpingo-oophorectomy, • Secondary: Radiotherapy	(deceased)	
Aozasa 1993 Japan	Case 1	71 post-menopausal [a]	• Genital bleeding	n/a	DLBCL	NA	• Initial: Surgery, • Secondary: Radiotherapy	12 months (deceased)	(34)
	Case 2	30 pre-menopausal [a]	• Genital bleeding	n/a	Diffuse large predominantly noncleaved cell	II	• Initial: Chemotherapy - CHOP-MTX, • Secondary: nil	2 months (deceased)	(34)
	Case 3	41 peri-menopausal [a]	• Genital bleeding	n/a	Diffuse large predominantly cleaved cell type	II	• Initial: Chemotherapy - CHOP, • Secondary: Radiotherapy - Cobalt 60 external whole pelvis	8 months (deceased)	(34)
	Case 4	71 post-menopausal [a]	• Genital bleeding	n/a	Diffuse mixed small and large cell lymphoma	I	• Initial: Total abdominal hysterectomy and bilateral salpingo-oophorectomy and pelvic lymph node dissection, • Secondary: nil	24 months (deceased)	(34)
Broekmans 1993 Netherlands	Case 1	45 pre-menopausal	• Slight post-coital bleeding	Cervical cytology	Non-Hodgkin's B-cell lymphoma	IE	• Initial: Chemotherapy - CHOP, • Secondary: nil	36 months (alive)	(35)
Makarewicz 1993 Poland	Case 1	37 pre-menopausal	• Heavy menstrual bleeding • Post-coital bleeding	Cervical biopsy	Centroblastic B-cell non-Hodgkin's lymphoma	IEA	• Initial: Hysterectomy, • Secondary: Radiotherapy	96 months (alive)	(36)

	Case 2	33 pre-menopausal	• Offensive vaginal discharge<br>• Post-coital bleeding	Cervical biopsy	Centroblastic centrocytic diffuse B-cell non-Hodgkin's lymphoma	n/a	• Initial: Radiotherapy - external beam whole pelvis,<br>• Secondary: nil	42 months (alive)	(36)
	Case 3	65 post-menopausal	• Post-menopausal bleeding	Cervical biopsy	Lymphoblastic B-cell lymphoma	IEA	• Initial: Radiotherapy,<br>• Secondary: nil	36 months (alive)	(36)
Rodier 1993 France	Case 1	55 post-menopausal	• Post-menopausal bleeding (3 months)	Cervical biopsy	Non-Hodgkin's B-cell lymphoma type B	n/a	• Initial: Chemotherapy,<br>• Secondary: Radiotherapy	n/a (alive)	(37)
Kasales 1994 USA	Case 1	20 pre-menopausal [a]	• Vaginal bleeding	Cervical biopsy	Anaplastic large-cell lymphoma	n/a	• Initial: Radiotherapy - external whole pelvis regional cobalt irradiation,<br>• Secondary: n/a	8 months (alive)	(38)
	Case 2	31 pre-menopausal [a]	• Deep pelvic pain<br>• Vaginal bleeding	Cervical biopsy	Mixed histiocytic lymphoma	n/a	• Initial: Radiotherapy - pelvis,<br>• Secondary: nil	36 months (alive)	(38)
	Case 3	53 peri-menopausal [a]	• 10-pound weight gain TV	CT pelvis	Mixed small and large cell non-Hodgkin's lymphoma	n/a	• Initial: Radiotherapy - external pelvis,<br>• Secondary: Radiotherapy - Cobalt 60 high dose intracavitary	72 months (alive)	(38)
Cuykx 1994 Belgium	Case 1	40 peri-menopausal [a]	• Intermenstrual bleeding (5 months)	Ultrasound	Extranodal non-Hodgkin's B-cell lymphoma	n/a	• Initial: Chemotherapy - CHOP,<br>• Secondary: Radiotherapy	12 months (alive)	(39)
Awwad 1994 Lebanon	Case 1	27 pre-menopausal	• Post-coital bleeding<br>• Irregular menses (3 months)	Cervical biopsy	Lymphoma	IIA	• Initial: Chemotherapy - pro-MACE and CytoBOM,<br>• Secondary: nil	n/a	(40)

Huh 1994 South Korea	Case 1	36 pre-menopausal	• Irregular vaginal bleeding<br>• Pelvic discomfort (3 months)	Cervical cytology	Intermediate grade diffuse small cleaved cell lymphoma	IEA	• Initial: Supracervical hysterectomy and bilateral salpingo-oophorectomy,<br>• Secondary: Chemotherapy - CHOP, Radiotherapy	48 months (alive)	(41)
Patsner 1994 USA	Case 1	38 pre-menopausal	• Irregular vaginal bleeding	Cervical cytology	cervical non-Hodgkin's B-cell lymphoma	IE	• Initial: Leuprolide acetate,<br>• Secondary: Chemotherapy - CHOP, Radiotherapy - whole pelvis	36 months (alive)	(42)
Makarewicz 1995 Poland	Case 1	37 pre-menopausal [a]	• Menorrhagia<br>• Post-coital bleeding	Cervical biopsy	DLBCL	IEA	• Initial: Emergency hysterectomy and left salpingo-oophorectomy,<br>• Secondary: Radiotherapy	36 months (alive)	(43)
	Case 2	33 pre-menopausal [a]	• Offensive vaginal discharge<br>• Post-coital bleeding	Cervical biopsy	DLBCL	n/a	• Initial: Radiotherapy,<br>• Secondary: n/a	42 months (alive)	(43)
	Case 3	65 post-menopausal [a]	• Postmenopausal bleeding	Cervical biopsy	B-cell lymphoblastic lymphoma	IEA	• Initial: Hysterectomy,<br>• Secondary: Radiotherapy	96 months (alive)	(43)
Kawakami 1995 USA	Case 1	60 post-menopausal [a]	• Asymptomatic	Cervical cytology	Pleomorphic medium sized and large cell non-Hodgkin's B-cell lymphoma	n/a	• Initial: Chemotherapy,<br>• Secondary: n/a	n/a	(44)
	Case 2	37 pre-menopausal	• Abnormal vaginal bleeding (1 month)	Pelvic ultrasound	Non-Hodgkins T-cell lymphoma	n/a	• Initial: Chemotherapy,<br>• Secondary: n/a	n/a	(44)
	Case 3	63 post-menopausal [a]	• Abnormal vaginal bleeding (2 months)	Cervical biopsy	Small lymphocytic non-Hodgkin's B-cell lymphoma	n/a	• Initial: Chemotherapy,<br>• Secondary: nil	n/a (alive)	(44)

	Case 4	66 post-menopausal	• Asymptomatic	Cervical cytology	DLBCL	n/a	• Initial: Surgery,<br>• Secondary: Chemotherapy	n/a (alive)	(44)
Gupta 1995 Saudi Arabia	Case 1	38 pre-menopausal	• Irregular prolonged menstrual bleeding (10 months)<br>• Heavy vaginal bleeding (2 days) (10 months + 2 days)	Histology from emergency total abdominal hysterectomy	Non-Hodgkin's lymphoma type B	n/a	• Initial: Chemotherapy - cyclophosphamide, vincristine, prednisone,<br>• Secondary: Radiotherapy - Cobalt 60 external whole pelvis	3 months (alive)	(45)
Holweg 1995 Germany	Case 1	48 pre-menopausal	• Inter-menstrual bleeding (12 months)	Cervical cytology	Non-Hodgkin's B-cell lymphoma	FIGO IB IEA	• Initial: Total abdominal hysterectomy and bilateral salpingo-oophorectomy,<br>• Secondary: n/a	n/a	(46)
	Case 2	35 pre-menopausal	• Heavy menstrual bleeding (2 months)	Cervical cytology	Centrocytic-centroblastic non-Hodgkin's B-cell lymphoma	FIGO IIB IEA	• Initial: Chemotherapy - COP (cyclophosphamide, vincristine, prednisone),<br>• Secondary: Radiotherapy - external whole pelvis	n/a	(46)
Plewicka 1995 Poland	Case 1	56 post-menopausal [a]	• Abdominal pain lumbosacral spine discomfort<br>• Oedema of lower extremities (3months)	Cervical biopsy	Lymphoma	n/a	• Initial: Chemotherapy - CHOP (including 3 with Adriamycin),<br>• Secondary: Chemotherapy - nitrogranulogen and vepesid and steroid therapies	12 months (alive)	(46)

Papadopoulos 1996 UK	Case 1	36 pre-menopausal [a]	• Irregular bleeding 1 episode of post-coital bleeding (1 month)<br>• Heavy vaginal bleeding and night sweats (1 month)	Ultrasound pelvis	High grade lymphoma	n/a	• Initial: Embolisation of branches of the right uterine artery and branch vessels of the anterior division of the right internal iliac artery and distal branches of the left uterine artery,<br>• Secondary: Chemotherapy - cyclophosphamide, etoposide, adriamycin, cytosine arabinoside, bleomycin, vincristine, methotrexate	48 months (alive)	(48)
	Case 2	69 post-menopausal	• Asymptomatic	Cervical cytology	Diffuse small cleaved B-cell lymphoma	IE	• Initial: Chemotherapy,<br>• Secondary: n/a	12 months (alive)	(48)
Abbas 1996 USA	Case 1	25 pre-menopausal [a]	• Heavy vaginal bleeding	CT scan pelvis and abdomen	Non-Hodgkin's B-cell lymphoma	n/a	• Initial: Chemotherapy,<br>• Secondary: n/a	1.5 months (alive)	(49)
Dhimes 1996 Spain	Case 1	69 post-menopausal	• Asymptomatic	Cervical cytology	Diffuse small cleaved B-cell lymphoma	IE	• Initial: Total abdominal hysterectomy,<br>• Secondary: nil	12 months (alive)	(50)
Fianza 1996 Italy	Case 1	58 post-menopausal	• Menorrhagia (2 months)	Cervical biopsy	Centroblastic non-Hodgkin's B-cell lymphoma	IIE, FIGO IIb	• Initial: Chemotherapy – cyclophosphamide, cortisone<br>• Secondary: nil	132 months (alive)	(51)
	Case 2	22 pre-menopausal	• Leucorrhoea<br>• Menorrhagia	US pelvis	Large B-cell lymphoma	n/a	• Initial: Chemotherapy – CyclOBEAP<br>• Secondary: nil	12 months (alive)	(51)

al-Talib 1996 UK	Case 1	45 peri-menopausal[a]	• Post-coital bleeding	Cervical cytology	High grade Hodgkin's lymphoma	non-B-cell	n/a	• Initial: Total abdominal hysterectomy,<br>• Secondary: Chemotherapy - CHOP, Radiotherapy - pelvis	24 months (alive)	(52)
	Case 2	20 pre-menopausal[a]	• Asymptomatic	Cervical cytology	DLBCL		n/a	• Initial: Radical hysterectomy and bilateral salpingo-oophorectomy,<br>• Secondary: n/a	9 months (alive)	(52)
Biswal 1997 India	Case 1	45 pre-menopausal	• Post-coital bleeding<br>• Offensive vaginal discharge<br>• Dysuria<br>• Lower abdominal pain (6 months)	Examination under anaesthesia	DLBCL		n/a	• Initial: Chemotherapy - cyclophosphamide, doxorubicin, vincristine, prednisone,<br>• Secondary: Radiotherapy - external whole pelvis	5 months (alive)	(53)
el Ghazi 1997 Morocco	Case 1	61 post-menopausal	• Post-menopausal bleeding<br>• Vaginal discharge	Cervical biopsy	Centroblastic Hodgkin's lymphoma	non-B-cell	1E (IIIA FIGO)	• Initial: Chemotherapy - CHOP-like (cyclophosphamide, epirubicin, vincristine, prednisolone),<br>• Secondary: Radiotherapy	28 months (alive)	(54)
Lee 1998 Singapore	Case 1	67 post-menopausal[a]	• Post-coital bleeding	Polypectomy	Non-Hodgkin's lymphoma	B-cell	1EA	• Initial: Chemotherapy - CHOP,<br>• Secondary: Radiotherapy, Chemotherapy - CHOP	n/a (alive)	(55)

	Case 2	65 post-menopausal [a]	• Vaginal bleeding with clots (2 months)	Cervical biopsy	Non-Hodgkin's lymphoma	B-cell	1EA	• Initial: Chemotherapy - CHOP chemotherapy (cyclophosphamide, doxorubicin, vincristine and prednisolone),<br>• Secondary: nil	n/a (alive)	(55)
Chandy 1998 India	Case 1	50 post-menopausal	• Post-menopausal vaginal bleeding	Vaginal biopsy	Extranodal Hodgkin's lymphoma	non-B-cell	IE	• Initial: Total hysterectomy and bilateral salpingo-oophorectomy with resection of right common and external iliac nodes,<br>• Secondary: Radiotherapy	n/a (alive)	(56)
Clarke 1998 UK	Case 1	28 pre-menopausal [a]	• Post-coital bleeding (3 months)	Cystoscopy	High grade Hodgkin's lymphoma	non-B-cell	IEA	• Initial: Total hysterectomy and bilateral salpingo-oophorectomy with paraaortic node sampling and pelvic lymphadenectomy,<br>• Secondary: Radiotherapy	6 months (alive)	(57)
Kaito 1998 Japan	Case 1	28 pre-menopausal	• Irregular bleeding (2 months)	MRI pelvis	DLBCL		n/a	• Initial: Chemotherapy – CHOP<br>• Secondary: Radiotherapy	11 months (alive)	(58)
Bilgin 1999 Turkey	Case 1	74 post-menopausal	• Post-menopausal vaginal bleeding (7 months)	Cervical biopsy	Non-Hodgkin's lymphoma	B-cell	IIIE	• Initial: Chemotherapy - CHOP,<br>• Secondary: nil	24 months (alive)	(59)
Grace 1999 Ireland	Case 1	52	• Asymptomatic	Colposcopy	Follicular Hodgkin's lymphoma	non-B-cell	IIIE	• Initial: nil,<br>• Secondary: nil	4 months (alive)	(60)

		peri-menopausal [a]							
	Case 2	36 pre-menopausal [a]	• Intermenstrual bleeding<br>• Post-coital bleeding (6 months)	Colposcopy	High grade non-Hodgkin's B-cell lymphoma	IAE	• Initial: Chemotherapy - CHOP (vinblastine),<br>• Secondary: nil	n/a (alive)	(60)
Wang 1999 Taiwan	Case 1	35 pre-menopausal [a]	• Asymptomatic	Radical abdominal hysterectomy	Low grade non-Hodgkin's B-cell lymphoma	n/a	• Initial: n/a,<br>• Secondary: n/a	n/a	(61)
Isosaka 1999 Japan	Case 1	67 post-menopausal [a]	• Heavy vaginal bleeding (4 days)	CT pelvis	DLBCL	IE	• Initial: Chemotherapy – R-CHOP<br>• Secondary: Radiotherapy	24 months (alive)	(62)
Mhawech 2000 USA	Case 1	30 pre-menopausal [a]	• Lower abdominal pain<br>• Vaginal bleeding	CT scan pelvis and abdomen	NK-cell lymphoma	IE	• Initial: Radiotherapy - whole pelvis,<br>• Secondary: nil	n/a	(63)
	Case 2	45 peri-menopausal [a]	• Increasing back and bone pain (4 months)	n/a	Blastoid NK-cell lymphoma/leukaemia	n/a	• Initial: Total hysterectomy and bilateral salpingo-oophorectomy,<br>• Secondary: nil	n/a	(63)
Agarossi 2000 Italy	Case 1	38 pre-menopausal [a]	• Compelling fever<br>• Weakness<br>• Weight loss<br>• Malodorous vaginal leucorrhoea<br>• Vaginal spotting	Colposcopy	Extranodal non-Hodgkin's B-cell lymphoma	IE	• Initial: Total abdominal hysterectomy,<br>• Secondary: nil	3 months (deceased)	(64)
Kostopoulos 2000 Greece	Case 1	64 post-menopausal	• Lower abdominal pain<br>• Vaginal bleeding	n/a	DLBCL	IE	• Initial: Total abdominal hysterectomy and bilateral salpingo-oophorectomy,	n/a	(65)

							• Secondary: Chemotherapy - cyclophosphamide, doxorubicin, vincristine, prednisone		
Yokoyama 2000 Japan	Case 1	55 post-menopausal	• Watery vaginal discharge (1 month)	MRI pelvis	Large cleaved cell diffuse lymphoma	IIIEA	• Initial: Cervical conization,<br>• Secondary: n/a	12 months (alive)	(66)
Piura 2001 Israel	Case 1	20 pre-menopausal [a]	• Asymptomatic	Colposcopy	Lymphoma-like lesion	n/a	• Initial: Nil,<br>• Secondary: n/a	52 months (alive)	(67)
	Case 2	23 pre-menopausal [a]	• Post-coital bleeding (2 months)	Cervical cytology	Lymphoma-like lesion	n/a	• Initial: Chemotherapy - CEOP (cyclophosphamide, epirubicin, vincristine, prednisolone),<br>• Secondary: Radiotherapy - pelvis	18 months (alive)	(67)
Sharin-Mansouri 2001 USA	Case 1	34 pre-menopausal [a]	• Inter-menstrual bleeding<br>• Post-coital bleeding (8 months)	Colposcopy	Intermediate grade non-Hodgkin's B-cell lymphoma	IAE	• Initial: Chemotherapy - mitoxantrone, cyclophosphamide, vincristine, prednisolone,<br>• Secondary: Chemotherapy	48 months (alive)	(68)
Kaneko 2001 Japan	Case 1	72 post-menopausal	• Abdominal pain (1 month)	MRI pelvis	DLBCL	n/a	• Initial: Chemotherapy - CHOP,<br>• Secondary: Chemotherapy - mitoxantrone, etoposide, carboplatin,	3 months (deceased)	(69)

							prednisolone and another with dexamethasone		
	Case 2	65 post-menopausal	• Reduced urine output<br>• Fever (2 weeks)	CT abdomen pelvis	DLBCL	n/a	• Initial: Chemotherapy - CHOP ,<br>• Secondary: Chemotherapy - mitoxantrone, etoposide, carboplatin, prednisolone, and another with dexamethasone	6 months (deceased)	(69)
	Case 3	45 peri-menopausal[a]	• Asymptomatic	Cervical biopsy	DLBCL	n/a	• Initial: Hysterectomy,<br>• Secondary: Chemotherapy - CHOP	59 months (alive)	(69)
Vang 2001 USA	Case 1	30 pre-menopausal	• Abnormal vaginal bleeding	n/a	NK-cell	IE	• Initial: Radical abdominal hysterectomy, bilateral salpingo-oophorectomy and pelvic lymph node dissection,<br>• Secondary: Chemotherapy - CHOP	n/a	(70)
	Case 2	39 pre-menopausal	• Asymptomatic	n/a	DLBCL	IE	• Initial: Chemotherapy-proMECE/CytaBOM (cyclophosphamide, epirubicin, etoposide, prednisone, cytarabine, vincristine, bleomycin, methotrexate),	7 months (alive)	(70)

						• Secondary: Total abdominal hysterectomy and bilateral salpingo-oophorectomy		
Case 3	46 peri-menopausal [a]	• Adnexal mass and pain	n/a	DLBCL	IE	• Initial: Chemotherapy - cyclophosphamide, doxorubicin, vincristine, and prednisolone, • Secondary: Radiotherapy - pelvis	66 months (alive)	(70)
Case 4	53 peri-menopausal [a]	• Asymptomatic	n/a	Marginal zone B-cell lymphoma	IE	• Initial: Total abdominal hysterectomy, • Secondary: n/a	n/a	(70)
Case 5	57 post-menopausal [a]	• Abnormal uterine bleeding	n/a	DLBCL	IIE	• Initial: Chemotherapy, • Secondary: radiotherapy	120 months (alive)	(70)
Case 6	57 post-menopausal [a]	• Abnormal uterine bleeding	n/a	DLBCL	IIE	• Initial: Total abdominal hysterectomy and bilateral salpingo-oophorectomy, • Secondary: n/a	60 months (alive)	(70)
Case 7	61 post-menopausal [a]	• Abnormal uterine bleeding	n/a	Grade 1 Follicular Lymphoma	IIE	• Initial: Cervical conization, • Secondary: n/a	72 months (alive)	(70)
Case 8	65 post-menopausal	• Abnormal uterine bleeding	n/a	DLBCL	IE	• Initial: Chemotherapy, • Secondary: Radiotherapy	12 months (deceased)	(70)
Case 9	67 post-menopausal	• Asymptomatic	n/a	DLBCL	IE	• Initial: Chemotherapy,	108 months	(70)

							• Secondary: Radiotherapy	(deceased)	
	Case 10	67 post-menopausal	• Abnormal uterine bleeding	n/a	DLBCL	IE	• Initial: Total abdominal hysterectomy and bilateral salpingo-oophorectomy,<br>• Secondary: Chemotherapy, Radiotherapy	60 months (alive)	(70)
Jha 2001 Ireland	Case 1	67 post-menopausal	• Nausea<br>• Haematuria<br>• Lower abdominal pain (4 days)	Transabdominal ultrasound	DLBCL	n/a	• Initial: Chemotherapy,<br>• Secondary: n/a	66 months (alive)	(71)
Rossi 2001 Italy	Case 1	46 pre-menopausal	• Inter-menstrual bleeding	Colposcopy and cervical polypectomy	High grade large B-cell MALT-type lymphoma	IV	• Initial: Cervical conization,<br>• Secondary: Chemotherapy	36 months (alive)	(72)
Liro 2001 Poland	Case 1	58 post-menopausal	• Vaginal spotting (6months)<br>• Heavy vaginal bleeding<br>• Vaginal discharge (2 months)	Cervical biopsy	Non-Hodgkin's B-cell lymphoma type B	n/a	• Initial: Cervical conization,<br>• Secondary: Chemotherapy, Radiotherapy	n/a (alive)	(73)
Bode 2002 Finland	Case 1	46 peri-menopausal [a]	• Vaginal bleeding (2 weeks)	Transabdominal and transvaginal ultrasound	Follicular lymphoma	IE	• Initial: Embolization of both uterine arteries using polyvinyl alcohol particles,<br>• Secondary: Chemotherapy - CEOP, Radiotherapy	10 months (alive)	(74)
Marin 2002 Spain	Case 1	63 post-menopausal [a]	• Asymptomatic	Cervical cytology	B-cell lymphoblastic NHL (Non-Hodgkin Lymphoma)	n/a	• Initial: Radical hysterectomy and pelvic lymphadenectomy,	n/a	(75)

							• Secondary: Radiotherapy		
Lyman 2002 USA	Case 1	38 pre-menopausal	• Abdominal pain • Vaginal bleeding	Histology from hysterectomy	T-cell acute lymphoblastic leukaemia	n/a	• Initial: Chemotherapy, • Secondary: n/a	n/a (deceased)	(76)
el Mrabet 2002 Morocco	Case 1	66 post-menopausal	• Postmenopausal bleeding (5 months)	Cervical biopsy	Non-Hodgkin's B-cell lymphoma type B	1E (1A FIGO)	• Initial: Chemotherapy - CHOP, • Secondary: n/a	26 months (alive)	(77)
Au 2003 China	Case 1	45 peri-menopausal [a]	• Foul vaginal discharge	Cervical cytology	DLBCL	IIEB	• Initial: Chemotherapy - methotrexate, bleomycin, adriamycin, cyclophosphamide, vincristine dexamethasone, • Secondary: nil	96 months (alive)	(78)
	Case 2	50 post-menopausal	• Vaginal bleeding	Cervical cytology	Lymphoma-like lesion	n/a	• Initial: Large Loop Excision of the Transformation Zone (LLETZ), • Secondary: nil	12 months (alive)	(78)
	Case 3	35 pre-menopausal [a]	• Yellowish blood-stained vaginal discharge	Cervical cytology	Lymphoma-like lesion	n/a	• Initial: Large Loop Excision of the Transformation Zone (LLETZ), • Secondary: Cone biopsy	60 months (alive)	(78)
Baxter 2003 UK	Case 1	75 post-menopausal	• Post-menopausal vaginal bleeding	Ultrasound	Large cell follicular lymphoma	n/a	• Initial: Chemotherapy - Chlorambucil, • Secondary: nil	6 months (alive)	(79)
Gabriele 2003 Italy	Case 1	40 peri-menopausal [a]	• Heavy uterine bleeding	Cervical cytology	Extranodal large non-Hodgkin's B-cell lymphoma	n/a	• Initial: Chemotherapy - CHOP, • Secondary: nil	27 months (alive)	(80)

Kahlifa 2003 USA	Case 1	32 pre-menopausal [a]	• Menorrhagia<br>• LLQ pain<br>• Weight loss (3 weeks)	CT pelvis	DLBCL	n/a	• Initial: Chemotherapy - CHOP,<br>• Secondary: Radiotherapy - pelvis	10 months (alive)	(81)
Szantho 2003 USA	Case 1	56 post-menopausal	• Bloody discharge	Ultrasound	Non-Hodgkin's B-cell lymphoma	IE	• Initial: Chemotherapy - CHOP (cyclophosphamide, doxorubicin, vincristine, prednisolone),<br>• Secondary: n/a	60 months (alive)	(82)
Quattrini 2003 Italy	Case 1	57 post-menopausal [a]	• Vaginal bleeding	Colposcopy and cervical biopsy	B-cell chronic lymphocytic leukaemia/small lymphocytic lymphoma (B-CLL/SLL)	n/a	• Initial: n/a,<br>• Secondary: n/a	n/a (deceased)	(83)
Sun 2003 China	Case 1	72 post-menopausal	• Heavy vaginal bleeding<br>• Low grade fever	CT abdomen pelvis	lymphoma	n/a	• Initial: Chemotherapy - CHOP l (cyclophosphamide, adriamycin, vincristine, prednisone),<br>• Secondary: Radical hysterectomy with bilateral salpingo-oophorectomy and lymph node resection	n/a	(84)
Thyagarajan 2004 UK	Case 1	41 peri-menopausal [a]	• Urinary symptoms and menorrhagia	Cervical biopsy	High grade non-Hodgkin's B-cell lymphoma	n/a	• Initial: Chemotherapy,<br>• Secondary: Radiotherapy	7 months (alive)	(85)
KendrickJEth 2005 USA	Case 1	66 post-menopausal [a]	• Abdominal bloating<br>• Early satiety (1 month)	CT pelvis	Intermediate grade non-Hodgkin's B-cell lymphoma	IV	• Initial: n/a, Secondary: n/a	18 months (alive)	(86)

	Case 2	47 peri-menopausal[a]	• Malodorous vaginal discharge	Cervical biopsy	Intermediate grade non-Hodgkin's B-cell lymphoma	n/a	• Initial: Bilateral pelvic lymphadenectomy,<br>• Secondary: nil	16 months (alive)	(86)
Chan 2005 USA	Case 1	62 post-menopausal	• Vaginal discharge<br>• Pelvic pain	n/a	High grade small-cell non-Burkitt	IEA	• Initial: Chemotherapy - CHOP with rituxan,<br>• Secondary: nil	72 months (deceased)	(87)
	Case 2	40 pre-menopausal	• Vaginal bleeding<br>• Abdominal pain	n/a	DLBCL	IVEA	• Initial: Chemotherapy - CHOP,<br>• Secondary: nil	12 months (alive)	(87)
	Case 3	41 pre-menopausal	• Severe weakness<br>• Abdominal pain<br>• Weight loss<br>• Fever<br>• Night sweats	n/a	DLBCL	IIIEB	• Initial: Chemotherapy - CHOP with rituxan,<br>• Secondary: Total abdominal hysterectomy, chemotherapy	11 months (deceased)	(87)
	Case 4	49 pre-menopausal	• Vaginal bleeding<br>• Abdominal pain	n/a	High grade lymphoblastic lymphoma	IVEA	• Initial: Total abdominal hysterectomy and bilateral salpingo-oophorectomy and pelvic lymphadenectomy,<br>• Secondary: Radiotherapy - pelvis	36 months (alive)	(87)
	Case 5	76 post-menopausal	• Asymptomatic	n/a	DLBCL	IEA	• Initial: Chemotherapy - CHOP and rituxan,<br>• Secondary: nil	14 months (alive)	(87)
	Case 6	52 post-menopausal	• Vaginal bleeding<br>• Weight loss<br>• Fatigue	n/a	Intermediate grade predominantly large-cell lymphoma	IVEB	• Initial: Abdominal hysterectomy and bilateral salpingo-oophorectomy,	12 months (alive)	(87)

							• Secondary: Chemotherapy - CHOP		
Garavaglia 2005 Italy	Case 1	38 pre-menopausal [a]	• Asymptomatic	Cervical cytology	Non-Hodgkin's large B-cell lymphoma	IE	• Initial: Chemotherapy - CHOP,<br>• Secondary: nil	120 months (alive)	(88)
	Case 2	38 pre-menopausal [a]	• Asymptomatic	Cervical cytology	DLBCL	IIE	• Initial: Chemotherapy - MACOP-B (methotrexate, adriamycin, cyclophosphamide, vincristine, prednisone, and bleomycin),<br>• Secondary: nil	84 months (alive)	(88)
	Case 3	35 pre-menopausal [a]	• Abnormal vaginal bleeding (3 months)	Ultrasound pelvis	DLBCL	IIE	• Initial: Chemotherapy - MACOP-B (methotrexate, adriamycin, cyclophosphamide, vincristine, prednisone, and bleomycin),<br>• Secondary: Abdominal hysterectomy and bilateral salpingo-oophorectomy and pelvic lymphadenectomy	72 months (alive)	(88)
Dursun 2005 Turkey	Case 1	51 peri-menopausal [a]	• Vaginal discharge (24 months)	Radiological evaluation of cervix	Non-Hodgkin's B-cell lymphoma	IE	• Initial: Chemotherapy - CHOP,<br>• Secondary: Abdominal	19 months (alive)	(89)

							hysterectomy and bilateral salpingo-oophorectomy, Bilateral ovarian transplantation with aortic and renal lymphadenectomy		
	Case 2	49 post-menopausal	• Asymptomatic	Cervical cytology	Non-Hodgkin's B-cell lymphoma	IE	• Initial: Chemotherapy- epirubicin, cyclophosphamide, vincristine,<br>• Secondary: Radiotherapy - involved field	22 months (alive)	(89)
Alameda 2005 Spain	Case 1	37 pre-menopausal [a]	• Asymptomatic	Polypectomy	Large B-cell lymphoma-like reaction	n/a	• Initial: Chemotherapy - CHOP,<br>• Secondary: Radiotherapy - Cobalt 60 external whole pelvis	48 months (alive)	(90)
Goker 2005 Turkey	Case 1	55 post-menopausal	• Pelvic pain	n/a	Burkitt lymphoma	IE	• Initial: Chemotherapy - m-CHOP,<br>• Secondary: Radiotherapy - Cobalt 60 external whole pelvis	108 months (deceased)	(91)
Murad 2005 Pakistan	Case 1	62 post-menopausal [a]	• Irregular vaginal bleeding (1 month)	MRI pelvis	DLBCL	n/a	• Initial: Hysterectomy,<br>• Secondary: n/a	n/a	(92)
Heredia 2005 Chile	Case 1	32 pre-menopausal [a]	• Bloody vaginal discharge<br>• Post-coital bleeding (2.5 months)	Cervical biopsy	Mixed small and large B-cell non-Hodgkin's lymphoma	IEA	• Initial: Supracervical hysterectomy and bilateral salpingo-oophorectomy and omentectomy,	61 months (alive)	(93)

							• Secondary: Chemotherapy - R-CHOP		
	Case 2	31 pre-menopausal [a]	• Vaginal bloody discharge (2 months)	Colposcopy	Diffuse mixed small and large B-cell non-Hodgkin's lymphoma	IIEA	• Initial: Chemotherapy - R-CHOP,<br>• Secondary: Radiotherapy - external beam pelvis and cervix	15 months (alive)	(93)
VanRenterghem 2005 Belgium	Case 1	38 pre-menopausal	• Heavy menstrual bleeding<br>• Lower abdominal pain<br>• Lower back pain (4 months)	Transvaginal ultrasound	DLBCL	IIE Bulky disease	• Initial: Chemotherapy, Secondary: n/a	48 months (alive)	(94)
	Case 2	45 peri-menopausal [a]	• Malodorous watery vaginal discharge<br>• Vaginal spotting	Cervical polypectomy	DLBCL	IEA	• Initial: Chemotherapy - Cyclophosphamide, epirubicin, Vincristine and Prednisolone,<br>• Secondary: n/a	18 months (alive)	(94)
Huang 2005 Taiwan	Case 1	42 peri-menopausal [a]	• Vaginal spotting<br>• Abdominal pain<br>• Nausea<br>• Vomiting (1 week)	Transabdominal ultrasound	Burkitt's lymphoma	IE	• Initial: Chemotherapy - ACVBP (adriamycin, Cyclophosphamide, Vincristine, Bleomycin, Prednisolone) followed by consolidation IV methotrexate, Itostamide VP 16, Ara-C,<br>• Secondary: n/a	n/a (deceased)	(95)
Bellefqih 2006	Case 1	23	• Asymptomatic	Cervical cytology	DLBCL	n/a	• Initial: Chemotherapy,	n/a	(96)

France		pre-menopausal[a]					• Secondary: n/a		
Frey 2006 USA	Case 1	35 pre-menopausal[a]	• Menorrhagia	Cervical biopsy	DLBCL	IIE	• Initial: Chemotherapy - CHOP,<br>• Secondary: Radiotherapy - external beam cervix and pelvis	36 months (alive)	(97)
	Case 2	56 post-menopausal	• Vaginal bleeding	Transvaginal ultrasound	DLBCL	IIE	• Initial: Chemotherapy - R-CHOP,<br>• Secondary: nil	6 months (alive)	(97)
	Case 3	43 peri-menopausal[a]	• Intermenstrual bleeding	Endometrial biopsy	Marginal zone non-Hodgkin's B-cell lymphoma	IIE	• Initial: Total abdominal hysterectomy and bilateral salpingo-oophorectomy,<br>• Secondary: Chemotherapy - R-CHOP	28 months (alive)	(97)
	Case 4	49 post-menopausal	• Menorrhagia	Vaginal ultrasound	DLBCL	IIE	• Initial: Total abdominal hysterectomy and bilateral salpingo-oophorectomy and pelvic lymph nodes,<br>• Secondary: nil	n/a	(97)
González-Cejudo 2006 Spain	Case 1	26 pre-menopausal[a]	• Abnormal uterine bleeding	Ultrasound	Extranodal non-Hodgkin's B-cell lymphoma	n/a	• Initial: Total abdominal hysterectomy and bilateral salpingo-oophorectomy and pelvic lymph nodes,<br>• Secondary: Chemotherapy - R-CHOP	12 months (alive)	(98)

Gupta 2006 India	Case 1	35 pre-menopausal	• Irregular vaginal bleeding (24 months)<br>• White discharge (3 months)	CXR	Extranodal non-Hodgkin's B-cell lymphoma	IVAE	• Initial: Total abdominal hysterectomy,<br>• Secondary: Chemotherapy - R-CHOP	24 months (alive)	(99)
Semczuk 2006 Poland	Case 1	43 pre-menopausal	• n/a	Ultrasound	High grade non-Hodgkin's B-cell lymphoma	IE	• Initial: Chemotherapy - MCP-842 (cyclophosphamide, vincristine, doxorubicin, methotrexate, and cytosine arabinoside),<br>• Secondary: Chemotherapy - (mitoxantrone, cyclophosphamide, vincristine, bleomycin, VP-16)	10 months (alive)	(100)
Hariprasad 2006 India	Case 1	47 peri-menopausal [a]	• Irregular vaginal bleeding (2 months)	CT abdomen and pelvis	DLBCL	IE	• Initial: Chemotherapy - CHOP (adriamycin omitted due to cardiotoxicity),<br>• Secondary: Radiotherapy	n/a (alive)	(101)
	Case 2	80 post-menopausal	• Post-menopausal bleeding<br>• Anorexia (9 months)	CXR	DLBCL	IE	• Initial: Chemotherapy - CHOP,<br>• Secondary: nil	n/a (alive)	(101)
D cantu de Leon 2006 Mexico	Case 1	56 post-menopausal [a]	• Lower abdominal pain<br>• Arthralgia<br>• Vaginal bleeding (6 months)	Colposcopy	DLBCL	IIE	• Initial: Total laparoscopic hysterectomy and bilateral salpingo-oophorectomy,	6 months (alive)	(102)

							• Secondary: Chemotherapy - CHOP		
Paul 2006 India	Case 1	44 peri-menopausal [a]	• Heavy menstrual bleeding (6 months)<br>• Irregular menstruation<br>• Increased vaginal discharge (3 months)<br>(9 months)	Cervical cytology	DLBCL	n/a	• Initial: Total hysterectomy and bilateral salpingo-oophorectomy,<br>• Secondary: Chemotherapy - CHOP	36 months (alive)	(103)
Wannesson 2006 Canada	Case 1	46 peri-menopausal [a]	• Watery vaginal discharge<br>• Vaginal bleeding (3 weeks)	Cervical cytology	DLBCL	IEA	• Initial: Chemotherapy,<br>• Secondary: n/a	n/a (alive)	(104)
Bural 2007 USA	Case 1	35 pre-menopausal [a]	• Vaginal bleeding	CT pelvis	DLBCL	n/a	• Initial: Chemotherapy,<br>• Secondary: Total abdominal hysterectomy	n/a	(105)
Cohn 2007 USA	Case 1	64 post-menopausal [a]	• Asymptomatic	CT pelvis	DLBCL	IE	• Initial: Chemotherapy - CHOP with rituximab,<br>• Secondary: nil	13 months (alive)	(106)
	Case 2	46 peri-menopausal [a]	• Abdominal fullness<br>• Vaginal bleeding<br>• Vaginal discharge (2 months)	Abdominal and transvaginal ultrasound	DLBCL	IV	• Initial: Chemotherapy - CHOP and rituximab,<br>• Secondary: nil	24 months (deceased)	(106)
	Case 3	36 pre-menopausal [a]	• Asymptomatic	Cervical Biopsy	Follicular cutaneous nodular B-cell lymphoma	IE	• Initial: Radiotherapy,<br>• Secondary: nil	50 months (alive)	(106)

	Case 4	22 pre-menopausal [a]	• Pelvic pressure<br>• Dysmenorrhoea<br>• Abnormal vaginal bleeding	Ultrasound pelvis	DLBCL	IE	• Initial: Chemotherapy - R-CHOP,<br>• Secondary: Radiotherapy - whole pelvis	n/a	(106)
Signorelli 2007 Italy	Case 1	56 post-menopausal [a]	• n/a	Cervical biopsy	DLBCL	IIE	• Initial: Chemotherapy - CHOP ,<br>• Secondary: Radiotherapy - external beam pelvis	168 months (alive)	(107)
	Case 2	29 pre-menopausal	• n/a	Cervical biopsy	DLBCL	IIIE	• Initial: Chemotherapy - CHOP,<br>• Secondary: Cervical conisation, Chemotherapy - CHOP	130 months (alive)	(107)
	Case 3	32 pre-menopausal	• n/a	Cervical biopsy	DLBCL	IE	• Initial: Chemotherapy - ABVD,<br>• Secondary: n/a	91 months (alive)	(107)
	Case 4	45 peri-menopausal [a]	• n/a	Cervical biopsy	DLBCL	IE	• Initial: Chemotherapy - CHOP,<br>• Secondary: n/a	38 months (alive)	(107)
	Case 5	44 peri-menopausal [a]	• n/a	Cervical biopsy	DLBCL	IIE	• Initial: Chemotherapy - CHOP,<br>• Secondary: n/a	84 months (alive)	(107)
	Case 6	46 peri-menopausal [a]	• n/a	Cervical biopsy	DLBCL	IIE	• Initial: Chemotherapy - CHOP,<br>• Secondary: n/a	246 months (alive)	(107)
	Case 7	58	• n/a	Cervical biopsy	DLBCL	IE	• Initial: Chemotherapy - CHOP,	228 months (alive)	(107)

		post-menopausal [a]					• Secondary: Total abdominal hysterectomy and bilateral salpingo-oophorectomy		
	Case 8	33 pre-menopausal	• n/a	Cervical biopsy	DLBCL	IIE	• Initial: Chemotherapy - CHOP,<br>• Secondary: Total abdominal hysterectomy and bilateral salpingo-oophorectomy	227 months (alive)	(107)
	Case 9	54 peri-menopausal [a]	• n/a	Cervical biopsy	DLBCL	IE	• Initial: Chemotherapy - CHOP,<br>• Secondary: Radiotherapy - pelvis	118 months (alive)	(107)
Korcum 2007 Turkey	Case 1	67 post-menopausal	• Vaginal bleeding<br>• Back pain (2 months)	Transvaginal ultrasound	Low-grade follicular non-Hodgkins's lymphoma	IIE	• Initial: Total abdominal hysterectomy and bilateral salpingo-oophorectomy,<br>• Secondary: Chemotherapy - CHOP	n/a (alive)	(108)
Lorusso 2007 Italy	Case 1	29 pre-menopausal	• Post-coital bleeding (2 months)	Cervical biopsy	DLBCL	IEA	• Initial: Total abdominal hysterectomy and bilateral salpingo-oophorectomy,<br>• Secondary: Chemotherapy - CHOP	n/a	(109)
Mihalievic 2007 Serbia	Case 1	64 post-menopausal [a]	• Malaise<br>• Pyrexia<br>• Weight loss	Transabdominal ultrasound	Hodgkins Lymphoma nodular sclerosis type	n/a	• Initial: Total abdominal hysterectomy and	n/a (deceased)	(110)

							bilateral salpingo-oophorectomy,<br>• Secondary: n/a		
Coon<br>2008<br>UK	Case 1	56<br>post-menopausal[a]	• Vaginal spotting	Endometrial and cervical biopsy	Extranodal marginal zone B-cell lymphoma of mucosa	n/a	• Initial: Chemotherapy - CHOP,<br>• Secondary: n/a	28 months (alive)	(111)
AbHamid<br>2008<br>Malaysia	Case 1	43<br>pre-menopausal	• Prolonged menses (3 months)<br>• Post-coital bleeding (1 month)<br>(4 months)	Cervical biopsy	DLBCL	IE	• Initial: Total abdominal hysterectomy with bilateral salpingo-oophorectomy,<br>• Secondary: Radiotherapy, Chemotherapy - rituximab	n/a (alive)	(112)
Okudaira<br>2008<br>Japan	Case 1	68<br>post-menopausal	• Abnormal vaginal bleeding	Colposcopy	DLBCL	IIE	• Initial: n/a,<br>• Secondary: nil	60 months (alive)	(113)
Hanprasertpong<br>2008<br>Thailand	Case 1	25<br>pre-menopausal[a]	• Post-coital bleeding<br>• Increasing vaginal discharge (2 weeks)	Cervical biopsy	DLBCL	IE	• Initial: Chemotherapy - CHOP (cyclophosphamide, adriamycin, oncovin, and prednisone),<br>• Secondary: Radiotherapy - pelvis	36 months (alive)	(114)
Su<br>2008<br>Taiwan	Case 1	69<br>post-menopausal	• Post-menopausal bleeding<br>(1 month)	Abdominal ultrasound	DLBCL	IV (post-operative stage)	• Initial: nil,<br>• Secondary: nil	36 months (alive)	(115)
Kohler<br>2008<br>Brazil	Case 1	30<br>pre-menopausal	• Asymptomatic	Cervical biopsy	Non-Hodgkin's B-cell lymphoma type B	1E	• Initial: Chemotherapy - R-CHOP,<br>• Secondary: Radiotherapy	30 months (alive	(116)
	Case 2	57<br>post-menopausal	• Foul smelling vaginal discharge (12 months)	Cervical biopsy	Non-Hodgkin's B-cell lymphoma type B	1E	• Initial: Total abdominal hysterectomy and	18 months (alive)	(116)

							bilateral salpingo-oophorectomy,<br>• Secondary: Chemotherapy - R-CVP (rituximab, cyclophosphamide, vincristine, prednisolone)		
Demuynck 2009 France	Case 1	26 pre-menopausal [a]	• Menorrhagia (3 months)	Transparietal ultrasound pelvis	BCL1-cell lymphoma	IVE	• Initial: Chemotherapy - RCVP,<br>• Secondary: Radiotherapy - cervix	18 months (alive)	(117)
Hanley 2009 USA	Case 1	52 peri-menopausal [a]	• Abnormal vaginal bleeding	Ultrasound	DLBCL	IV	• Initial: Chemotherapy - CHOP-R,<br>• Secondary: Radiotherapy - pelvis	n/a (alive)	(118)
	Case 2	88 post-menopausal [a]	• Asymptomatic	Cervical cytology	Non-Hodgkin's B-cell lymphoma	n/a	• Initial: Chemotherapy - CHOP,<br>• Secondary: n/a	n/a (alive)	(118)
Baijal 2009 India	Case 1	44 pre-menopausal	• Vaginal bleeding<br>• Foul smelling discharge (2 months)	Cervical biopsy	DLBCL	IE	• Initial: Chemotherapy - vincristine, doxorubicin, cyclophosphamide,<br>• Secondary: Total abdominal hysterectomy and bilateral salpingo-oophorectomy	15 months (alive)	(119)
Amna 2009 UK	Case 1	46 peri-menopausal [a]	• Heavy inter-menstrual bleeding<br>• Post-coital bleeding	Asymptomatic	Follicular non-Hodgkins's lymphoma	n/a	• Initial: Radiotherapy,<br>• Secondary: n/a	12 months (alive)	(120)

Liu 2009 China	Case 1	35 pre-menopausal[a]	• n/a	n/a	DLBCL	n/a	• Initial: Surgery,<br>• Secondary: n/a	18 months (alive)	(121)
	Case 2	36 pre-menopausal[a]	• n/a	n/a	DLBCL	n/a	• Initial: Surgery,<br>• Secondary: Chemotherapy	17 months (alive)	(121)
	Case 3	37 pre-menopausal[a]	• n/a	n/a	DLBCL	n/a	• Initial: Surgery,<br>• Secondary: Chemotherapy - CHOP	12 months (alive)	(121)
Naki 2010 Turkey	Case 1	82 post-menopausal	• Vaginal discharge (12 months)	Cervical cytology	DLBCL	IE	• Initial: Total abdominal hysterectomy and bilateral salpingo-oophorectomy,<br>• Secondary: Chemotherapy - R-CHOP	n/a (alive)	(122)
Ustaalioglu 2010 Turkey	Case 1	65 post-menopausal	• Vaginal bleeding<br>• Fever<br>• Weight loss (4 months)	Abdominal and transvaginal ultrasound	DLBCL	IEB	• Initial: Chemotherapy - R-CHOP,<br>• Secondary: Radiotherapy - external whole pelvis	10 months (alive)	(123)
Novotny 2011 USA	Case 1	82 post-menopausal	• Fever<br>• Altered mental status<br>• Abdominal pain (3 weeks)	CT pelvis	DLBCL	n/a	• Initial: Chemotherapy - CHOP,<br>• Secondary: n/a	0.5 months (deceased)	(124)
Upanal 2011 Australia	Case 1	49 peri-menopausal[a]	• Abdominal fullness<br>• Change to bowel habit<br>• Vaginal discharge (3.5 months)	Transvaginal ultrasound	DLBCL	IEA	• Initial: Chemotherapy - R-CHOP,<br>• Secondary: n/a	20 months (alive)	(125)

	Case 2	51 peri-menopausal [a]	• Abdominal pain<br>• Dyspareunia<br>• Difficulty with urination<br>(2 months)	Transabdominal ultrasound	DLBCL	IIEA	• Initial: n/a,<br>• Secondary: n/a	19 months (alive)	(125)
Venizelos 2011 Greece	Case 1	33 pre-menopausal	• Vaginal bleeding (2 months)	Cervical biopsy	DLBCL	n/a	• Initial: n/a,<br>• Secondary: n/a	60 months (alive)	(126)
Dyer 2011 France	Case 1	27 pre-menopausal	• Asymptomatic	Cervical biopsy	DLBCL	n/a	• Initial: n/a,<br>• Secondary: n/a	108 months (alive)	(127)
	Case 2	23 pre-menopausal	• Post-coital bleeding<br>(6 months)	Cervical biopsy	DLBCL	n/a	• Initial: nil,<br>• Secondary: nil	60 months (alive)	(127)
Valizadeh 2011 Iran	Case 1	45 peri-menopausal [a]	• Change to vaginal discharge<br>(12 months)<br>• Blood-stained vaginal discharge (3 months)<br>(15 months)	Transabdominal ultrasound	B-cell lymphoproliferative disorder	n/a	• Initial: Chemotherapy - R-CHOP,<br>• Secondary: Radiotherapy - pelvis	12 months (alive)	(128)
Kim 2011 South Korea	Case 1	57 post-menopausal [a]	• Weight loss 6kg<br>(12 months)	Cervical cytology	DLBCL	n/a	• Initial: Chemotherapy - R-CHOP,<br>• Secondary: Radiotherapy - pelvis	n/a	(129)
	Case 2	53 peri-menopausal [a]	• Uncomfortable feeling in the abdomen	Cervical cytology	DLBCL	n/a	• Initial: Chemotherapy - CHOP,<br>• Secondary: n/a	n/a	(129)
	Case 3	66 post-menopausal [a]	• Vaginal bleeding and discharge	Ultrasound pelvis	DLBCL	n/a	• Initial: Chemotherapy - CHOP,<br>• Secondary: n/a	n/a	(129)
Chraiet 2012 Tunisia	Case 1	62 post-menopausal [a]	• Postmenopausal bleeding<br>• Lower abdominal bleeding	Ultrasound pelvis	DLBCL	IV	• Initial: Chemotherapy - R-CHOP,<br>• Secondary: nil	12 months (alive)	(130)

			(1 month)						
	Case 2	38 pre-menopausal [a]	• Menorrhagia<br>• Vaginal discharge	Cervical cytology	DLBCL	IE	• Initial: Chemotherapy - R-CHOP,<br>• Secondary: n/a	120 months (alive)	(130)
	Case 3	34 pre-menopausal [a]	• Irregular vaginal bleeding<br>• pelvic pain<br>(3 months)	MRI pelvis	DLBCL	IE	• Initial: Total abdominal hysterectomy and bilateral salpingo-oophorectomy,<br>• Secondary: Chemotherapy - CHOP	12 months (alive)	(130)
Park 2012 Korea	Case 1	53 post-menopausal	• Asymptomatic	Transvaginal ultrasound	High grade non-Hodgkin's B-cell lymphoma	IV	• Initial: Chemotherapy - CHOP,<br>• Secondary: Radiotherapy - external beam pelvis	n/a	(131)
Binesh 2012 Iran	Case 1	85 post-menopausal	• Postmenopausal vaginal bleeding (1 month)	Cervical biopsy	DLBCL	IE	• Initial: Chemotherapy - R-ACVBP (rituximab, doxorubicin, cyclophosphamide, vindesine, bleomycin, and prednisone),<br>• Secondary: nil	5 months (deceased)	(132)
Calli 2012 Turkey	Case 1	65 post-menopausal	• Urinary incontinence<br>• Menometrorrhagia<br>(1 month)	Cervical cytology	DLBCL	n/a	• Initial: Radiotherapy,<br>• Secondary: Radiotherapy - field box technique	n/a (alive)	(133)
Ramalingam 2012 USA	Case 1	25 pre-menopausal [a]	• n/a	Loop electrosurgical excision procedure (LEEP)	Lymphoma-like lesion	n/a	• Initial: Chemotherapy - cyclophosphamide, vinblastine, prednisolone,	7 months (alive)	(134)

							• Secondary: n/a		
	Case 2	19 pre-menopausal [a]	• n/a	Loop electrosurgical excision procedure (LEEP)	Lymphoma-like lesion	n/a	• Initial: Chemotherapy - R-CHOP, • Secondary: Chemotherapy - high dose methotrexate, cytarabine, Radiotherapy	n/a	(134)
	Case 3	24 pre-menopausal [a]	• n/a	Loop electrosurgical excision procedure (LEEP)	Lymphoma-like lesion	n/a	• Initial: n/a, • Secondary: n/a	21 months (alive)	(134)
	Case 4	27 post-menopausal [a]	• n/a	Loop electrosurgical excision procedure (LEEP)	Lymphoma-like lesion	n/a	• Initial: Chemotherapy - R-CHOP, • Secondary: Radiotherapy	n/a (alive)	(134)
	Case 5	25 pre-menopausal [a]	• n/a	Loop electrosurgical excision procedure (LEEP)	Lymphoma-like lesion	n/a	• Initial: n/a, • Secondary: n/a	6 months (alive)	(134)
	Case 6	19 pre-menopausal [a]	• n/a	Loop electrosurgical excision procedure (LEEP)	Lymphoma-like lesion	n/a	• Initial: n/a, • Secondary: n/a	n/a	(134)
Jastaniyah 2012 Canada	Case 1	54 peri-menopausal [a]	• Persistent vaginal bleeding	Endocervical curettage and biopsy	Nodular lymphocyte predominant Hodgkin lymphoma	IIEA	• Initial: n/a, • Secondary: n/a	18 months (alive)	(135)
Kanaan 2012 Brazil	Case 1	80 post-menopausal	• Cough • Nausea • Vomiting • Headache • Appetite loss	Ultrasound	DLBCL	IIIA	• Initial: n/a, • Secondary: n/a	n/a (deceased)	(136)

Ledwich 2012 USA	Case 1	37 pre-menopausal	• Vaginal bleeding	Transvaginal ultrasound	DLBCL	n/a	• Initial: n/a, • Secondary: n/a	26 months (deceased)	(137)
Parnis 2012 Malta	Case 1	54 post-menopausal	• Postmenopausal bleeding (3 months)	Cervical biopsy	DLBCL	IE	• Initial: n/a, • Secondary: n/a	17 months (alive)	(138)
Yalta 2012 Turkey	Case 1	56 post-menopausal [a]	• Asymptomatic	Cervical cytology	DLBCL	n/a	• Initial: Total abdominal hysterectomy and bilateral salpingo-oophorectomy and pelvic lymph node dissection, • Secondary: n/a	n/a	(139)
Yin 2012 China	Case 1	17 pre-menopausal	• Abdominal pain • Nausea and vomiting (9 months)	CT abdomen pelvis	ALK-positive Large B-cell lymphoma	III	• Initial: Chemotherapy - E-CHOP (cyclophosphamide, doxorubicin, vincristine, prednisolone, etoposide), • Secondary: Chemotherapy	4 months (alive)	(140)
Bull 2013 UK	Case 1	47 peri-menopausal [a]	• Malodorous discharge brown colour (3 weeks) • Lower abdominal pain (1 week)	Ultrasound	DLBCL	IIEB	• Initial: Total abdominal hysterectomy and bilateral salpingo-oophorectomy, • Secondary: n/a	n/a	(141)
Anagnostopoulos 2013 UK	Case 1	65 post-menopausal	• Asymptomatic	Transvaginal ultrasound	Low grade follicular lymphoma	IE	• Initial: Chemotherapy - R-CHOP, • Secondary: nil	15 months (alive)	(142)
Groszmann 2013 USA	Case 1	25 pre-menopausal	• Vaginal spotting	Transvaginal ultrasound	Large B- cell non-Hodgkins's lymphoma	n/a	• Initial: Chemotherapy, • Secondary: n/a	12 months (alive)	(143)

			• Post-coital bleeding (>2 months)						
Mouhajir 2013 India	Case 1	49 peri-menopausal [a]	• Heavy menstrual bleeding<br>• Post-coital bleeding (8 months)	Cervical biopsy	DLBCL	IEA	• Initial: Chemotherapy - CHOP,<br>• Secondary: Radiotherapy - external beam pelvis	192 months (alive)	(144)
Santos 2013 Brazil	Case 1	41 peri-menopausal [a]	• Vaginal bleeding	Cervical cytology	DLBCL	n/a	• Initial: Hysterectomy,<br>• Secondary: nil	n/a	(145)
Cao 2014 China	Case 1	20 pre-menopausal [a]	• n/a	n/a	DLBCL	IEA	• Initial: n/a,<br>• Secondary: n/a	84 months (alive)	(146)
	Case 2	58 post-menopausal [a]	• n/a	n/a	DLBCL	IEA+X	• Initial: Chemotherapy - CHOP,<br>• Secondary: nil	56 months (alive)	(146)
Bellevicine 2014 Italy	Case 1	79 post-menopausal [a]	• Abnormal vaginal bleeding	Colposcopy	DLBCL	IIE	• Initial: Chemotherapy - CHOP+RT,<br>• Secondary: nil	n/a	(147)
DeGreve 2014 Belgium	Case 1	75 post-menopausal	• Post-menopausal bleeding<br>• Right groin pain (2 weeks)	Cervical cytology	High grade diffuse large cell B-cell non-Hodgkin's lymphoma	IE	• Initial: Chemotherapy - R-CHOP,<br>• Secondary: n/a	20 months (alive)	(148)
Igwe 2014 USA	Case 1	22 pre-menopausal	• Left lower extremity oedema<br>• Pelvic pain<br>• Prolonged menses (2 months)	CT abdomen pelvis	DLBCL	IIE	• Initial: Chemotherapy - R-CHOP',<br>• Secondary: nil	n/a (alive)	(149)
Korivi 2014 USA	Case 1	43	• Asymptomatic	CT abdomen pelvis	Blastic B-cell lymphoma	n/a	• Initial: Chemotherapy - EPOCH (etoposide,	n/a (alive)	(150)

		peri-menopausal [a]					prednisone, vincristine, cyclophosphamide, and doxorubicin) and immunotherapy ofatumumab, dose reductions of bortezomib and vincristine,<br>• Secondary: Radiotherapy - intensity-modulated radiation therapy (IMRT) to cervix		
Mandato 2014 Italy	Case 1	44 peri-menopausal [a]	• Abnormal vaginal bleeding (1 month)	Hysteroscopy and endometrial biopsy	Diffuse large B-cell extranodal lymphoma	IVEA	• Initial: Vaginal myomectomy,<br>• Secondary: Chemotherapy - R-CHOP	24 months (alive)	(151)
Rubido 2014 Spain	Case 1	28 Pre-menopausal	• Dyspareunia<br>• Vaginal bleeding (6 months)	Cervical biopsy	DLBCL	n/a	• Initial: Chemotherapy – R-CHOP<br>• Secondary: Radiotherapy	120 months (alive)	(152)
Adachi 2015 Japan	Case 1	69 post-menopausal	• Lower abdominal discomfort<br>• Back pain<br>• Sudden haematuria (1 month)	Cystoscopy	DLBCL	n/a	• Initial: Chemotherapy - CHOP,<br>• Secondary: Chemotherapy - R-CHOP	36 months (alive)	(153)
Wang 2015 China	Case 1	54 peri-menopausal [a]	• Difficulty in micturition	Ultrasound	Nasal type NK/T cell lymphoma	n/a	• Initial: Total laparoscopic hysterectomy and bilateral salpingo-oophorectomy,<br>• Secondary: Nil	n/a	(154)

Pather 2015 South Africa	Case 1	44 peri-menopausal [a]	• Vaginal bleeding	n/a	Plasmablastic lymphoma	n/a	• Initial: Chemotherapy - R-CHOP,<br>• Secondary: n/a	2 months (deceased)	(155)
	Case 2	35 pre-menopausal	• Vaginal bleeding	n/a	DLBCL	n/a	• Initial: Chemotherapy - R-CHOP,<br>• Secondary: n/a	n/a (alive)	(155)
	Case 3	69 post-menopausal	• Blood-stained vaginal discharge	n/a	ALK- positive large B-cell lymphoma	n/a	• Initial: Chemotherapy - COP,<br>• Secondary: n/a	n/a (deceased)	(155)
	Case 4	34 pre-menopausal	• Vaginal bleeding	n/a	Plasmablastic lymphoma	n/a	• Initial: Chemotherapy - CHOP,<br>• Secondary: n/a	0.5 months (deceased)	(155)
	Case 5	32 pre-menopausal	• Incidental	n/a	DLBCL	n/a	• Initial: Chemotherapy - CHOP,<br>• Secondary: n/a	n/a	(155)
	Case 6	36 pre-menopausal	• Asymptomatic	n/a	Plasmablastic lymphoma	n/a	• Initial: Chemotherapy - COP,<br>• Secondary: n/a	n/a	(155)
Agarwal 2015 India	Case 1	55 post-menopausal	• Vaginal mass (6 months)<br>• Abdominal pain (4 months)<br>• Post-menopausal bleeding (20 days)<br>• Dysuria (11 months)	Transabdominal ultrasound	B-cell lymphoma	n/a	• Initial: n/a,<br>• Secondary: n/a	18 months (alive)	(156)
Kasai 2015 Japan	Case 1	67 post-menopausal	• Lower abdominal pain	MRI pelvis	DLBCL	IV	• Initial: n/a,<br>• Secondary: n/a	46 months (alive)	(157)
	Case 2	77	• Lower abdominal pain	MRI pelvis	DLBCL	IV	• Initial: Total abdominal	9 months (alive)	(157)

		post-menopausal					hysterectomy and bilateral salpingo-oophorectomy,<br>• Secondary: nil		
Bilimoria 2016 USA	Case 1	69 post-menopausal	• Postmenopausal vaginal bleeding	Ultrasound pelvis	DLBCL in a background of follicular lymphoma	n/a	• Initial: Chemotherapy - R-CHOP,<br>• Secondary: nil	n/a	(158)
Dobrosavljevic 2016 Serbia	Case 1	37 pre-menopausal [a]	• Inter-menstrual bleeding<br>• Post-coital bleeding (2 months)	Cervical Cytology	Follicular low grade non-Hodgkin's B-cell lymphoma	IEA	• Initial: Hysterectomy with bilateral salpingo-oophorectomy,<br>• Secondary: nil	12 months (alive)	(159)
Okamura 2016 USA	Case 1	26 pre-menopausal [a]	• Asymptomatic	Cervical Cytology	Atypical lymphoid hyperplasia	n/a	• Initial: Total hysterectomy,<br>• Secondary: Chemotherapy - R-CHOP	6 months (alive)	(160)
Fratoni 2016 Italy	Case 1	39 pre-menopausal [a]	• Vaginal bleeding	Colposcopy	DLBCL with a prominent spindle cell component (sarcomatoid variant)	1EA	• Initial: Large Loop Excision of the Transformation Zone (LLETZ),<br>• Secondary: Chemotherapy - chlorambucil and immunotherapy with Obinutuzumab	48 months (alive)	(161)
Hilal 2016 Germany	Case 1	73 post-menopausal [a]	• Asymptomatic	Colposcopy	Extranodal B-Chronic lymphocytic leukaemia	n/a	• Initial: n/a, Secondary: n/a	n/a	(162)
Omori 2016 Japan	Case 1	65 post-menopausal [a]	• Vaginal bleeding (1 month)	Cervical biopsy	Extranodal nasal type NK/T cell lymphoma	n/a	• Initial: n/a,<br>• Secondary: Chemotherapy - dexamethasone, cytarabine, methotrexate	4 months (deceased)	(163)

Kabaca 2016 Turkey	Case 1	49 peri-menopausal[a]	• Asymptomatic	Cervical biopsy	lymphoma	n/a	• Initial: n/a,<br>• Secondary: n/a	n/a	(164)
Sharma 2016 India	Case 1	61 post-menopausal	• Post-menopausal bleeding<br>• Weight loss 6kg (3 months)	CT chest abdomen pelvis	DLBCL	IVBEX	• Initial: Chemotherapy - CHOP and etoposide,<br>• Secondary: nil	n/a	(165)
Sun 2016 China	Case 1	63 post-menopausal	• Post-menopausal bleeding	MRI pelvis	DLBCL	n/a	• Initial: Chemotherapy - R-CHOP,<br>• Secondary: n/a	n/a	(166)
Vijayakumar 2016 USA	Case 1	67 post-menopausal	• Pelvic pain	Transvaginal ultrasound	Marginal zone B-cell lymphoma or lymphoplasmacytic lymphoma	n/a	• Initial: Chemotherapy - R-CHOP, intrathecal methotrexate,<br>• Secondary: Radiotherapy - involved field cervix	28 months (alive)	(167)
	Case 2	51 pre-menopausal	• Pelvic pain and pressure	Transvaginal ultrasound	DLBCL and Burkitt Lymphoma	n/a	• Initial: Chemotherapy - R-CHOP,<br>• Secondary: Radiotherapy - involved field cervix	20 months (deceased)	(167)
	Case 3	53 peri-menopausal[a]	• Malaise<br>• Decreased appetite<br>• Weight loss<br>• Right sided pelvic pain (1 month)	CT abdomen pelvis	DLBCL - germinal centre subtype	IE	• Initial: Chemotherapy - R-CHOP,<br>• Secondary: Radiotherapy - involved field cervix	n/a (alive)	(167)
	Case 4	44 peri-menopausal[a]	• Pelvic pain<br>• Left leg pain<br>• Weight loss (5 months)	Transvaginal ultrasound	DLBCL	n/a	• Initial: Surgery,<br>• Secondary: Chemotherapy	n/a	(167)

	Case 5	43 peri-menopausal [a]	• Heavy menstrual bleeding	Transvaginal ultrasound	(DLBCL)	n/a	• Initial: n/a,<br>• Secondary: n/a	6 months (alive)	(167)
Yang 2016 USA	Case 1	69 post-menopausal	• Irritative urinary voiding<br>• Microhaematuria	Transvaginal ultrasound	High grade B-cell lymphoma	n/a	• Initial: Total abdominal hysterectomy and bilateral salpingo-oophorectomy and pelvic lymph node dissection,<br>• Secondary: Chemotherapy - rituximab, bendamustine	n/a	(168)
Regalo 2016 Portugal	Case 1	40 pre-menopausal	• Right lower extremity pain and swelling	Imaging- does not define type	Large B-cell lymphoma non- Hodgkin's lymphoma	IIE	• Initial: Surgery,<br>• Secondary: Chemotherapy - R-CHOP, Radiotherapy	48 months (alive)	(169)
Singh 2016 India	Case 1	34 pre-menopausal	• Abdominal pain (3 months)	CT abdomen pelvis	DLBCL	n/a	• Initial: Laparoscopic bilateral salpingo-oophorectomy,<br>• Secondary: Chemotherapy - R-CHOP, Radiotherapy	60 months (alive)	(170)
	Case 2	77 post-menopausal	• Fever<br>• Abdominal pain	CT Abdomen pelvis with contrast	DLBCL	n/a	• Initial: Chemotherapy - R-CHOP,<br>• Secondary: n/a	36 months (alive)	(170)
Srivastava 2016 India	Case 1	27 pre-menopausal	• Severe abdominal pain and swelling (20 days)	Ultrasound	DLBCL	n/a	• Initial: Chemotherapy - R-CHOP,<br>• Secondary: n/a	4 months (alive)	(171)
Liu 2016 USA	Case 1	47 peri-menopausal [a]	• Heavy menstrual bleeding	Large loop excision of transformation zone	DLBCL and follicular lymphoma	IIIB	• Initial: Chemotherapy - R-CHOP,<br>• Secondary: n/a	n/a	(172)

Zhou 2016 China	Case 1	31 pre-menopausal	• Menolipsis (8 weeks)	Transvaginal ultrasound	DLBCL	n/a	• Initial: Chemotherapy - R-CHOP, • Secondary: n/a	n/a (alive)	(173)
Kosari 2017 Iran	Case 1	49 peri-menopausal [a]	• Vaginal bleeding (2 months)	CT pelvis	Peripheral T-cell lymphoma non-Hodgkin's lymphoma NOS type	n/a	• Initial: Chemotherapy - gemcitabine, dexamethasone, cisplatin (GDP), • Secondary: Chemotherapy - etoposide, ifosfamide, cisplatin, Radiotherapy	5 months (deceased)	(174)
Yang 2017 USA	Case 1	69 post-menopausal [a]	• Irritative urinary voiding • Microhaematuria	Urine cytology	High grade B-cell lymphoma	n/a	• Initial: Chemotherapy - R-CHOP rituximab, cyclophosphamide, doxorubicin, vincristine, prednisone, • Secondary: n/a	n/a	(175)
Azarhoush 2017 Iran	Case 1	43 peri-menopausal [a]	• Vaginal bleeding • vaginal discharge	Ultrasound	High grade B-cell lymphoma	n/a	• Initial: Chemotherapy - R-CHOP, • Secondary: Chemotherapy - R-CHOP	24 months (alive)	(176)
Chen 2017 China	Case 1	36 pre-menopausal	• Irregular vaginal bleeding (4 months)	CT pelvis	Extranasal natural killer (NK)/T-cell lymphoma	IIB	• Initial: Chemotherapy - CHOP, • Secondary: Radiotherapy	15 months (deceased)	(177)
Cubo 2017 Spain	Case 1	51 post-menopausal	• Post-menopausal bleeding (1 month)	Cervical biopsy	DLBCL	IE	• Initial: Chemotherapy - R-CHOP, • Secondary: nil	24 months (alive)	(178)

Roberts 2018 USA	Case 1	55 pre-menopausal	• Daily spotting (3 months) leading to heavy vaginal bleeding (1 month) (4 months)	Transvaginal ultrasound	DLBCL	n/a	• Initial: Chemotherapy - R-CHOP, • Secondary: n/a	36 months (alive)	(179)
Koyanagi 2018 Japan	Case 1	74 post-menopausal	• Asymptomatic	Transvaginal ultrasound	DLBCL	IIEA	• Initial: Chemotherapy - R-CHOP, • Secondary: Laparoscopic hysterectomy with bilateral salpingo-oophorectomy	n/a (alive)	(180)
Seresht 2018 Iran	Case 1	31 pre-menopausal	• Abnormal uterine bleeding (6 months)	Ultrasound	Non-Hodgkins B-cell lymphoma	IE	• Initial: Chemotherapy - R-CHOP, • Secondary: n/a	18 months (alive)	(181)
Bolandi 2019 Iran	Case 1	31 pre-menopausal[a]	• Abnormal uterine bleeding	Ultrasound	Non-Hodgkin's B-cell lymphoma	n/a	• Initial: Chemotherapy - CHOP, • Secondary: nil	n/a (alive)	(182)
Gui 2019 China	Case 1	65 post-menopausal	• Post-menopausal bleeding (2 weeks)	Cervical biopsy	DLBCL	IIEA	• Initial: Chemotherapy - R-CHOP, • Secondary: Chemotherapy - R-CHOP	35 months (alive)	(183)
Heremans 2019 Belgium	Case 1	42 peri-menopausal[a]	• Irregular vaginal bleeding	Transvaginal ultrasound	High grade non-Hodgkin's lymphoma	n/a	• Initial: Chemotherapy, • Secondary: n/a	n/a	(184)
Needs 2019 USA	Case 1	38 pre-menopausal	• Heavy menstrual bleeding • Inter-menstrual bleeding (12 months)	Cervical cytology	DLBCL	n/a	• Initial: n/a, • Secondary: n/a	n/a	(185)

Wilkie 2019 USA	Case 1	15 pre-menopausal	• Heavy vaginal bleeding (6 weeks)	Transvaginal ultrasound	Burkitt Lymphoma	n/a	• Initial: COCP regimen, • Secondary: Chemotherapy	3 months (alive)	(186)
Costa 2020 UK	Case 1	54 post-menopausal	• Feeling of vaginal mass tender to touch • Fever • Chills • Night sweats • Intermittent lower left abdominal pain (2 weeks) • Weight loss (1 year)	Cervical biopsy	DLBCL	n/a	• Initial: Chemotherapy - EPOCH-R, • Secondary: Chemotherapy - R-CODOX-M with IVAC and methotrexate	4 months (alive)	(187)
Del 2020 France	Case 1	36 pre-menopausal [a]	• Vaginal bleeding • Pelvic pain • Dysuria • Asthenia (2 weeks)	Transvaginal ultrasound	DLBCL	IV	• Initial: Chemotherapy - R-CHOP (Rituximab, Cyclophosphamide, Hydroxydaunorubicin, Vincristine, and Prednisone, • Secondary: Chemotherapy - high dose methotrexate	15 months (alive)	(188)
Goda 2020 India	Case 1	52 post-menopausal	• Post-menopausal bleeding (2 months)	Histology	DLBCL	IAE	• Initial: Chemotherapy - R-CHOP, • Secondary: Radiotherapy - involved field	18 months (alive)	(189)
	Case 2	50 post-menopausal	• Post-menopausal bleeding	n/a	DLBCL	IE	• Initial: Chemotherapy - R-CHOP, • Secondary: Radiotherapy - involved field	43 months (alive)	(189)

	Case 3	39<br>pre-menopausal [a]	• Foul smelling discharge<br>(9 months)	n/a	DLBCL		• Initial: Chemotherapy - R-CHOP,<br>• Secondary: Radiotherapy - involved field	8 months (alive)	(189)
	Case 4	62<br>post-menopausal	• Post-menopausal bleeding<br>(5 months)	n/a	DLBCL		• Initial: Chemotherapy - R-CEOP,<br>• Secondary: Radiotherapy - involved field	10 months (alive)	(189)
SelviDemirtas<br>2020<br>Turkey	Case 1	46<br>peri-menopausal [a]	• Vaginal bleeding	Cervical biopsy	DLBCL	n/a	• Initial: Chemotherapy - R-CHOP,<br>• Secondary: n/a	n/a	(190)
Liu<br>2020<br>China	Case 1	74<br>post-menopausal	• Fever expectoration (2 weeks)<br>• Night sweats<br>• Low mood<br>• Weight loss 4kg (12 months) (13 months)	CT	DLBCL	IVB	• Initial: Chemotherapy - ifosfamide, carboplatin, etoposide,<br>• Secondary: Allogenic stem cell transplant	n/a (alive)	(191)
MohammedSaeed<br>2020<br>USA	Case 1	50<br>post-menopausal	• Symptomatic anaemia<br>• Abdominal pain<br>• Abnormal vaginal bleeding	Left inguinal lymph node biopsy	Nodular sclerosis classical Hodgkin lymphoma involving cervix	n/a	• Initial: Chemotherapy - R-CHOP,<br>• Secondary: n/a	12 months (deceased)	(192)
Murata<br>2020<br>Japan	Case 1	50<br>peri-menopausal [a]	• Asymptomatic	Cervical cytology	Spindle cell variant diffuse large B-cell lymphoma	IV	• Initial: Chemotherapy - R-CHOP,<br>• Secondary: n/a	6 months (alive)	(193)
	Case 2	46<br>peri-menopausal [a]	• Abnormal vaginal bleeding	Cervical cytology	Spindle cell variant diffuse large B-cell lymphoma	IV	• Initial: Chemotherapy - cyclophosphamide, adriamycin,	42 months (alive)	(193)

							vincristine, prednisone,<br>• Secondary: Radiotherapy - external beam pelvis		
Yoshida 2020 Japan	Case 1	47 peri-menopausal [a]	• Atypical vaginal bleeding	Transvaginal ultrasound	DLBCL	IIEA	• Initial: Chemotherapy - R-CHOP,<br>• Secondary: n/a	36 months (alive)	(194)
Akkour 2021 Saudi Arabia	Case 1	54 peri-menopausal [a]	• Abnormal vaginal bleeding<br>• Pelvic pain (1 month)	MRI pelvis	DLBCL	IE	• Initial: Chemotherapy - R-CHOP,<br>• Secondary: Radiotherapy	24 months (alive)	(195)
Crespo 2021 Spain	Case 1	83 post-menopausal [a]	• Vaginal bleeding	Transvaginal ultrasound	Non-Hodgkin's B-cell lymphoma	n/a	• Initial: Chemotherapy - R-CHOP,<br>• Secondary: Radiotherapy - humerus and pelvis	n/a	(196)
Desana 2021 Italy	Case 1	54 post-menopausal	• Asymptomatic	Transvaginal ultrasound	DLBCL	n/a	• Initial: Embolization of uterine arteries,<br>• Secondary: Chemotherapy	n/a	(197)
Pons 2021 Spain	Case 1	48 peri-menopausal [a]	• Bleeding during intercourse (1 month)	Cervical biopsy	DLBCL	IE	• Initial: Laparotomic bilateral hysteron salpingectomy with lymphadenectomy,<br>• Secondary: Chemotherapy - R-CHOP	n/a	(198)
Birge 2021 Turkey	Case 1	40 peri-menopausal [a]	• Post-coital bleeding<br>• Dyspareunia<br>• Watery vaginal discharge with foul odour	Cervical biopsy	High grade B-cell lymphoma	n/a	• Initial: Nil, Secondary: n/a	10 months (alive)	(199)

Kim 2021 Korea	Case 1	81 post-menopausal	• Vaginal bleeding (3 months)	Tele-cervicography	DLBCL	n/a	• Initial: Chemotherapy - R-CHOP, • Secondary: n/a	n/a	(200)
Sasaki 2021 Japan	Case 1	77 post-menopausal [a]	• Abdominal pain	CT abdomen pelvis	DLBCL	FIGO IV	• Initial: Chemotherapy – R-THP-COP • Secondary: nil	13 months (alive)	(201)
Shim 2021 South Korea	Case 1	84 post-menopausal	• Vaginal bleeding • Difficulty urinating	CT abdomen pelvis	B-cell non-Hodgkin lymphoma- double hit subtype	IVA	• Initial: Chemotherapy - R-CHOP, • Secondary: Radiotherapy	n/a (alive)	(202)
Capsa 2022 Switzerland	Case 1	75 post-menopausal [a]	• Significant vaginal bleeding	Cervical biopsy	DLBCL	IE	• Initial: Chemotherapy - R-CHOP, • Secondary: nil	29 months (alive)	(203)
Stabile 2022 Switzerland	Case 1	83 post-menopausal	• Vaginal bleeding	Transvaginal ultrasound	DLBCL	IEA	• Initial: Chemotherapy - R-CHOP, • Secondary: n/a	60 months (alive)	(204)
Saksena 2022 USA	Case 1	44 peri-menopausal [a]	• Heavy menstrual bleeding	Histology from vaginal hysterectomy	DLBCL	n/a	• Initial: Chemotherapy - R-CHOP, • Secondary: n/a	10 months (alive)	(205)
	Case 2	43 peri-menopausal [a]	• Asymptomatic	Histology from hysterectomy	B- cell lymphoma - not otherwise specified	n/a	• Initial: Chemotherapy - CHOP, • Secondary: nil	n/a (alive)	(205)
	Case 3	36 pre-menopausal	• Asymptomatic	Endocervical curettage and biopsy	DLBCL	n/a	• Initial: Chemotherapy - R-CHOP, • Secondary: n/a	58 months (alive)	(205)
	Case 4	48 peri-menopausal [a]	• Gynaecological symptoms	Histology from hysterectomy	DLBCL	n/a	• Initial: Large Loop Excision of the Transformation Zone (LLETZ),	204 months (alive)	(205)

							• Secondary: Chemotherapy - R-CHOP		
	Case 5	46 peri-menopausal [a]	• Abnormal vaginal bleeding post-hysterectomy (24 months)	Histology from trachelectomy	DLBCL	n/a	• Initial: Hysterectomy, • Secondary: n/a	93 months (alive)	(205)
	Case 6	37 pre-menopausal	• Abnormal vaginal bleeding	Cervical cone biopsy	Grade 3 Follicular diffuse large B-cell lymphoma	n/a	• Initial: n/a, • Secondary: n/a	132 months (alive)	(205)
Alameh 2022 Iran	Case 1	41 peri-menopausal [a]	• Pelvic discomfort • Abnormal vaginal discharge • Dyspareunia (3 months)	Cervical cytology	DLBCL	IE	• Initial: Loop electrosurgical procedure and rituximab, • Secondary: n/a	60 months (alive)	(206)
Aminimoghaddam 2022 Iran	Case 1	38 pre-menopausal	• Post-coital bleeding (6 months)	Cervical biopsy	DLBCL	IE	• Initial: Hysterectomy, • Secondary: n/a	n/a (alive)	(207)
	Case 2	30 pre-menopausal	• Post-coital bleeding (12 months) • Heavy vaginal discharge (1 month) (13 months)	Cervical biopsy	DLBCL	IIE	• Initial: n/a, • Secondary: n/a	12 months (alive)	(207)
Li 2022 China	Case 1	71 post-menopausal	• Dysuria (2 months)	Cervical cytology	DLBCL	IV	• Initial: Hysterectomy, • Secondary: n/a	n/a (deceased)	(208)
Rodić 2022 Serbia	Case 1	35 pre-menopausal [a]	• Asymptomatic	Endocervical curettage and biopsy	DLBCL - centroblastic germinal centre B-cell like (GCB) subtype	n/a	• Initial: Chemotherapy - cyclophosphamide, etoposide, steroids, • Secondary: n/a	4 months (alive)	(209)
Weng 2022 China	Case 1	63 post-menopausal [a]	• Abnormal vaginal discharge (20 days)	n/a	DLBCL	FIGO IA	• Initial: Complete surgical excision of the mass,	89 months (deceased)	(210)

						• Secondary: Radiotherapy		
Case 2	55 peri-menopausal [a]	• Asymptomatic (1 month)	n/a	DLBCL	FIGO IA	• Initial: Surgery, • Secondary: Radiotherapy, Chemotherapy-CHOP	76 months (alive)	(210)
Case 3	39 pre-menopausal	• Abnormal vaginal bleeding (2 months)	n/a	DLBCL	FIGO IVA	• Initial: Chemotherapy - R-CHOP, • Secondary: n/a	n/a	(210)
Case 4	26 pre-menopausal	• Abnormal vaginal bleeding (2 months)	n/a	DLBCL	FIGO IA	• Initial: Chemotherapy, • Secondary: n/a	n/a	(210)
Case 5	42 peri-menopausal [a]	• Abnormal vaginal bleeding (2 months)	n/a	DLBCL	FIGO IA	• Initial: Chemotherapy, • Secondary: n/a	156 months (alive)	(210)
Case 6	30 pre-menopausal	• Abnormal vaginal bleeding (1 month)	n/a	DLBCL	FIGO IA	• Initial: Chemotherapy - R-CHOP, • Secondary: Surgery	132 months (alive)	(210)
Case 7	56 post-menopausal [a]	• Abnormal vaginal bleeding (3 months)	n/a	DLBCL	FIGO IB	• Initial: Chemotherapy - CHOP, • Secondary: Chemotherapy - Ifosfamide, methotrexate, Surgery	n/a	(210)
Case 8	49 peri-menopausal [a]	• Abnormal vaginal bleeding (1 month)	n/a	DLBCL	FIGO IIA	• Initial: Surgery, • Secondary: Chemotherapy	4 months (deceased)	(210)
Case 9	73 post-menopausal [a]	• Abnormal vaginal bleeding (1 month)	n/a	DLBCL	FIGO IIA	• Initial: Chemotherapy, • Secondary: n/a	31 months (deceased)	(210)

	Case 10	65 post-menopausal[a]	• Abdominal pain (1 month)	n/a	DLBCL)	FIGOIV B	• Initial: Chemotherapy,<br>• Secondary: Radiotherapy	101 months (alive)	(210)
	Case 11	67 post-menopausal[a]	• Abdominal pain (12 months)	n/a	Mantle cell lymphoma - blastoid variant	FIGO IVA	• Initial: Chemotherapy - R-CHOP,<br>• Secondary: n/a	33 months (deceased)	(210)
	Case 12	22 pre-menopausal	• Abdominal pain (1 month)	n/a	DLBCL	FIGO IA	• Initial: Chemotherapy - CHOP,<br>• Secondary: Chemotherapy - etoposide, doxorubicin, cyclophosphamide, vincristine, prednisolone	45 months (alive)	(210)
	Case 13	74 post-menopausal[a]	• Abnormal vaginal bleeding (1 month)	n/a	DLBCL	FIGO IA	• Initial: Chemotherapy - CHOP,<br>• Secondary: Radiotherapy	14 months (alive)	(210)
Seal 2022 India	Case 1	47 peri-menopausal[a]	• Fever (15 days)<br>• Weakness left lower limb<br>• Right sided deviation of mouth (1 day)<br>• Abnormal vaginal bleeding (15 days)	Transabdominal ultrasound	Extranodal peripheral T-cell lymphoma	n/a	• Initial: Radiotherapy,<br>• Secondary: n/a	n/a (deceased)	(211)
Shi 2023 China	Case 1	34 pre-menopausal[a]	• Yellow leucorrhoea	Colposcopy	Atypical lymphoid hyperplasia	n/a	• Initial: Chemotherapy - R-CHOP,<br>• Secondary: nil	6 months (alive)	(212)

Gao 2023 China	Case 1	66 post-menopausal [a]	• Haematuria	n/a	DLBCL	IVE	• Initial: Chemotherapy - R-CHOP<br>• Secondary: Radiotherapy - external beam	8 months (deceased)	(213)
	Case 2	72 post-menopausal [a]	• Back pain	n/a	DLBCL	IE	• Initial: Total abdominal hysterectomy and bilateral salpingo-oophorectomy and pelvic and para-aortic lymphadenectomy,<br>• Secondary: Chemotherapy - R-EPOCH (rituximab, etoposide, prednisone, vincristine, cyclophosphamide, and hydroxydaunorubicin)	97 months (alive)	(213)
	Case 3	56 post-menopausal [a]	• Abdominal pain	n/a	DLBCL	IVE	• Initial: nil,<br>• Secondary: nil	12 months (alive)	(213)
	Case 4	62 post-menopausal [a]	• Asymptomatic	n/a	DLBCL	IVE	• Initial: Chemotherapy - R-hyperCVAD (cyclophosphamide, vincristine, adriamycin, and dexamethasone),<br>• Secondary: nil	1.5 month (deceased)	(213)
	Case 5	31	• Lower limb pain	n/a	Burkitt lymphoma	IVE	• Initial: Chemotherapy - R-MA and CVP-R,	6 months (alive)	(213)

	pre-menopausal[a]					• Secondary: nil		
Case 6	58 post-menopausal[a]	• Back pain	n/a	DLBCL	IVE	• Initial: Chemotherapy - R-CHOP (4 with Ara-C + dexamethasone intrathecal injection),<br>• Secondary: nil	27 months (alive)	(213)
Case 7	71 post-menopausal[a]	• Abnormal uterine bleeding	n/a	DLBCL	IVE	• Initial: Chemotherapy - R-CHOP (Ara-C + 4 methotrexate intrathecal injections)<br>• Secondary: nil	38 months (deceased)	(213)
Case 8	58 post-menopausal[a]	• Abnormal uterine bleeding	n/a	DLBCL	IE	• Initial: Loop electrosurgical excision procedure<br>• Secondary: n/a	88 months (alive)	(213)

[a]Studies that did not clarify menopausal status, but was inferred using the patient age; patients younger than 45 years old were defined as pre-menopausal, those aged 45-55 years old were defined as peri-menopausal, and those older than 55 years were defined as post-menopausal._CT, computerised tomography; DLBCL, diffuse large B-cell lymphoma; MRI, magnetic resonance imaging; n/a: not available. ACVBP, doxorubicin, bleomycin, vinblastine, and dacarbazine; RCVP, rituximab, cyclophosphamide, vincristine; ECHOP, cyclophosphamide, doxorubicin, vincristine, etoposide, prednisolone; GDP, gemcitabine, dexamethasone, and cisplatin; R-THP-COP, tetrahydropyranyl adriamycin, cyclophosphamide, vincristine, prednisone; CHOP, cyclophosphamide, doxorubicin, vincristine, prednisone; R-CHOP, rituximab, cyclophosphamide, doxorubicin, vincristine, prednisone; F-MACOP, 5-fluorouracil, methotrexate with leucovorin rescue, cytarabine, cyclophosphamide, vincristine, and prednisone; CyclOBEAP, cyclophosphamide, vincristine, bleomycin, etoposide, doxorubicin, prednisolone; CHOP-MTX, cyclophosphamide, doxorubicin, vincristine, prednisone, methotrexate; pro-MACE-Cyto-BOM, cyclophosphamide, doxorubicin, etoposide cytozar, bleomycin, vincristine, methotrexate and prednisone; CEOP, cyclophosphamide, etoposide, prednisolone, vincristine; COP, cyclophosphamide, vincristine, prednisone.

Table SII. Summary of characteristics of the included cases.

Variable	Value
Age (data available for 339 patients), years	
Mean	48.5
Median	46
Range	15-88
Symptoms (data available for 318 patients), n (%)	
Vaginal bleeding (all types)	189/318 (59.4)
Post-coital bleeding	33/318 (10.4)
Intermenstrual bleeding	12/318 (3.8)
Vaginal spotting	8/318 (2.5)
Vaginal discharge (all types)	44/318 (13.8)
Abdominal pain	34/318 (10.7)
Pelvic pain	12/318 (3.8)
"B" symptoms	28/318 (8.8)
Asymptomatic	42/318 (13.2)
1st diagnostic modality used (data available for 278 patients), n (%)	
Cervical biopsy	78/278 (28.1)
Ultrasound	59/278 (21.2)
Cervical cytology	56/278 (20.1)
Computerised tomography	24/278 (8.6)
Positive histological markers (data available for 136 patients), n (%)	
CD3	61/136 (44.9)
CD5	29/136 (21.3)
CD10	48/136 (35.3)
CD20	15/136 (11)
Cyclin D1	34/136 (25)
BCL-2	16/136 (11.8)
Ki-67 (>80%)	0 (0)
Staging – Ann Arbour (data available for 179 patients), n (%)	
I	93/179 (52)
II	47/179 (26.3)
III	8/179 (4.5)
IV	29/179 (16.2)
Metastasis at time of diagnosis (data available for 245 patients), n (%)	

Yes	26/245 (10.6)
No	219/245 (89.4)
Lymphoma types (data available for 339 patients)	
Non-Hodgkin’s	265/339 (78.2)
Hodgkin’s	5/339 (1.5)
B-cell	252/339 (74.3)
T-cell	8/339 (2.4)
Recurrence (data available for 225 patients)	
Yes	120/225 (53.3)
No	105/225 (46.7)
Outcome (data available for 279 patients)	
Alive	233/279 (83.2)
Deceased	46/279 (16.5)

Table SIII. Summary of therapeutic approaches utilised for the included patients (data available for 309 patients).

Approach	Rate	%
Surgery (all types, data available for 115 patients)		
Hysterectomy & lymphadenectomy	23/115	20.0
Cervical conisation	10/115	9.1
Embolization	3/115	2.6
Surgery type not specified	14/115	12.2
Types of hysterectomy (data available for 85 patients)		
Radical hysterectomy	9/85	10.6
Total hysterectomy	58/85	68.2
Unknown type of hysterectomy	18/85	21.2
Neo-adjuvant (data available for 19 patients)		
Chemotherapy	15/19	78.9
Radiotherapy	4/19	21.1
Adjuvant (data available for 62 patients)		
Chemotherapy	35/62	56.5
Radiotherapy	18/62	29.0
Chemotherapy only	109/194	56.2
Radiotherapy only	23/194	11.9
Chemotherapy & Radiotherapy	62/194	32.0

Table SIV. Management trends over time.

Decades	Surgery only	Chemotherapy only	Radiotherapy only	Chemo-radiotherapy only	Adjuvant Chemotherapy	Adjuvant radiotherapy	Adjuvant chemo-radiotherapy	Neoadjuvant chemotherapy	Neoadjuvant radiotherapy
1964-1973	0	0	1	0	0	1	0	0	1
1974-1983	0	0	2	1	0	1	0	0	1
1984-1993	6	4	11	8	4	9	0	0	2
1994-2003	11	22	5	13	7	4	6	3	0
2004-2013	8	32	3	23	14	2	0	9	0
2014-2023	9	51	1	17	10	1	3	3	0
Sum	34	109	23	62	35	18	9	15	4

Table SV. Quality assessment of included studies (case reports).

First author, year	Q1	Q2	Q3	Q4	Q5	Q6	Q7	Q8	(Refs.)
Nasiell, 1964	●	●	●	●	●	○	○	●	(1)
Moores, 1965	○	●	●	●	●	○	○	●	(2)
Anderson , 1967	●	●	●	●	●	○	○	●	(3)
Carr, 1976	○	●	●	●	●	●	●	●	(5)
Steinffeld, 1979	●	●	●	●	●	●	○	●	(7)
Tunca, 1979	●	●	●	●	●	●	●	●	(6)
Bowen, 1985	●	●	●	●	●	○	○	●	(9)
Taki, 1985	●	●	●	●	●	●	○	●	(11)
Bar, 1986	○	●	●	●	●	●	●	●	(12)
Mann, 1987	●	●	●	●	●	●	○	●	(13)
Cardillo, 1987	●	●	●	●	●	●	○	●	(14)
Strang, 1988	●	●	●	●	●	●	○	●	(16)
Ibrahim, 1988	●	●	●	●	●	●	○	●	(17)
Khong, 1989	●	●	●	●	●	●	●	●	(18)
Kurup, 1989	●	●	●	●	●	○	○	●	(20)
Cambell, 1989	●	●	●	●	●	●	○	●	(22)
Johnston, 1989	●	●	●	●	●	●	●	●	(23)
Matsuyama, 1989	○	●	●	●	●	●	○	●	(24)
Stickelmann, 1989	○	●	●	●	●	●	○	●	(26)
Mathisasen, 1990	○	●	●	●	●	●	⦸	●	(27)
Dang, 1991	○	●	●	●	○	○	◌	●	(28)
Hachisuga, 1991	●	○	●	●	●	●	○	●	(29)
Pasini, 1991	○	●	●	●	●	●	○	●	(30)
Malatskey, 1991	○	○	●	●	●	○	○	●	(31)
Rodier, 1993	○	●	●	●	●	○	⦸	●	(37)
Cuykx, 1994	○	●	●	●	●	●	○	●	(39)
Awwad, 1994	●	●	●	●	●	●	●	●	(40)
Huh, 1994	●	●	●	○	●	●	○	●	(41)
Patsner, 1994	●	●	●	●	●	●	●	●	(42)
Gupta, 1995	●	●	●	●	●	●	○	●	(45)
Plewicka, 1995	○	●	●	●	●	●	●	●	(47)
Papadopoulos, 1996	●	●	●	●	●	●	●	●	(48)
Abbas, 1996	○	●	●	●	●	●	○	●	(49)
Dhimes, 1996	●	●	●	●	●	●	○	●	(50)
Biswal, 1997	○	●	●	●	●	●	○	●	(53)
el Ghazi, 1997	●	○	●	●	●	●	⦸	●	(54)
Chandy, 1998	●	●	●	●	●	●	○	●	(56)
Clarke, 1998	○	●	●	●	●	●	○	●	(57)
Bilgin, 1999	○	●	●	●	●	●	○	●	(59)
Wang, 1999	○	○	●	○	●	○	○	●	(61)
Agarossi, 2000	●	●	●	●	●	●	○	●	(64)
Kostopoulos, 2000	○	●	●	●	○	○	○	●	(65)
Yokoyama, 2000	●	●	●	●	●	●	●	●	(66)
Sharin-Mansouri, 2001	○	●	●	●	●	●	○	●	(68)
Jha, 2001	●	●	●	●	●	●	●	●	(71)
Rossi, 2001	○	●	●	●	●	●	○	●	(72)
Liro, 2001	●	●	●	●	●	○	○	●	(73)
Bode, 2002	●	●	●	●	●	●	○	●	(74)
MarÃn, 2002	○	●	●	●	●	○	○	●	(75)
Lyman, 2002	●	●	●	●	●	●	○	●	(76)
el Mrabet, 2002	●	●	●	●	●	●	⦸	●	(77)
Baxter, 2003	○	●	●	●	●	●	○	●	(79)
Gabriele, 2003	○	○	●	○	●	●	○	●	(80)
Kahlifa, 2003	●	●	●	●	●	●	○	●	(81)
SzÃ¡nthÃ, 2003	○	●	●	●	●	●	●	●	(82)

Quattrini, 2003	○	●	●	●	●	●	●	○	(83)
Sun, 2003	○	○	●	○	○	○	○	●	(84)
Thyagarajan, 2004	○	●	●	●	●	●	○	●	(85)
Alameda, 2005	○	○	●	●	○	○	○	●	(90)
Goker, 2005	○	●	●	●	●	●	○	●	(91)
Murad, 2005	●	●	●	●	○	○	○	●	(92)
Huang, 2005	○	●	●	●	●	●	●	●	(95)
Bellefqih, 2006	○	○	●	●	○	○	○	●	(96)
González-Cejudo, 2006	○	○	●	●	●	●	○	●	(98)
Gupta, 2006	○	●	●	●	●	●	○	●	(99)
Semczuk, 2006	○	●	●	●	●	●	○	●	(100)
D cantu de Leon, 2006	●	●	●	●	●	●	○	●	(102)
Paul, 2006	●	●	●	●	●	●	○	●	(103)
Wannesson, 2006	○	●	●	●	●	○	○	●	(104)
Bural, 2007	○	○	●	●	●	●	○	○	(105)
Korcum, 2007	●	●	●	●	●	●	○	●	(108)
Lorusso, 2007	○	●	●	●	●	●	●	●	(109)
Mihalievic, 2007	○	●	●	●	●	○	○	●	(110)
Coon, 2008	○	○	●	●	●	●	○	●	(111)
AbHamid, 2008	●	●	●	●	●	●	●	●	(112)
Okudaira, 2008	○	○	●	●	●	●	○	●	(113)
Hanprasertpong, 2008	●	●	●	●	●	●	●	●	(114)
Su, 2008	●	●	●	●	●	●	○	●	(115)
Demuynck, 2009	●	●	●	●	●	●	○	●	(117)
Baijal, 2009	●	●	●	●	●	●	●	●	(119)
Amna, 2009	●	●	●	●	●	●	○	●	(120)
Naki, 2010	●	●	●	●	●	●	○	●	(122)
Ustaalioglu, 2010	○	●	●	●	●	●	●	●	(123)
Novotny, 2011	●	●	●	●	●	○	○	●	(124)
Venizelos, 2011	○	●	●	●	●	●	○	●	(126)
Valizadeh, 2011	●	●	●	●	●	●	●	●	(128)
Park, 2012	●	●	●	●	○	○	○	●	(131)
Binesh, 2012	●	●	●	●	●	●	○	●	(132)
Calli, 2012	●	●	●	●	●	●	○	●	(133)
Jastaniyah, 2012	●	●	●	●	●	●	●	●	(135)
Kanaan, 2012	○	○	●	●	●	●	●	●	(136)
Ledwich, 2012	○	●	●	●	●	●	○	●	(137)
Parnis, 2012	●	●	●	●	●	●	●	●	(138)
Yalta, 2012	○	●	●	●	○	○	○	●	(139)
Yin, 2012	●	●	●	●	○	○	○	●	(140)
Bull, 2013	●	●	●	●	●	●	○	●	(141)
Anagnostopoulos, 2013	○	●	●	●	●	●	○	●	(142)
Groszmann, 2013	○	●	●	●	●	●	○	●	(143)
Mouhajir, 2013	○	●	●	●	●	●	○	●	(144)
Santos, 2013	○	●	●	○	●	●	○	●	(145)
Bellevicine, 2014	●	○	●	●	○	○	○	●	(147)
DeGreve, 2014	●	●	●	●	●	●	○	●	(148)
Igwe, 2014	●	●	●	●	●	●	○	●	(149)
Korivi, 2014	●	●	●	●	●	○	●	●	(150)
Mandato, 2014	○	●	●	●	●	●	○	●	(151)
Adachi, 2015	○	●	●	●	●	●	○	●	(153)
Wang, 2015	○	●	●	●	●	●	○	●	(154)
Agarwal, 2015	●	●	●	●	●	●	○	●	(156)
Bilimoria, 2016	○	○	●	●	●	●	○	●	(158)
Dobrosavljevic, 2016	●	●	●	●	●	●	○	●	(159)
Okamura, 2016	○	○	●	●	○	●	○	●	(160)
Fratoni, 2016	●	●	●	○	●	○	○	●	(161)
Hilal, 2016	○	●	●	●	●	○	○	●	(162)

Omori, 2016	●	●	●	●	●	●	○	●	(163)
Kabaca, 2016	○	○	●	●	●	○	○	●	(164)
Sharma, 2016	○	●	●	●	●	○	●	●	(165)
Sun, 2016	○	○	●	●	○	○	○	○	(166)
Yang, 2016	●	●	●	●	●	○	○	●	(168)
Regalo, 2016	○	●	●	●	●	●	○	●	(169)
Srivastava, 2016	●	●	●	●	●	●	○	●	(171)
Liu, 2016	○	●	●	●	●	●	○	●	(172)
Zhou, 2016	○	●	●	●	○	○	○	●	(173)
Kosari, 2017	●	●	●	●	●	●	●	●	(174)
Yang, 2017	○	●	●	●	●	○	○	●	(175)
Azarhoush, 2017	○	○	●	●	●	●	○	●	(176)
Chen, 2017	●	●	●	●	●	●	●	●	(177)
Cubo, 2017	○	●	●	●	●	●	○	●	(178)
Roberts, 2018	○	●	●	●	●	●	○	●	(179)
Koyanagi, 2018	●	●	●	●	●	●	○	●	(180)
Seresht, 2018	○	●	●	●	●	●	○	●	(181)
Bolandi, 2019	○	●	●	○	●	●	○	●	(182)
Gui, 2019	○	●	●	●	●	●	○	●	(183)
Heremans, 2019	○	●	●	●	●	○	○	●	(184)
Needs, 2019	●	●	●	●	○	○	○	●	(185)
Wilkie, 2019	●	●	●	●	●	●	○	●	(186)
Costa, 2020	●	●	●	●	●	●	●	●	(187)
Del, 2020	●	●	●	●	●	●	○	●	(188)
SelviDemirtas, 2020	○	○	●	●	●	●	○	●	(190)
Liu, 2020	●	●	●	●	●	●	○	●	(191)
MohammedSaeed, 2020	●	●	●	●	●	●	●	●	(192)
Yoshida, 2020	●	○	●	●	●	●	●	●	(194)
Akkour, 2021	●	●	●	●	●	●	○	●	(195)
Crespo, 2021	○	○	●	●	○	○	○	●	(196)
Desana, 2021	○	●	●	●	●	○	○	●	(197)
Pons, 2021	●	●	●	●	●	○	○	●	(198)
BirgeÃ, 2021	○	●	●	●	●	●	○	●	(199)
Kim, 2021	●	●	●	●	●	○	○	●	(200)
Shim, 2021	●	●	●	●	●	●	○	●	(202)
Capsa, 2022	○	●	●	●	●	●	○	●	(203)
Stabile, 2022	●	●	●	●	●	●	○	●	(204)
Alameh, 2022	●	●	●	●	●	●	○	●	(206)
Li, 2022	●	●	●	●	●	●	○	●	(208)
Rodić, 2022	○	○	●	●	●	●	○	●	(209)
Seal, 2022	○	●	●	●	●	●	●	●	(211)
Shi, 2023	○	○	●	●	●	●	○	●	(212)

Q1: Were patient's demographic characteristics clearly described?; Q2: Was the patient's history clearly described and presented as a timeline? Q3: Was the current clinical condition of the patient on presentation clearly described?; Q4: Were diagnostic tests or methods and the results clearly described?; Q5: Was the intervention(s) or treatment procedure(s) clearly described?; Q6: Was the post-intervention clinical condition clearly described?; Q7: Were adverse events (harms) or unanticipated events identified and described?; Q8: Does the case report provide takeaway lessons? ● = Yes; ○ = No; ⦸ = Unclear; ◌ = Not Available.

Table SVI. Quality assessment of included studies (case series).

First author, year	Q1	Q2	Q3	Q4	Q5	Q6	Q7	Q8	Q9	Q10	(Refs.)
Komaki, 1984	●	●	●	⦸	⦸	●	●	●	●	●	(8)
Gharpure, 1985	●	●	●	⦸	⦸	○	●	●	●	●	(10)
Khoury, 1989	●	●	●	●	⦸	○	●	●	●	●	(19)
Perren, 1992	●	●	●	●	⦸	●	●	●	●	●	(32)
Maryniak, 1993	●	●	⦸	⦸	⦸	○	●	●	○	●	(33)
Aozasa, 1993	●	●	●	●	●	○	●	●	●	●	(34)
Broekmans, 1993	●	●	⦸	⦸	⦸	●	●	●	●	●	(35)
Makarewicz, 1993	●	●	●	●	⦸	○	●	●	●	●	(36)
Kasales, 1994	●	●	●	●	●	○	●	●	○	●	(38)
Makarewicz, 1995	●	●	●	●	⦸	○	●	●	●	●	(43)
Kawakami, 1995	●	●	●	⦸	⦸	○	○	●	●	●	(44)
Holweg, 1995	●	●	●	⦸	⦸	○	●	○	●	●	(46)
al-Talib, 1996	●	●	●	⦸	⦸	○	●	●	○	●	(52)
Lee, 1998	●	●	●	●	⦸	○	●	●	●	●	(55)
Grace, 1999	●	●	●	⦸	⦸	○	●	●	●	●	(60)
Mhawech, 2000	●	●	●	○	○	●	○	●	○	●	(63)
Piura, 2000	●	●	●	⦸	⦸	○	●	●	●	●	(67)
Kaneko, 2001	●	●	●	⦸	⦸	●	●	●	●	●	(69)
Vang, 2001	●	●	●	⦸	●	○	●	●	●	●	(70)
Au, 2003	●	●	●	○	⦸	●	○	●	●	●	(78)
Kendrick, 2005	●	●	●	⦸	⦸	○	●	●	●	●	(86)
Chan, 2005	●	●	●	⦸	●	●	●	●	●	●	(87)
Garavaglia, 2005	●	●	●	⦸	⦸	●	●	●	●	●	(88)
Dursun, 2005	●	●	●	⦸	⦸	○	●	●	●	●	(89)
Heredia, 2005	●	●	●	●	⦸	●	●	●	●	●	(93)
Van Renterghem, 2005	●	●	●	⦸	⦸	○	●	●	●	●	(94)
Frey, 2006	●	●	●	⦸	●	●	●	●	●	●	(97)
Hariprasad, 2006	●	●	●	⦸	⦸	○	●	●	●	●	(101)
Cohn, 2007	●	●	●	⦸	⦸	○	●	●	●	●	(106)
Signorelli, 2007	●	●	●	●	●	●	●	●	●	●	(107)
Köhler, 2008	●	●	●	⦸	⦸	●	●	●	●	●	(116)
Hanley, 2009	●	●	●	⦸	⦸	●	●	○	●	●	(118)
Upanal, 2011	●	●	●	⦸	⦸	●	●	●	●	●	(125)
Dyer, 2011	●	●	●	●	○	○	●	○	●	●	(127)
Kim, 2011	●	●	●	⦸	⦸	○	●	○	●	●	(129)
Chraiet, 2012	●	●	●	⦸	⦸	○	●	●	●	●	(130)
Ramalingam, 2012	●	●	●	⦸	⦸	○	○	●	○	●	(134)
Cao, 2014	●	●	●	●	●	○	●	●	●	●	(146)
Pather, 2015	●	●	●	⦸	⦸	○	○	●	●	●	(155)
Kasai, 2015	●	●	●	⦸	⦸	○	●	●	●	●	(157)
Vijayakumar, 2016	●	●	●	⦸	⦸	○	●	●	●	●	(167)
Singh, 2016	●	●	●	⦸	⦸	○	●	●	●	●	(170)
Liu, 2009	●	●	●	⦸	⦸	○	○	●	●	●	(172)
Goda, 2020	●	●	●	⦸	⦸	○	●	●	●	●	(189)
Murata, 2020	●	●	●	○	⦸	●	●	●	●	●	(193)
Saksena, 2022	●	●	●	●	●	●	●	●	●	●	(205)
Aminimoghaddam, 2022	●	●	●	⦸	⦸	●	●	●	●	●	(207)
Weng, 2022	●	●	●	●	●	○	●	●	●	●	(210)
Gao, 2023	●	●	●	●	●	○	●	●	●	●	(213)

Q1: Were there clear criteria for inclusion in the case series?; Q2: Was the condition measured in a standard, reliable way for all participants included in the case series?; Q3: Were valid methods used for identification of the condition for all participants included in the case series?; Q4: Did the case series have consecutive inclusion of participants?; Q5 Did the case series have complete inclusion of participants?; Q6: Was there clear reporting of the demographics of the participants in the study?; Q7: Was there clear reporting of clinical information of the participants?; Q8: Were the outcomes or follow up results of cases clearly reported?; Q9: Was there clear reporting of the presenting site (s)/clinic (s) demographic information?; Q10: Was statistical analysis appropriate? ● = Yes; ○ = No; ⦸ = Unclear; ◌ = Not available.

Table SVII. Number of included patients in the integrated analysis for overall survival. P-values were derived from the Kaplan-Meier curves that were calculated using the log-rank test and the Gehan-Breslow-Wilcoxon method.

A, Main analysis of overall survival (n=229)		
Group A	Group B	P-value
Premenopausal (n=94)	Postmenopausal (n=80)	0.005
Operative (n=78)	Non-operative (n=139)	0.2
Symptomatic (n=187)	Asymptomatic (n=24)	0.2
Operative (n=78)	Chemoradiation (n=49)	0.8
Surgery only (n=22)	Surgery + adjuvant treatment[a] (n=56)	0.1
Chemotherapy only (n=72)	Chemoradiation (n=49)	0.2
Surgery + adjuvant treatment[a] (n=56)	Chemoradiation (n=49)	0.4
Surgery + chemotherapy (n=24)	Chemotherapy only (n=72)	0.2
Stage I+II (n=104)	Stage III+IV (n=28)	0.1
Stage I (n=70)	Stage IV (n=22)	0.1
Metastasis (n=13)	No metastasis (n=155)	0.5
Operative (n=78)	Chemotherapy only (n=72)	0.1
B, Subgroup analysis of overall survival excluding studies with high risk of bias (n=173)		
Group A	Group B	P-value
Premenopausal (n=66)	Postmenopausal (n=64)	0.002
Operative (n=60)	Non-operative (n=108)	0.3
Symptomatic (n=147)	Asymptomatic (n=14)	0.6
Operative (n=60)	Chemoradiation (n=40)	0.7
Surgery only (n=17)	Surgery + adjuvant treatment[a] (n=43)	0.2
Chemotherapy only (n=55)	Chemoradiation (n=40)	0.4
Surgery + adjuvant treatment[a] (n=43)	Chemoradiation (n=40)	0.8
Surgery + chemotherapy (n=14)	Chemotherapy only (n=55)	0.6
Stage I+II (n=89)	Stage III+IV (n=21)	0.1
Stage I (n=59)	Stage IV (n=17)	0.1
Metastasis (n=11)	No metastasis (n=113)	0.5
Operative (n=60)	Chemotherapy only (n=55)	0.2
C, Subgroup analysis of overall survival of post-menopausal patients only (n=80)		
Group A	Group B	P-value
Operative (n=26)	Non-operative (n=51)	0.8
Symptomatic (n=70)	Asymptomatic (n=7)	n/a
Operative (n=26)	Chemoradiation (n=17)	0.4
Surgery only (n=7)	Surgery + adjuvant treatment[a] (n=19)	0.8
Chemotherapy only (n=30)	Chemoradiation (n=17)	0.2
Surgery + adjuvant treatment[a] (n=19)	Chemoradiation (n=17)	0.4
Surgery + chemotherapy (n=9)	Chemotherapy only (n=30)	0.4
Stage I+II (n=42)	Stage III+IV (n=13)	0.2
Stage I (n=23)	Stage IV (n=12)	0.7
Metastasis (n=13)	No metastasis (n=54)	0.4
Operative (n=26)	Chemotherapy only (n=30)	0.3
D, Subgroup analysis of overall survival of patients with stage I+II only (n=104)		
Group A	Group B	P-value
Premenopausal (n=39)	Postmenopausal (n=42)	0.3
Operative (n=32)	Non-operative (n=70)	0.1
Symptomatic (n=82)	Asymptomatic (n=11)	0.5
Operative (n=32)	Chemoradiation (n=30)	0.1
Surgery only (n=8)	Surgery + adjuvant treatment[a] (n=24)	0.5
Chemotherapy only (n=28)	Chemoradiation (n=30)	0.8
Surgery + adjuvant treatment[a] (n=24)	Chemoradiation (n=30)	0.1
Surgery + chemotherapy (n=8)	Chemotherapy only (n=28)	0.3
Metastasis (n=3)	No metastasis (n=85)	0.9
Operative (n=32)	Chemotherapy only (n=28)	0.1

[a]Chemotherapy or radiotherapy or both.

References

1. NASIELL M. Hodgkin's disease limited to the uterine cervix. A case report including cytological findings in the cervical and vaginal smears. *Acta cytologica* 1964;8:16-18.
2. WG M. MALIGNANT LYMPHOMA OF THE CERVIX. *The Nova Scotia Medical Bulletin* 1965;44:72 PASSIM-72 PASSIM.
3. ANDERSON GG. Hodgkin's disease of the uterine cervix: Report of a case. *Obstetrics & Gynecology* 1967;29(2):170-72.
4. ALANIS C, COLUNGA F. LINFOMA DEL CERVIX UTERINO (INFORME DE DOS CASOS). 1974
5. Carr I, Hill A, Hancock B, et al. Malignant lymphoma of the cervix uteri: histology and ultrastructure. *Journal of Clinical Pathology* 1976;29(8):680-86.
6. Tunca JC, Reddi P, Shah SH, et al. Malignant non-Hodgkin's-type lymphoma of the cervix uteri occurring during pregnancy. *Gynecologic Oncology* 1979;7(3):385-93.
7. Steinfeld AD. Histiocytic lymphoma of the cervix. *Gynecologic Oncology* 1979;8(1):97-103.
8. Komaki R, Cox JD, Hansen RM, et al. Malignant lymphoma of the uterine cervix. *Cancer* 1984;54(8):1699-704.
9. Bowen D, Gordon Grant M. Primary lymphoma of the cervix-a case report. *South African Medical Journal* 1985;68(12):889-90.
10. Gharpure K, Mahesh D, Bhargava M. Malignant lymphoma of the uterine cervix--a report of two cases with review of literature. *Indian journal of cancer* 1985;22(4):296-302.
11. Taki I, Aozasa K, Kurokawa K. Malignant lymphoma of the uterine cervix. Cytologic diagnosis of a case with immunocytochemical corroboration. *Acta cytologica* 1985;29(4):607-11.
12. Bär BM, Reijnders FJ, Keuning JJ, et al. Primary malignant lymphoma of the uterine cervix associated with cold-reacting autoantibody-mediated hemolytic anemia. *Acta haematologica* 1986;75(4):232-35.
13. Mann R, Roberts WS, Gunasakeran S, et al. Primary lymphoma of the uterine cervix. *Gynecologic oncology* 1987;26(1):127-34.
14. Cardillo M, Manente L, Ambrad O, et al. Immunohistochemical study in a case of primitive lymphoma of the uterine cervix. *European journal of gynaecological oncology* 1987;8(6):607-12.
15. Shimamoto T, Tateyama H. Case report of malignant lymphoma of the uterine cervix. *Nihon Sanka Fujinka Gakkai Zasshi* 1987;39(6):1013-16.
16. Strang P, Sorbe B, Sundström C. Primary aneuploid lymphoma of the uterine cervix: a case report. *Gynecologic oncology* 1988;30(2):302-05.
17. Ibrahim Z, Mellor S, Curtis M. Primary lymphoma of the cervix. *Journal of Obstetrics and Gynaecology* 1988;8(4):364-65.
18. Khong C, Yam K, Ong B. Primary malignant lymphoma of the cervix uteri: a case report. *Singapore medical journal* 1989;30(2):217-20.
19. Robinson A, Khoury G, Carney D. Burkitt's lymphoma following treatment for Hodgkin's disease. *The British Journal of Radiology* 1989;62(744):1089-90.
20. Kurup A, Viegas O, Wee A, et al. Primary lymphoma of the cervix: a case report. *Materia Medica Polona Polish Journal of Medicine and Pharmacy* 1989;21(1):31-33.

21. Hashimoto A, Fujimi A, Kanisawa Y, et al. Primary diffuse large B-cell lymphoma of the uterine cervix successfully treated with rituximabplus cyclophosphamide, doxorubicin, vincristine, and prednisone chemotherapy-a case report. *Gan to Kagaku Ryoho Cancer & Chemotherapy* 2013;40(13):2589-92.
22. Campbell J, Pace D, O'brien C, et al. Primary peripheral T cell lymphoma of the cervix. *Journal of Obstetrics and Gynaecology* 1989;10(2):160-61.
23. Johnston C, Senekjian EK, Ratain MJ, et al. Conservative management of primary cervical lymphoma using combination chemotherapy: a case report. *Gynecologic oncology* 1989;35(3):391-94.
24. Matsuyama T, Tsukamoto N, Kaku T, et al. Primary malignant lymphoma of the uterine corpus and cervix. Report of a case with immunocytochemical analysis. *Acta cytologica* 1989;33(2):228-32.
25. Murotsuki J, Yazaki S, Hayakawa S, et al. A case of malignant lymphoma of the uterine cervix. *Gan no rinsho Japan Journal of Cancer Clinics* 1989;35(14):1716-20.
26. Hochmalignes Non-Hodgkin-Lymphom der Cervix uteri. Gynäkologie und Geburtshilfe 1988; 1989. Springer.
27. Mathiasen H, Mygind H, Agertoft L. Malignant lymphoma of the uterine cervix. *Ugeskrift for Laeger* 1990;152(6):401-01.
28. Dang HT, Terk MR, Colletti PM, et al. Primary lymphoma of the cervix: MRI findings with gadolinium. *Magnetic resonance imaging* 1991;9(6):941-44.
29. Ohkuma Y, Hachisuga T, Fukuda K, et al. 23 Examination of lymphoma-like lesion of the uterine cervix. *日本産科婦人科學會雜誌* 1991;43(8):1133.
30. Pasini F, Iuzzolino P, Santo A, et al. A primitive lymphoma of the uterine cervix. Case report. *European journal of gynaecological oncology* 1991;12(2):107-12.
31. Malatskey A, Reuter KL, Woda B. Sonographic findings in primary uterine cervical lymphoma. *Journal of clinical ultrasound* 1991;19(1):62-64.
32. Perren T, Farrant M, McCarthy K, et al. Lymphomas of the cervix and upper vagina: a report of five cases and a review of the literature. *Gynecologic oncology* 1992;44(1):87-95.
33. Maryniak R, Nasierowska-Guttmejer A. Primary malignant lymphoma of the uterine cervix. A clinicopathological evaluation of 3 cases. *European journal of gynaecological oncology* 1993;14(5):402-05.
34. Aozasa K, Saeki K, Ohsawa M, et al. Malignant lymphoma of the uterus. Report of seven cases with immunohistochemical study. *Cancer* 1993;72(6):1959-64.
35. Broekmans F, Swartjes J, Van der Valk P, et al. Primary malignant lymphoma of the uterus: localization in a cervical polyp. *European Journal of Obstetrics & Gynecology and Reproductive Biology* 1993;48(3):215-19.
36. Makarewicz R, Swirska M, Terlikiewicz J. Non-hodgkin's malignant lymphoma in the uterine cervix. *Wiadomosci Lekarskie (Warsaw, Poland: 1960)* 1993;46(23-24):936-37.
37. Rodier J, Ghnassia J, Janser J, et al. Non-Hodgkin's malignant lymphoma of the cervix uteri. *Journal de chirurgie* 1993;130(12):554-55.
38. Kasales C, Langer J, Young C, et al. Residual mass after treatment of primary cervical lymphoma: the utility of gallium 67 and MRI. *Abdominal imaging* 1994;19:274-77.
39. Cuykx H, Oyen R, Knaepen C, et al. Primary lymphoma of the uterine cervix. *Journal belge de radiologie* 1994;77(1):13-14.

40. Awwad JT, Khalil AM, Shamseddine AI, et al. Primary malignant lymphoma of the uterine cervix: is radiotherapy the best therapeutic choice for stage IE? *Gynecologic oncology* 1994;52(1):91-93.
41. HUH SJ, CHO CK, AHN YC, et al. PRIMARY MALIGNANT LYMPHOMA OF THE UTERINE CERVIX. *The Journal of JASTRO* 1994;6(2):119-23.
42. Patsner B, Greenberg S. Unsuspected Primary Cervical Lymphoma Presenting as" Cervical Fibroid": Partial Response to Leuprolide Acetate. *Gynecologic oncology* 1995;58(3):393-94.
43. Makarewicz R, Kuzminska A. Non-Hodgkin's lymphoma of the uterine cervix: a report of three patients. *Clinical Oncology* 1995;7(3):198-99.
44. Kawakami S, Togashi K, Kojima N, et al. MR appearance of malignant lymphoma of the uterus. *Journal of computer assisted tomography* 1995;19(2):238-42.
45. Gupta S, Sachdev L, Gupta R, et al. Non-Hodgkin lymphoma of the uterine cervix. *Annals of Saudi Medicine* 1995;15(2):187-88.
46. Holweg M KA, Stegner HE, Loning T, Jonat W. Primary lymphoma of the cervix uteri--2 case reports. *Geburtshilfe und Frauenheilkunde* 1995;55(3):171-72.
47. Plewicka M, Szelc S, Smok-Ragankiewicz A, et al. Non-Hodgkin's lymphoma of the uterine cervix. *Ginekologia polska* 1995;66(4):246-48.
48. Papadopoulos A, Pambakian H, Devaja O, et al. High grade non-Hodgkins stage IEB primary malignant lymphoma of the cervix and upper vagina. A case report. *European journal of gynaecological oncology* 1996;17(6):484-86.
49. Abbas MA, Birdwell R, Katz DS, et al. Primary lymphoma of the cervix in a heart transplant patient. *AJR American journal of roentgenology* 1996;167(5):1136-38.
50. Dhimes P, Alberti N, De Agustin P, et al. Primary malignant lymphoma of the uterine cervix: report of a case with cytologic and immunohistochemical diagnosis. *Cytopathology* 1996;7(3):204-10.
51. La Fianza A, Di Maggio E, Preda L, et al. Role of computerized tomography (CT) in the diagnosis and treatment of primary lymphoma of the cervix uteri: report of 2 cases. *Radiol Med* 1996;91(5):662-65.
52. Al-Talib R, Sworn M, Ramsay A, et al. Primary cervical lymphoma: the role of cervical cytology. *Cytopathology* 1996;7(3):173-77.
53. Biswal B, Sharma A, Sharma M, et al. Malignant lymphoma of the uterine cervix: a case report and review of literature. *Journal of the Indian Medical Association* 1997;95(7):434-40.
54. El Ghazi E, Mansouri H, Melhouf M, et al. Non Hodgkin's malignant lymphoma of the uterine cervix. Apropos of a case. *Bulletin du Cancer* 1997;84(4):421-22.
55. Lee K, Seah E, Sethi V. Primary non-Hodgkin's lymphoma of the uterine cervix: Case report of long-term survival of two patients treated with surgery and radiotherapy. *Australasian radiology* 1998;42(2):126-27.
56. Chandy L, Kumar L, Dawar R. Non-Hodgkin's Lymphoma Presenting as a Primary Lesion in Uterine Cervix: Case Report. *Journal of Obstetrics and Gynaecology Research* 1998;24(3):183-87.
57. Clarke D, Ostler P, Watkinson A, et al. Case report: magnetic resonance imaging in primary cervical lymphoma: the role in diagnosis and follow-up. *Clinical radiology* 1998;53(5):383-85.

58. Kaito K, Otsubo H, Sekita T, et al. Primary non-Hodgkin's lymphoma of the uterine cervix complicated by acute renal failure due to ureter obstruction. *[Rinsho Ketsueki] The Japanese Journal of Clinical Hematology* 1998;39(6):463-65.
59. Bilgin T, Dos A, Tolunay S. Primary malignant lymphoma of the uterine cervix: difficulties in diagnosis. *Journal of Obstetrics and Gynaecology* 1999;19(6):671.
60. Grace A, O'Connell N, Byrne P, et al. Malignant lymphoma of the cervix. An unusual presentation and a rare disease. *European journal of gynaecological oncology* 1999;20(1):26-28.
61. Wang PH, Chao KC, Lin G, et al. Primary malignant lymphoma of the cervix in pregnancy. A case report. *The Journal of reproductive medicine* 1999;44(7):630-32.
62. Isosaka M, Hayashi T, Mitsuhashi K, et al. Primary diffuse large B-cell lymphoma of the uterus complicated with hydronephrosis. *[Rinsho Ketsueki] The Japanese Journal of Clinical Hematology* 2013;54(4):392-96.
63. Mhawech P, Medeiros LJ, Bueso-Ramos C, et al. Natural Killer–Cell Lymphoma Involving the Gynecologic Tract. *Archives of pathology & laboratory medicine* 2000;124(10):1510-13.
64. Agarossi A, Ridolfo A, Antonacci M, et al. Primary malignant lymphoma of the uterine cervix in a patient with AIDS. *ITALIAN JOURNAL OF GYNAECOLOGY AND OBSTETRICS* 2000;12:119-22.
65. Kostopoulos IS, Barbanis SB, Kaloutsi VD, et al. Synchronous occurrence of multiple malignant neoplasms in the uterus (adenocarcinoma of the endometrium, large B-cell lymphoma of the cervix). *Pathology-Research and Practice* 2000;196(8):573-75.
66. Yokoyama Y, Sato S, Xiao Y, et al. Primary non-Hodgkin's lymphoma of the uterine cervix. *Archives of gynecology and obstetrics* 2001;265:108-11.
67. Piura B, Yanai-Inbar I, Rabinovich A, et al. Lymphoma-like lesion of the uterine cervix. *European Journal of Obstetrics & Gynecology and Reproductive Biology* 2001;97(2):235-38.
68. Mansouri H, Kebdani T, Hassouni K, et al. Unusual locations for lymphomas: Case 1. Intermediate-grade lymphoma of the cervix. *Journal of clinical oncology* 2001;19(11):2959-60.
69. Kaneko H, Kita Y, Taniwaki M, et al. Clinicopathologic and cytogenetic analyses of three cases of primary uterine non-Hodgkin's lymphoma. *Internal medicine* 2001;40(10):1028-31.
70. Vang R, Medeiros LJ, Fuller GN, et al. Non-Hodgkin's lymphoma involving the gynecologic tract: a review of 88 cases. *Advances in anatomic pathology* 2001;8(4):200-17.
71. Jha V, Cuthbert R, Jackson R, et al. Non-Hodgkin's lymphoma of the cervix and vagina presenting with bilateral hydronephrosis and acute renal failure. *JOURNAL OF OBSTETRICS AND GYNAECOLOGY-BRISTOL-* 2001;21(1):83-83.
72. Rossi G, Bonacorsi G, Longo L, et al. Primary High-Grade Mucosa-Associated Lymphoid Tissue–Type Lymphoma of the Cervix Presenting as a Common Endocervical Polyp. *Archives of pathology & laboratory medicine* 2001;125(4):537-40.
73. Liro M, Emerich J, Debniak J, et al. A rare case of non-Hodgkin's lymphoma of the uterine cervix. *Ginekologia polska* 2001;72(1):27-30.
74. Bode M, Tikkakoski T, Johansson J, et al. Lymphoma of the cervix: Imaging and transcatheter arterial embolization. *Acta Radiologica* 2002;43(4):431-32.

75. Marín C, Seoane JM, Sánchez M, et al. Magnetic resonance imaging of primary lymphoma of the cervix. *European radiology* 2002;12:1541-45.
76. Lyman MD, Neuhauser TS. Precursor T-cell acute lymphoblastic leukemia/lymphoma involving the uterine cervix, myometrium, endometrium, and appendix. *Annals of Diagnostic Pathology* 2002;6(2):125-28.
77. el Mrabet F, Ferhati D, Berkhli S, et al. Primary malignant non-Hodgkin's lymphoma of the cervix uteri. *Presse Medicale (Paris, France: 1983)* 2002;31(7):318-18.
78. Au W, Chan B, Chung L, et al. Primary B-cell lymphoma and lymphoma-like lesions of the uterine cervix. *American journal of hematology* 2003;73(3):176-79.
79. Baxter NP, Lane G, Swift S. Primary malignant follicular lymphoma of the cervix: a rare cause of postmenopausal bleeding. *BJOG: An International Journal of Obstetrics & Gynaecology* 2003;110(3):337-38.
80. Gabriele A, Gaudiano L. Primary malignant lymphoma of the cervix. A case report. *The Journal of reproductive medicine* 2003;48(11):899-901.
81. Kahlifa M, Buckstein R, Perez-Ordoñez B. Sarcomatoid variant of B-cell lymphoma of the uterine cervix. *International journal of gynecological pathology* 2003;22(3):289-93.
82. Szánthó A, ános Bálega J, Csapó Z, et al. Primary non-Hodgkin's lymphoma of the uterine cervix successfully treated by neoadjuvant chemotherapy: case report. *Gynecologic oncology* 2003;89(1):171-74.
83. Quattrini M, Del Nonno F, Pacetti U, et al. Rapid modification of aggressiveness of a primary non-Hodgkin lymphoma of uterine cervix. *Journal of Experimental & Clinical Cancer Research: CR* 2003;22(4):633-35.
84. Sun S-S, Lin C-Y, Chuang F-J, et al. Ga-67 scintigraphy in primary cervical lymphoma. *Clinical nuclear medicine* 2003;28(10):869-70.
85. Thyagarajan M, Dobson M, Biswas A. Appearance of uterine cervical lymphoma on MRI: a case report and review of the literature. *The British journal of radiology* 2004;77(918):512-15.
86. Kendrick IV JE, Straughn Jr JM. Two cases of non-Hodgkin's lymphoma presenting as primary gynecologic malignancies. *Gynecologic oncology* 2005;98(3):490-92.
87. Chan JK, Loizzi V, Magistris A, et al. Clinicopathologic features of six cases of primary cervical lymphoma. *Am J Obstet Gynecol* 2005;193(3):866-72.
88. Garavaglia E, Taccagni G, Montoli S, et al. Primary stage I–IIE non-Hodgkin's lymphoma of uterine cervix and upper vagina: evidence for a conservative approach in a study on three patients. *Gynecologic oncology* 2005;97(1):214-18.
89. Dursun P, Gultekin M, Bozdag G, et al. Primary cervical lymphoma: report of two cases and review of the literature. *Gynecol Oncol* 2005;98(3):484-89.
90. Alameda F, Bellosillo B, Baró T, et al. Large cell lymphoma-like reaction in a cervical polyp. *Gynecologic oncology* 2005;99(2):481-85.
91. Goker B, Bese T, Ilvan S, et al. A case with multiple gynecological malignancies. *International Journal of Gynecologic Cancer* 2005;15(2)
92. Murad M, Akhtar W. Primary lymphoma of the cervix. *Journal of the College of Physicians and Surgeons--pakistan: JCPSP* 2005;15(6):364-65.

93. Heredia F, Bravo M, Pierotic M, et al. Neoadjuvant combined chemotherapy followed by external whole pelvic irradiation in two cases of primary extranodal non-Hodgkin's lymphoma of the uterine cervix. *Gynecologic oncology* 2005;97(1):285-87.

94. Van Renterghem N, De Paepe P, Van den Broecke R, et al. Primary lymphoma of the cervix uteri: a diagnostic challenge. Report of two cases and review of the literature. *European journal of gynaecological oncology* 2005;26(1):36-38.

95. Huang W-T, Chuang S-S, Eng H-L, et al. Synchronous CIN 3 and cervical lymphoma: a case report and review of the literature. *Pathology-Research and practice* 2005;201(7):521-26.

96. Synchronous primary cervical lymphoma and HPV infection with low grade intraepithelial neoplasia. A case report. MODERN PATHOLOGY; 2006. NATURE PUBLISHING GROUP 75 VARICK STREET, 9TH FLOOR, NEW YORK, NY 10013-1917 USA.

97. Frey NV, Svoboda J, Andreadis C, et al. Primary lymphomas of the cervix and uterus: the University of Pennsylvania's experience and a review of the literature. *Leukemia & lymphoma* 2006;47(9):1894-901.

98. González-Cejudo C, Martínez-Maestre MA, Peregrín-Álvarez I, et al. Primary lymphoma of the cervix: unusual location for a common disease. *European Journal of Obstetrics and Gynecology and Reproductive Biology* 2006;125(2):268-69.

99. Gupta S, Maheshwari A, Bhati GS, et al. Cervical lymphoma presenting as irregular vaginal bleeding. *Journal of Cancer Research and Therapeutics* 2006;2(2):72-73.

100. Semczuk A, Skomra D, Korobowicz E, et al. Primary non-Hodgkin's lymphoma of the uterine cervix mimicking leiomyoma: case report and review of the literature. *Pathology-Research and Practice* 2006;202(1):61-64.

101. Hariprasad R, Kumar L, Bhatla DN, et al. Primary uterine lymphoma: report of 2 cases and review of literature. *American journal of obstetrics and gynecology* 2006;195(1):308-13.

102. De LeÓN DC, Montiel DP, Vilchis JC. Primary malignant lymphoma of uterine cervix. *International Journal of Gynecologic Cancer* 2006;16(2)

103. Paul P, Koshy A, Thomas T. Lymphoma of the cervix: a case report with review of literature. *Gynecological Surgery* 2006;3(3):226-27.

104. Wannesson L. Primary lymphoma of the uterine cervix: An approach to management. *Gynecologic oncology* 2006;100(3):626-27.

105. Bural GG, Shriaknthan S, Houseni M, et al. FDG-PET is useful in staging and follow-up of primary uterine cervical lymphoma. *Clinical Nuclear Medicine* 2007;32(9):748-50.

106. Cohn D, Resnick K, Eaton L, et al. Non-Hodgkin's lymphoma mimicking gynecological malignancies of the vagina and cervix: a report of four cases. *International Journal of Gynecologic Cancer* 2007;17(1)

107. Signorelli M, Maneo A, Cammarota S, et al. Conservative management in primary genital lymphomas: the role of chemotherapy. *Gynecologic oncology* 2007;104(2):416-21.

108. Korcum AF, Karadogan I, Aksu G, et al. Primary follicular lymphoma of the cervix uteri: a review. *Annals of hematology* 2007;86:623-30.

109. Lorusso D, Ferrandina G, Pagano L, et al. Successful pregnancy in stage IE primary non-Hodgkin’s lymphoma of uterine cervix treated with neoadjuvant chemotherapy and conservative surgery. *Oncology* 2008;72(3-4):261-64.

110. Hodgkin's lymphoma of the uterine cervix. HAEMATOLOGICA-THE HEMATOLOGY JOURNAL; 2007. FERRATA STORTI FOUNDATION VIA GIUSEPPE BELLI 4, 27100 PAVIA, ITALY.
111. Coon D, Beriwal S, Swerdlow SH, et al. Mucosa-associated lymphoid tissue lymphoma of the cervix. *Journal of clinical oncology* 2008;26(3):503-04.
112. Ab Hamid S, Wastie M. Primary non-Hodgkin's lymphoma presenting as a uterine cervical mass. *Singapore Med J* 2008;49(3):e73-e75.
113. Okudaira T, Nagasaki A, Miyagi T, et al. Primary diffuse large B-cell lymphoma of the uterine cervix--a case report. *Gan to kagaku ryoho Cancer & chemotherapy* 2008;35(8):1423-25.
114. Hanprasertpong J, Hanprasertpong T, Thammavichit T, et al. Primary non-Hodgkin, s lymphoma of the uterine cervix. *Asian Pac J Cancer Prev* 2008;9(2):363-66.
115. Su CF, Tsai HJ, Kuo C, et al. Primary non-Hodgkin's lymphoma of the uterus, cervix and parametrium treated by combined immunochemotherapy. *Journal of Obstetrics and Gynaecology Research* 2008;34(4pt2):749-53.
116. Köhler HF, Novik PR, Campos AHJFM. Lymphoma of the uterine cervix: report of two cases and review of the literature. *Revista Brasileira de Ginecologia e Obstetrícia* 2008;30:626-30.
117. Demuynck F, Drean Y, Morvan J, et al. Primitive lymphoma of the uterine cervix: 3-T MR diffusion imaging and PET-CT features. *European Journal of Radiology Extra* 2009;71(1):e33-e36.
118. Hanley KZ, Tadros TS, Briones AJ, et al. Hematologic malignancies of the female genital tract diagnosed on liquid-based Pap test: Cytomorphologic features and review of differential diagnoses. *Diagnostic cytopathology* 2009;37(1):61-67.
119. Baijal G, Vadiraja B, Fernandes DJ, et al. Diffuse large B-cell lymphoma of the uterine cervix: a rare case managed novelly. *Journal of Cancer Research and Therapeutics* 2009;5(2):140-42.
120. Amna FA, Howell R, Raj S. Lymphoma of the cervix uteri. *Case Reports* 2009;2009:bcr0520091835.
121. Wang Z-Y, Li Y-X, Wang W-H, et al. Primary radiotherapy showed favorable outcome in treating extranodal nasal-type NK/T-cell lymphoma in children and adolescents. *Blood, The Journal of the American Society of Hematology* 2009;114(23):4771-76.
122. Naki MM, Çelik H, Api O, et al. Primary uterine lymphoma of the uterine cervix in advanced age/Ileri yasta uterus serviksinde olusan primer uterus lenfomasi. *Turkish Journal of Haematology* 2010;27(3):219.
123. Ustaalioglu BBO, Bilici A, Seker M, et al. Primary non-Hodgkin lymphoma of cervix successfully treated with rituximab: positron emission tomography images before and after therapy: a case report. *Leukemia research* 2010;4(34):e108-e10.
124. Novotny S, Ellis T, Stephens J. Primary B-cell lymphoma of the cervix presenting with bilateral hydronephrosis. *Obstetrics & Gynecology* 2011;117(2 Part 2):444-46.
125. Upanal N, Enjeti A. Primary lymphoma of the uterus and cervix: two case reports and review of the literature. *Australian and New Zealand Journal of Obstetrics and Gynaecology* 2011;51(6):559-62.
126. Venizelos I, Zafrakas M, Dragoumis K, et al. Non-Hodgkin's lymphoma involving the uterine cervix after treatment for Hodgkin disease. *Leukemia & lymphoma* 2003;44(12):2155-57.
127. Dyer MJ, Ye H, Isaacson PG. Primary lymphoma-like lesions of the uterine cervix; sheep in wolves' clothing. *British journal of haematology* 2011;153(6):791-94.

128. Valizadeh N, Ayatollahi H, Naji S. Primary Non-Hodgkin Lymphoma of The Cervix. *International Journal of Hematology-Oncology and Stem Cell Research* 2011:28-29.
129. Kim J, Kim H, Hong SR, et al. Liquid-Based Pap Smear Findings of Uterine Cervical Lymphoma: Three Cases Report. *Journal of Pathology and Translational Medicine* 2011;45(4):437-40.
130. Chraiet N, Lagha A, Krimi S, et al. Non-Hodgkin's Lymphoma Mimicking Gynecologic Malignancies of the Uterine Corpus, Cervix, and Vagina: A Report of Three Cases. *Journal of Gynecologic Surgery* 2012;28(2):158-60.
131. Park SB, Lee YH, Song MJ, et al. Sonographic findings of uterine cervical lymphoma manifesting as multinodular lesions. *Clinical Imaging* 2012;36(5):636-38.
132. Binesh F, Vahedian H, Rajabzadeh Y. Primary malignant lymphoma of the uterine cervix. *Case Reports* 2012;2012:bcr2012006675.
133. Calli AO, Rezanko T, Yigit S, et al. Lymphoma of the cervix: A diagnostic pitfall on cervicovaginal smear. *Journal of Cytology/Indian Academy of Cytologists* 2012;29(3):213.
134. Ramalingam P, Zoroquiain P, Valbuena JR, et al. Florid reactive lymphoid hyperplasia (lymphoma-like lesion) of the uterine cervix. *Annals of Diagnostic Pathology* 2012;16(1):21-28.
135. Jastaniyah N, Lai R, Pearcey R. Nodular lymphocyte predominant Hodgkin's lymphoma of the cervix: A case report of a rare entity. *Gynecologic Oncology Case Reports* 2013;4:4.
136. Kanaan D, Parente DB, Constantino CPL, et al. Lymphoma of uterine cervix: magnetic resonance imaging findings. *Radiologia Brasileira* 2012;45:167-69.
137. Ledwich LJ, Olenginski TP. A rare lymphoma in a patient with amyopathic dermatomyositis. *American journal of clinical dermatology* 2010;11:151-55.
138. Parnis J, Camilleri DJ, Babic D, et al. Lymphoma of the cervix. *Case Reports in Hematology* 2012;2012
139. Yalta T, Taştekin E, Puyan FÖ, et al. Non-Hodgkin's lymphoma: A rare diagnosis on cervicovaginal cytology. *Journal of Cytology/Indian Academy of Cytologists* 2012;29(2):142.
140. Yin W-H, Guo N, Tian X-Y, et al. Pediatric Anaplastic Lymphoma Kinase–Positive Large B-cell Lymphoma: A Case Report and Review of the Literature. *Pediatric and Developmental Pathology* 2012;15(4):318-23.
141. Bull L, Knowles A, Ogden S, et al. Primary cervical lymphoma: a rare presentation to a genitourinary medicine clinic. *International journal of STD & AIDS* 2013;24(7):587-89.
142. Anagnostopoulos A, Mouzakiti N, Ruthven S, et al. Primary cervical and uterine corpus lymphoma; a case report and literature review. *International Journal of Clinical and Experimental Medicine* 2013;6(4):298.
143. Groszmann Y, Benacerraf BR. Sonographic features of primary lymphoma of the uterine cervix. *Journal of ultrasound in medicine: official journal of the American Institute of Ultrasound in Medicine* 2013;32(4):717-18.
144. Mouhajir N, Diakité A, Toulba A, et al. Primary non-Hodgkin's lymphoma of the uterine cervix: case report of long-term survival patient. *The Journal of Obstetrics and Gynecology of India* 2014;64:145-47.
145. Primary B lymphoma of the uterine cervix. VIRCHOWS ARCHIV; 2013. SPRINGER 233 SPRING ST, NEW YORK, NY 10013 USA.
146. Cao X-x, Li J, Zhang W, et al. Patients with primary diffuse large B-cell lymphoma of female genital tract have high risk of central nervous system relapse. *Annals of hematology* 2014;93:1001-05.

147. Bellevicine C, Zabatta A, Malapelle U, et al. Diffuse Large B-Cell Extranodal Lymphoma of the Uterine Cervix: An Incidental Pap Smear Finding With Histological and Immunohistochemical Correlation. *Diagnostic Cytopathology* 2014;42(7):644-46.

148. De Greve T, Vanwalleghem L, Van Hoof A, et al. An unusual cervical tumor as presentation of a non-Hodgkin lymphoma. *Case Reports in Obstetrics and Gynecology* 2014;2014

149. Igwe E, Diaz J, Ferriss J. Diffuse large B cell lymphoma of the cervix with rectal involvement. *Gynecologic Oncology Reports* 2014;10:1.

150. Korivi BR, Jensen CT, Patnana M, et al. A rare presentation of lymphoma of the cervix with cross-sectional imaging correlation. *Case Reports in Radiology* 2014;2014

151. Mandato VD, Palermo R, Falbo A, et al. Primary diffuse large B-cell lymphoma of the uterus: case report and review. *Anticancer Research* 2014;34(8):4377-90.

152. del Valle-Rubido C, Cano-Cuetos A, Heras-Sedano I, et al. Linfoma primario de cérvix: gestación tras tratamiento conservador. *Progresos de Obstetricia y Ginecología* 2014;57(5):225-29.

153. Adachi S, Yamazaki K, Liang S-G, et al. Primary uterine diffuse large B-cell lymphoma involving the urinary bladder with urinary cytology mimicking carcinomas: A case report. *Journal of Cytology/Indian Academy of Cytologists* 2015;32(3):181.

154. Wang G-N, Zhao W-G, Gao X-Z, et al. Primary natural killer/T cell lymphoma of the cervix: case report and clinicopathological analysis. *Taiwanese Journal of Obstetrics and Gynecology* 2015;54(1):71-74.

155. Pather S, Philip V, Lakha AB, et al. An expanded spectrum of high-grade B-cell non-hodgkin lymphomas involving the cervicovaginal region. *International Journal of Gynecological Pathology* 2015;34(6):564-69.

156. Agarwal P, Agarwal G, Singh D, et al. Primary Cervico-Vaginal B-cell Lymphoma with Immunohistochemical Confi rmation: A Case Study. *INTERNATIONAL JOURNAL OF SCIENTIFIC STUDY* 2014;2(8):201-07.

157. Kasai M, Ichimura T, Murakami M, et al. Two cases of uterine malignant lymphoma diagnosed by needle biopsy. *Journal of Obstetrics and Gynaecology Research* 2015;41(10):1664-68.

158. Bilimoria F, Annunziata J, Balog A, et al. "These Are Not The Tumor Cells You Are Looking For": A Case Study of a Primary Non-Hodgkin Lymphoma Arising in the Cervix. *American Journal of Clinical Pathology* 2016;146(suppl_1):144.

159. Dobrosavljevic A, Skrobic M, Stanojevic D, et al. Primary non-Hodgkin lymphoma of the uterine cervix of a follicular type–case report. *Journal of Obstetrics and Gynaecology* 2016;36(5):685-86.

160. Okamura I, Ikeda T. Epstein-Barr virus associated with a lymphoma-mimicking lesion of the uterine cervix. *Blood, The Journal of the American Society of Hematology* 2016;128(10):1439-39.

161. Fratoni S, Abruzzese E, Trawinska MM, et al. Primitive "Spindle Cell Variant"(Sarcomatoid Variant) Diffuse Large B-Cell Lymphoma of the Uterine Cervix: Description and Outcome of a Rare Case. *International Journal of Gynecological Pathology* 2016;35(6):593-97.

162. Hilal Z, Hartmann F, Dogan A, et al. Lymphoma of the cervix: case report and review of the literature. *Anticancer Res* 2016;36(9):4931-40.

163. Omori M, Oishi N, Nakazawa T, et al. Extranodal NK/T-cell lymphoma, nasal type of the uterine cervix: A case report. *Diagnostic cytopathology* 2016;44(5):430-33.

164. An Incidental Diffuse Lymphoma Of The Uterine Cervix. INTERNATIONAL JOURNAL OF GYNECOLOGICAL CANCER; 2016. LIPPINCOTT WILLIAMS & WILKINS TWO COMMERCE SQ, 2001 MARKET ST, PHILADELPHIA ….
165. Sharma V, Dora T, Patel M, et al. Case report of diffuse large B cell lymphoma of uterine cervix treated at a semiurban cancer centre in North India. *Case Reports in Hematology* 2016;2016
166. Sun L-M, Jin A-L, Xu Y-T, et al. Expression of p63 in primary diffuse large B-cell lymphoma of the uterine cervix mimicking low differentiated squamous cell carcinoma. *INTERNATIONAL JOURNAL OF CLINICAL AND EXPERIMENTAL PATHOLOGY* 2016;9(11):12003-07.
167. Vijayakumar S, Serur E, Bybordi B, et al. Primary gynecological non-Hodgkin's lymphoma: a differential diagnosis of a pelvic mass. *Gynecologic oncology reports* 2016;18:29-31.
168. Yang L, Shi T, Liu F, et al. REV3L, a promising target in regulating the chemosensitivity of cervical cancer cells. *PLoS One* 2015;10(3):e0120334.
169. Regalo A, Caseiro L, Pereira E, et al. Primary lymphoma of the uterine cervix: a rare constellation of symptoms. *Case Reports* 2016;2016:bcr2016216597.
170. Singh L, Madan R, Benson R, et al. Primary non-Hodgkins lymphoma of uterine cervix: a case report of two patients. *The Journal of Obstetrics and Gynecology of India* 2016;66:125-27.
171. Srivastava P, Ahluwalia C, Zaheer S, et al. Primary non-Hodgkin's lymphoma of the female genital tract in a 27-year-old female: A rare case report. *Clin Cancer Investig J* 2016;5:287-90.
172. Liu Z, Krabill K, Sands A, et al. Primary Uterine Cervical High-Grade Follicular Lymphoma and Diffuse Large B-Cell Lymphoma: A Case Report and Review of Literature. *American Journal of Clinical Pathology* 2016;146(suppl_1):150.
173. Zhou W, Hua F, Zuo C, et al. Primary uterine cervical lymphoma manifesting as menolipsis staged and followed up by FDG PET/CT. *Clinical Nuclear Medicine* 2016;41(7):590-93.
174. Kosari F, Niknejad N, Nili F, et al. Peripheral T-cell lymphoma presenting as a primary uterine cervix mass: a report of a rare case. *International Journal of Gynecological Pathology* 2017;36(6):523-27.
175. Yang G, Deisch J, Tavares M, et al. Primary B-cell lymphoma of the uterine cervix: Presentation in Pap-test slide and cervical biopsy. *Diagnostic Cytopathology* 2017;45(3):235-38.
176. Azarhoush R, Pourkhani AH, Shahmirzadi AR, et al. Case Report: Primary Lymphoma of The Cervix. *RESEARCH JOURNAL OF PHARMACEUTICAL BIOLOGICAL AND CHEMICAL SCIENCES* 2017;8(3):11-16.
177. Chen Y, Li L, Lu J, et al. Case Report Primary natural killer/T-cell lymphoma of the cervix: a case report and literature review. *Int J Clin Exp Med* 2017;10(2):3899-905.
178. Cubo AM, Soto ZM, Cruz MÁ, et al. Primary diffuse large B cell lymphoma of the uterine cervix successfully treated by combined chemotherapy alone: a case report. *Medicine* 2017;96(19)
179. Roberts ME, Cottrill HM. A case of primary cervical lymphoma in a patient with abnormal uterine bleeding. *Gynecologic oncology reports* 2018;26:105-07.
180. Koyanagi T, Kondo H, Toyama A, et al. Malignant lymphoma of the uterine cervix presumptively diagnosed by Pap smear: A case report. *Oncology Letters* 2018;15(5):6678-80.
181. Seresht LM, Yousefi Z, Davachi B, et al. Primary Malignant Lymphoma of Cervix: A Case Report. *Journal of Midwifery & Reproductive Health* 2019;7(4)

182. Bolandi S. EP805 Primary malignant lymphoma of cervix misdiagnosed as an ovarian cyst on ultrasonography: a case report: BMJ Specialist Journals, 2019.
183. Gui W, Li J, Zhang Z, et al. Primary hematological malignancy of the uterine cervix: A case report. *Oncology Letters* 2019;18(3):3337-41.
184. Heremans R, Froyman W, Beckers M, et al. Advanced Doppler imaging and trucut biopsy of a late-stage cervical lymphoma. *Australasian Journal of Ultrasound in Medicine* 2019;22(4):301-04.
185. Needs T, Bonneau P, Cardoza-Favarato G. Diffuse Large B-Cell Lymphoma of the Cervix Diagnosed on Cervical Screening Cytology. *American Journal of Clinical Pathology* 2019;152:S97.
186. Wilkie GL, Taggart AA, Prensner JR, et al. Burkitt lymphoma presenting as menorrhagia and a vaginal mass in an adolescent. *Journal of pediatric and adolescent gynecology* 2019;32(1):90-92.
187. Costa PA, Needelman BS, Tjendra Y, et al. High-grade B-cell lymphoma with MYC and BCL6 rearrangements presenting as a cervical mass. *BMJ Case Reports CP* 2020;13(8):e235451.
188. Del M, Angeles MA, Syrykh C, et al. Primary B-Cell lymphoma of the uterine cervix presenting with right ureter hydronephrosis: A case report. *Gynecologic Oncology Reports* 2020;34:100639.
189. Goda JS, Gaikwad U, Narayan A, et al. Primary diffuse large B cell lymphoma of Uterine Cervix: Treatment outcomes of a rare entity with literature review. *Cancer Reports* 2020;3(5):e1264.
190. Demirtas GS, Gokcu M, Sancı M, et al. Primary non-Hodgkin's lymphoma masquerading as cervical cancer. *Ginekologia Polska* 2020;91(9):571-71.
191. Liu G, Shen Z, Yin L, et al. An elderly patient with primary cervical CD5-positive diffuse large B-cell lymphoma: a case report and review of the literature. *European Journal of Gynaecological Oncology* 2020;41(4):500-03.
192. Saeed DM, Patel P, Guzman G, et al. A case of classic Hodgkin lymphoma involving the uterine cervix presenting as vaginal spotting. *Cureus* 2020;12(6)
193. Murata H, Nakamura H, Ohta Y, et al. Two cases of spindle cell variant diffuse large B-cell lymphoma of the uterine cervix. *Gynecologic Oncology Reports* 2020;33:100611.
194. Yoshida Y, Izumi R, Iwashita S, et al. Malignant lymphoma of the cervix in a bicollis uterus considered to be a post-transplant lymphoproliferative disorder in a patient after renal transplantation: A case report. *Gynecologic Oncology Reports* 2020;34:100676.
195. Akkour K, Alhulwah M, Alhalal H, et al. Primary extranodal diffuse large B-cell lymphoma of the uterine cervix. *Malays J Pathol* 2021;43:327-31.
196. Crespo M, Amengual J, Ruiz M, et al. 125 Primary cervico-vaginal non-hodking lymphoma: case report and review: BMJ Specialist Journals, 2021.
197. Desana B, Balbo Mussetto A, Macera A, et al. Rare case of uterine cervix lymphoma with spontaneous regression: Case report. *Journal of Obstetrics and Gynaecology Research* 2021;47(2):807-11.
198. Pons L, Carballas E, Tapia G. Primary diffuse large B cell lymphoma of the cervix. *Medicina Clinica* 2020;156(10):530-31.
199. Birge Ö, Bakır MS, Dinc C, et al. Uterine cervical involvement of non-Hodgkin lymphoma: Rare cause of postcoital bleeding. *Clinical Case Reports* 2021;9(6)

200. Kim S-A, Ahn T-K, Hong R. Primary diffuse large B-cell lymphoma of uterine cervix diagnosed by cytology and concurrent cervical biopsy: a case report and literatures reviews since 1980. *European Journal of Gynaecological Oncology* 2021;42(2):218-28.

201. Sasaki Y, Kemuriyama K, Sasaki S, et al. A Case of Malignant Lymphoma of the Ileum and Uterine Cervix with Perforating Peritonitis of Ileum Tumor Detected during Chemotherapy. *Gan to Kagaku ryoho Cancer & Chemotherapy* 2021;48(9):1157-59.

202. Shim JE, Kim J, Kim M-K, et al. Primary Lymphoma of Cervix. *The Ewha Medical Journal* 2021;44(2):41-45.

203. Capsa C, Calustian LA, Antoniu SA, et al. Primary non-hodgkin uterine lymphoma of the cervix: a literature review. *Medicina* 2022;58(1):106.

204. Stabile G, Sancin L, Boschian Bailo P, et al. Hysteroscopic Resection Followed by Adjuvant Radiotherapy: Report of a New Therapeutic Approach to Primary Cervical Diffuse Large B-Cell Lymphoma. *International Journal of Environmental Research and Public Health* 2022;19(18):11779.

205. Saksena A, Jain A, Pack SD, et al. Follicle center lymphoma (FCL) of the lower female genital tract (LFGT): a novel variant of primary cutaneous follicle center lymphoma (PCFCL). *The American Journal of Surgical Pathology* 2023;47(3):409-19.

206. Alameh T, Mousavi Seresht L, Afshar N, et al. Is Uterine Cervix Lymphoma Missed Most of the Time? A Rare Case of Primary Cervical Lymphoma. *Journal of Obstetrics, Gynecology and Cancer Research (JOGCR)* 2022;7(5):452-57.

207. Aminimoghaddam S, Salarifar E, Noei Teymoordash S. Primary large B-cell lymphoma of the cervix: A case report and review of literature. *Clinical Case Reports* 2022;10(3):e05639.

208. Li J, Zhang X, Liu Y, et al. Primary diffuse large B-Cell lymphoma of the uterine cervix with severe lower urinary tract Symptoms: A rare case report and review of the literature. *Gynecologic Oncology Reports* 2022;43:101066.

209. Rodić DP, Kastratović T, Jovanović D, et al. Lymphoma of the uterine cervix–a rare clinical presentation. *Vojnosanitetski pregled* 2022;79(12)

210. Weng X, Huang M, Zhang M, et al. Primary lymphoma of the uterine cervix: a clinicopathologic study of 13 cases with review of additional 54 cases in the literature. *Virchows Archiv* 2023;482(2):419-29.

211. Seal R, Parkhi M, Kumar R, et al. Primary peripheral T-cell lymphoma of the cervix with mononeuritis multiplex: an unusual case presentation. *Autopsy and Case Reports* 2022;12:e2021354.

212. Shi W, Chen L, Guan D. Lymphoma-like lesion of the uterine cervix: A case report. *Asian journal of surgery* 2023;46(3):1368-69.

213. Gao Y-F, Wang Y, Wang T, et al. A rare case report of primary uterine and vaginal lymphoma in the elderly. *Journal of International Medical Research* 2023;51(3):03000605221147192.
